# Optimizing the Use of Deceased Donor Kidneys at Risk of Discard: A Clinical Practice Guideline

**DOI:** 10.3389/ti.2025.14596

**Published:** 2025-06-26

**Authors:** Joanna C. Dionne, Patricia Campbell, Héloïse Cardinal, Tatiana Giannidis, Aviva Goldberg, S. Joseph Kim, Greg Knoll, Michel Pâquet, Christina Parsons, Yuhong Yuan, Rahul Mainra

**Affiliations:** ^1^ Department of Medicine, McMaster University, Hamilton, ON, Canada; ^2^ Department of Health Research Methods, Evidence, and Impact, McMaster University, Hamilton, ON, Canada; ^3^ Division of Gastroenterology, McMaster University, Hamilton, ON, Canada; ^4^ Department of Laboratory Medicine and Pathology, University of Alberta, Edmonton, AB, Canada; ^5^ Centre de Recherche du Centre Hospitalier de l’Université de Montréal, Montreal, QC, Canada; ^6^ Division of Nephrology, Centre Hospitalier de l’Université de Montréal, Montreal, QC, Canada; ^7^ Department of Pediatrics and Child Health, University of Manitoba Max Rady College of Medicine, Winnipeg, MB, Canada; ^8^ Division of Nephrology, Department of Medicine, University of Toronto, Toronto, ON, Canada; ^9^ Ajmera Transplant Centre, University Health Network, Toronto, ON, Canada; ^10^ Division of Nephrology, Department of Medicine, University of Ottawa and the Ottawa Hospital Research Institute, Ottawa, ON, Canada; ^11^ Canadian Blood Services, Ottawa, ON, Canada; ^12^ Division of Nephrology, Department of Medicine, University of Saskatchewan, Saskatoon, SK, Canada

**Keywords:** kidney transplant, decision making, utilization, organ discard, clinical practice guideline

## Abstract

Underutilization of deceased donor organs has worsened the gap in the number of kidneys available for transplantation. The purpose of this clinical practice guideline is to provide recommendations on the utilization of donor kidneys at risk of discard. Six conditional recommendations were made all with very low certainty of evidence: 1) We suggest utilizing extended criteria donor (ECD) kidneys for transplantation rather than remaining on the wait list and continuing with dialysis; 2) We suggest utilizing kidneys from ECD versus non-ECD in selected transplant candidates; 3) We suggest that organs from older kidney donors can be used in selected transplant candidates who may derive benefit from them; 4) We suggest that kidneys from deceased donors with acute kidney injury can be used for transplantation based on clinician assessment and donor factors; 5) We suggest that donor kidneys with acute kidney injury from either ECD or non-ECD be used for kidney transplantation; 6) We suggest using kidneys from donors after death determination by circulatory criteria for transplantation. This clinical practice guideline provides evidence for the use of deceased donor kidneys that are at risk of discard and may improve the shared decision-making between transplant physicians and wait-listed patients.

## Introduction

Kidney transplantation remains the preferred treatment for patients with end stage kidney disease (ESKD), leading to improved life expectancy and quality of life when compared to remaining on dialysis [1, 2]. Unfortunately, there is a gap in the supply and demand for transplantable organs. Exacerbating this gap is the underutilization of deceased donor kidneys, reported to be as high as 20% of donated kidneys [3]. Meanwhile, patients die while awaiting this lifesaving gift. Despite the availability of compatible deceased donor kidneys, physicians may decline offers on behalf of their patients with the main concern being around kidney quality. These decisions lead to longer wait times, waitlist removal and sometimes death for patients awaiting transplantation [4].

In 2019, Canadian Blood Services convened a Steering Committee of transplant nephrologists, surgeons, and representatives from organ donation organizations (ODOs) to discuss the concern of potential organ (kidney) underutilization in Canada. One main priority identified by this group was to examine the current literature and perform a systematic review to guide evidence-based decision-making at the time of a deceased organ donor offer.

Kidneys at risk of underutilization are generally those with acute kidney injury (AKI), from older donor age, extended criteria donor (ECD) or with high Kidney Donor Risk Index (KDRI). Extended criteria donors are deceased donors aged 60 and above or 50–59 with at least 2 of the following characteristics: history of hypertension, stroke as cause of death or terminal serum creatinine above 132 μmol/L. In a seminal 2002 publication, transplantations of kidneys from ECD were associated with a risk (hazard ratio) of graft loss defined as death, return to dialysis or re-transplantation above 1.7 [5]. The KDRI is a score including donor variables which has been derived from a large retrospective cohort study including all first kidney transplant recipients in the US between 1995 and 2005 [6]. This score is associated with the risk of graft loss and can be expressed as the kidney donor profile index (KDPI), which is the percentile of a donor’s KDRI in the distribution of all donor KDRIs of a reference year. KDPI values are hence comprised between 0% and 100%, with higher values indicating a higher risk of graft loss.

Therefore, we present recommendations on common clinical questions that physicians face when making decisions to accept or decline kidneys that are at risk of discard.

## Materials and Methods

### The Steering Committee

A steering committee (SC) was formed to address optimizing the use of extended criteria deceased organ donors in kidney transplantation. The SC included experts in paediatric and adult transplant nephrology, surgery, ethics and guideline methodology.

### Sponsorship

This guideline was supported and endorsed by Canadian Blood Services.

### Question Development

Priority questions were developed in PICO (Population, Intervention, Comparison and Outcome) format to address evidence for use of extended criteria donor kidneys including, age, presence of acute kidney injury, and donation after death determination by circulatory criteria (DCC, also known as DCD or donation following circulatory death) compared to donation after death determination by neurologic criteria (DNC, also known as NDD or neurological death determination). Questions were developed with the steering committee via teleconferences. Outcomes were then identified and voted upon using an anonymous online voting website. Outcomes were rated as critical [7–9], important [4–6], or limited important [1–3] [7]. Each committee member voted on the outcomes using a modified Delphi approach. Critical outcomes included: mortality, graft survival, graft failure (patient death and graft failure requiring re-transplant or return to dialysis), and quality of life. Important outcomes included: rejection, readmission to hospital, DGF, estimated glomerular filtration rate (eGFR) at 1 year and 3 years, hospital length of stay, infection risk, and malignancy risk.

### Search Strategy and Screening

A search strategy was developed by a medical librarian. We searched MEDLINE, EMBASE, and Cochrane from inception until April 2024. The search strategy also underwent peer review from a second medical librarian. Search terms included: kidney transplant, renal transplant, extended criteria, ECD, acute kidney failure, acute kidney injury, kidney donor, age, infection, neurologic determination of death and donation after cardiac death. The results from the search were uploaded into COVIDENCE [8]. Four reviewers (JCD, YY, CP, TG) screened results for clinical trials, observational studies, and systematic reviews for relevant citations. Title, abstract and full text screening were done in duplicate (JCD, YY, TG). Any disagreement about inclusion at the full text stage was resolved through consensus.

### Data Extraction and Risk of Bias Assessment

Systematic reviews were conducted for each of the PICO questions. Using a standardised pilot data extraction form, the methodology team (JCD, YY, TG) performed data extraction and risk of bias assessment, which in turn were verified by a second reviewer. For clinical trials, the Cochrane Risk of Bias tool 2.0 [9] was used. For observational studies, the Newcastle-Ottawa Risk of Bias [10] assessment tool was employed.

### Data Analysis

For PICO questions that had sufficient data for analysis, a meta-analysis was performed using RevMan version 5.4 [11]. For all outcomes, we calculated fixed and random effects estimates. For PICO questions with less than five studies, we utilised a fixed effects model. For most PICO questions, a random effects model was used. For dichotomous outcomes, we reported a relative risk (RR) or odds ratios (OR). For continuous variables, a mean difference (MD) was used. All effect size measures are reported with 95% confidence intervals (CI). For PICO questions where there was insufficient data to allow for meta-analysis, the evidence was synthesised narratively.

The certainty of evidence was assessed using the Grading of Recommendations, Assessment, Development and Evaluation (GRADE) process [12]. In accordance with GRADE, we rated the certainty of evidence for each outcome as high, moderate, low or very low [13]. We rated the certainty of evidence for each outcome as high if data were from randomized controlled trials (RCTs) and low if data were from observational studies. The data were rated down 1 or 2 levels if the results were at serious, or very serious risk of bias, if there were serious inconsistencies across studies, if the evidence was indirect, or if there were concerns regarding publication bias. Data were rated up in observational studies if there were large effect sizes or dose-response gradients.

### Evidence Summary and Recommendation Formulation

Evidence summaries for each of the PICO questions (Supplementary Material S1) were developed by the methodologists (JCD, YY) including information on the methodology of each study, population, interventions, pooled estimates for each outcome, and overall rating of the certainty of evidence. Evidence to decision (EtD) frameworks (Supplementary Material S2) were completed by the SC to draft recommendations considering certainty of the evidence, balance of desirable and undesirable effects, resources required, feasibility, acceptability, and equity. Recommendations were considered approved if at least 80% of the committee agreed with the statement.

## Guidelines

### Part 1: The Evidence for the Use of Extended Criteria Donor Kidneys

#### Recommendation 1: We Suggest Utilizing ECD Kidneys for Transplantation Rather than Remaining on the Wait List and Continuing with Dialysis (Conditional Recommendation, Very Low Certainty of Evidence)

##### Evidence Summary

Transplantation with kidneys from ECD are associated with a lower risk of mortality (pooled RR 0.88, 95% CI 0.84, 0.92) [14, 15] when compared with remaining on dialysis.

##### Justification

Although kidneys from ECD are associated with a moderately higher risk of graft loss than those from non-ECD, the decision to accept or decline such kidneys must be made by comparing the benefits and risks of transplantation versus remaining on dialysis waiting for a better offer. Our systematic review of the literature and meta-analysis show that transplantation with kidneys from ECD is associated with lower mortality than remaining on dialysis (pooled RR 0.88, 95% CI 0.84, 0.92) [14, 15]. In the largest study supporting this result, the survival benefit was seen in recipients aged more than 40, especially in areas with long wait times [14].

Due to heterogeneity in KDPI categories and outcomes in relevant studies, we could not perform a meta-analysis of the data expressing transplant outcomes according to donor KDPI. Nevertheless, we identified one large retrospective cohort study which provided results similar to those presented in our systematic review for ECD. This study showed that accepting a kidney from a donor with high KDPI was associated with a survival benefit compared with remaining on dialysis [16].

The quality of evidence is very low as a result of the high risk of bias given the retrospective nature of the studies and the inconsistencies in results between studies.

##### Implementation

Kidneys from ECD are already utilized in clinical practice. Implementation considerations include identifying ways to operationalize wider utilization, promote transplant physician and candidate education as to the survival benefit of transplantation with kidneys from ECD to improve shared decision-making when kidneys from ECD are offered.

##### Research Priorities

Impact of transplantation with kidneys from ECD on cost effectiveness of the procedure and quality of life of kidney transplant candidates and recipients are needed. A provider preferences survey including conjoint analysis with transplant recipients could help determine the elements driving decision-making for accepting or declining kidneys from ECD. Better data are needed to determine which patients are the best candidates to receive ECD kidneys and efforts should be made to implement more consistent practices across the country.

#### Recommendation 2: We Suggest Utilizing Kidneys from ECD Versus Non-ECD in Selected Transplant Candidates (Conditional Recommendation, Very Low Certainty of Evidence)

##### Evidence Summary

Transplantation with kidneys from ECD versus non-ECD is associated with a moderately increased risk of mortality [14, 17–28], graft loss, DGF, and a small decrease in death-censored graft survival. There were no differences in the incidence of acute rejection, and hospital readmissions in transplantations performed with kidneys from ECD versus non-ECD.

##### Justification

Our systematic review of the literature and meta-analysis indicate that the risk of mortality (pooled RR 1.5, 95% CI 1.25, 1.80) [17–19, 21–26, 28–36], graft loss (pooled RR 1.63, 95% CI 1.32, 2.02) [18, 27, 28, 35, 37–39] and DGF (pooled RR 1.23, 95% CI 1.04, 1.46 [17–19, 21–26, 31, 35, 37–70] were moderately increased when transplantation was performed with kidneys from ECD versus non-ECD, while death-censored graft survival was only mildly improved when non-ECD were transplanted as compared with ECD (pooled RR 0.95, 95% CI 0.90, 0.99) [14, 26, 36, 42, 64–66, 71]. The risk of acute rejection (pooled RR 1.10, 95% CI 0.89, 1.37) [17–19, 21–24, 26, 31, 32, 35, 37–41, 45, 46, 48, 50, 52, 53, 55–57, 61, 63, 65, 66, 72–77] and hospital readmission (pooled RR 1.17, 95% CI 0.71, 1.92) [32, 37, 38, 62, 66, 67, 76] were not different when transplantation with kidneys from ECD was compared with transplantation with kidneys from non-ECD.

We performed a literature search to identify transplant outcomes by donor quality when the latter was expressed as the KDPI. Due to heterogeneity in KDPI cut-offs that define risk donor categories across studies, we were unable to perform a meta-analysis on the data. Nevertheless, the results of studies comparing transplantations with kidneys from donors with high versus low KDPI were similar to those comparing outcomes of transplantation in patients receiving ECD versus non-ECD. Transplantation with kidneys from donors with high KDPI reported a higher risk of graft loss and DGF compared with kidneys from donors with lower KDPI values.

The quality of the evidence was very low due to limitations in study design (observational studies only), the risk of selection and confounding biases due to differences in recipient characteristics among those who get offered kidneys from ECD, and double counting across studies using similar databases.

##### Implementation

Kidneys from ECD are already utilized in clinical practice. Implementation considerations are similar to Recommendation 1, Part 1. Ultimately identifying patients that will benefit from accepting an ECD kidney transplant compared to remaining on dialysis is imperative in wider utilization.

##### Research Priorities

Studies that address the barriers and facilitators that promote wider utilization should be performed, as well as studies that evaluate the impacts of using more ECD on cost-effectiveness and quality of life of transplant candidates and recipients. Research is also required to better understand which potential transplant candidates will benefit the most from these kidneys vs. remaining on dialysis.

#### Recommendation 3: We Suggest that Organs from Older Kidney Donors Can be Used in Selected Transplant Candidates Who May Derive Benefit from them (Conditional Recommendation, Very Low Certainty of Evidence).

##### Evidence Summary

The data supporting the recommendations were derived from non-randomised (i.e., observational) studies. The studies span the spectrum in scope, ranging from single centre reports to large national registry analyses. In addition, follow-up varied across studies from 1, 3, 5, to 10 years. Most studies dichotomized “younger” vs. “older” deceased donors as < 65 years vs. >/ = 65 years. It was acknowledged by the panel that age cut-offs used in these analyses are somewhat arbitrary, but 65 years of age seemed to be a widely adopted threshold for categorising the deceased donor population.

The pooled data showed that kidneys from younger (vs. older) deceased donors were generally associated with a higher relative likelihood of patient survival (RR 0.95, 95% CI: 0.93, 0.98) [21, 56, 78–97] and graft survival (RR 0.88, 95% CI: 0.86, 0.91) [21, 56, 78–80, 82–85, 87–89, 92, 98–113]. Furthermore, recipients of kidneys from older donors were more likely to experience DGF (RR 1.29, 95% CI: 1.12, 1.48) [78, 80–84, 86, 87, 89–92, 97, 98, 111, 113–124] and acute rejection (RR 1.18, 95% CI: 1.02, 1.37) [78, 82, 83, 90, 92, 96, 98, 99, 107, 115, 119, 122, 123, 125] when compared to recipients of kidneys from younger donors. Despite these differences, it was noted by the panel members that these differences were relatively small and other outcomes such as death-censored graft survival were comparable across the two groups (RR 0.97, 95% CI: 0.94, 1.00) [21, 78, 82, 84, 86, 87, 90–92, 94, 97, 117, 122].

The overall certainty of the evidence was very low given that the estimates were derived from comparisons in donor age groups with varying degrees of control for confounding and selection biases. Variation in the application of age cut-offs and the decision to dichotomize a continuous variable such as donor age to facilitate presentation of the results may have led to measurement bias.

##### Justification

Although the comparison of recipient outcomes between kidney transplanted from younger vs. older donors favoured younger donors in various domains (including graft survival), it was highlighted by the panel members that the net benefit of using kidneys from older donors in appropriate recipient candidates may provide substantial benefit at a system level in light of their broad availability, the continued mismatch between supply and demand for organs, and the relatively small differences in recipient outcomes between kidney transplants from younger vs. older donors. It is notable that the certainty of the evidence is very low given the risk for bias in the outcome estimates from observational studies.

The included studies provided no information on the cost-effectiveness of different strategies for using younger vs. older donor kidneys nor did they explicitly address issues of equity. Given that kidneys from a wide spectrum of donor ages are already being used in clinical practice, the panel felt that developing strategies to optimise the use of older donor kidneys would be both acceptable and feasible.

##### Implementation

The panel highlighted that donor age is a continuum and thus implementation of policies around the use of these kidneys should consider the impact of extremes of age on recipient outcomes. Other implementation considerations include defining the best approach to operationalizing the use of kidneys from a wider age spectrum, including the integration of shared decision-making frameworks that account of patient and provider preferences.

##### Research Priorities

One main research priority is being able to identify which recipients will benefit from transplantation with older donor kidneys. Future studies should establish the quality of life of recipients receiving older vs. younger donor kidneys. Furthermore, it would be important to establish the cost-effectiveness of strategy that expands the use of kidneys from older donors. Research on ways to modify and improve the function as well as long-term outcomes of older donor kidneys should be a priority. These studies may include interventions on donors and/or recipients as well as the rational application of biomarkers in supporting the management of the organ and patient.

### Part 2: The Evidence for the Use of Kidneys With Acute Kidney Injury

#### Recommendation 1: We Suggest that Kidneys from Deceased Donors with AKI Can be Used for Transplantation Based on Clinician Assessment and Donor Factors (Conditional Recommendation, Very Low Certainty of Evidence).

##### Evidence Summary

For the comparison of outcomes of renal transplantation using kidneys from deceased donors who had an acute kidney injury vs. deceased donors who did not (non-AKI), only observational studies were included in the analysis. Thereby, the risk of bias is high. Outcomes that were analysed included mortality and graft survival at different time points, and acute rejection up to a year. Graft survival at 1 year in 12 observational studies including over 16,000 patients was lower in donors with AKI: (RR 1.14, 95% CI: 1.02, 1.27) [22, 26, 35, 126–134]. No difference was found in graft loss at 3 years (RR 1.03, 95% CI: 0.84, 1.26) [56, 127, 128]. Mortality was similar at varying time points from 1 to 6 years (RR 0.80, 95% CI: 0.56, 1.14) [26, 35, 56, 127, 131–137]. No significant difference was found in the rate of DGF when the deceased donor had AKI (RR 1.53, 95% CI: 0.88, 2.68) [26, 35, 50, 53, 127–129, 131–154], and little to no difference was found in the rate of acute rejection up to 1 year after transplant as a function of deceased donor AKI status (RR 1.02, 95% CI: 0.94, 1.11) [26, 53, 127, 131–134, 138–142, 144, 149, 150, 152, 155].

##### Justification

No worrisome signal was seen in outcomes from kidneys whose donor had AKI. In particular, there was no difference in graft survival up to 10 years post-transplant, no difference in mortality up to 5 years and no difference in acute rejection at 1 year. Therefore, we suggest that kidneys from donors with AKI can be used for transplantation. However, this is based on very low certainty of evidence. Of note, severity of AKI (stage 1, 2 or 3) or the need for renal replacement therapy in deceased donors was not considered in these observational studies. Moreover, the studies could only ascertain the outcomes of AKI kidneys that were transplanted. The factors distinguishing AKI kidneys that were declined vs. used (and the outcomes of the former if they did get used for transplantation) are not clear. Having said this, using kidneys from deceased donors with AKI is both feasible and acceptable under the appropriate settings.

##### Implementation

The relative effect of using kidneys from deceased donors with AKI on graft loss is trivial. These kidneys are already being used by some transplant programs, but more widespread use should be encouraged. Implementation of the widespread use of donors with AKI will require improved education of accepting physicians on the positive outcomes of these specific kidneys. Furthermore, awareness of critical care and donor physicians that such kidneys may be donated is needed. Although not universal, some physicians do request a reassuring biopsy prior to accepting these organs. Resources to increase access of timely renal biopsy results may also be required, however this was not studied by our group.

##### Research Priorities

The very low certainty of evidence points to a research priority assessing the efficacy and the safety of using kidneys from deceased donors with AKI for kidney transplantation. Such future multicentre studies should use a standardised definition of AKI, stage of AKI, cause of AKI, and whether donor kidney replacement therapy was needed. Outcomes in different transplant candidate subgroups might identify specific transplant candidates who would benefit most from these kidneys.

#### Recommendation 2: We Suggest that Donor Kidneys with AKI from Either ECD or Non-ECD be Used for Kidney Transplantation (Weak Recommendation, Very Low Certainty of Evidence)

##### Evidence Summary

A total of six observational studies were found in the literature [22, 26, 50, 53, 126, 137]. The definition of AKI was not standardized and varied from between studies. Overall, there was no statistically significant differences in any of the outcomes of interest between donor kidneys with AKI labelled as ECD or non-ECD. There was a small difference in overall graft survival with a RR 1.1 (95% CI 1.0–1.2) favouring non-ECD kidneys with AKI [26, 53, 55]. However, there was no difference in mortality [22, 26, 134], DGF [22, 26, 50, 53, 134, 137], acute rejection [26, 50, 53, 135, 137] or eGFR [22, 26, 50, 53] at 1 year.

##### Justification

The available studies suggest that donors with AKI have similar outcomes regardless of ECD or non-ECD status. Given the lack of standardization of AKI definition and the uncertainty around the use of renal biopsy prior to transplantation, this is a weak recommendation. The outcome measures do not suggest any significant harm from transplanting kidneys from ECD with AKI. It is therefore feasible that this category of donor kidneys can be transplanted safely in selected recipients. None of the studies discussed cost utilization given the potential for DGF, although the data did not suggest a higher risk in ECD with AKI.

##### Implementation

It is possible that ECD with AKI make up a large proportion of underutilized organs. Appropriate use of these organs can lead to increased access to transplantation for selected patients with ESKD. Improving knowledge and education along with shared decision making between clinicians and patients is important to consider in the implementation of this strategy. Other implementation concerns are highlighted in Recommendation 1, Part 2.

##### Research Priorities

The evidence guiding this recommendation is of very low certainty and there is opportunity to inform the transplant community with well-designed studies using a standardized definition of AKI. Biopsy practices vary and additional research could focus on biopsy results and transplant outcomes. Other research questions are mentioned in Recommendation 1, Part 2.

### Part 3: The Evidence for the Use of Kidneys From Donors After Circulatory and Neurologic Determination of Death

#### Recommendation 1: We suggest Using Kidneys from Donors After Death Determination by Circulatory Criteria for Transplantation (Conditional Recommendation, Very Low Certainty of Evidence)

##### Evidence Summary

The literature comparing recipient outcomes of kidneys transplanted from donors after DNC vs. DCC are based on observational studies from single-centre, multicentre, and national data sources. There are no randomised controlled trials that have established the comparative effectiveness of these two approaches. DCC kidney transplants have seen a resurgence over the last 20 years due to advances in surgical recovery techniques, organ preservation, and the continued gap between the supply and demand for transplantable organs.

The evidence synthesis showed that recipients of DCC kidneys experienced a higher risk of all-cause mortality (RR 1.33, 95% CI: 1.15, 1.54) [156–164] and graft loss (RR 1.08, 95% CI: 1.00, 1.17) [158–161, 163–165] when compared to recipients of DNC kidneys. The relative likelihood of death-censored graft loss was comparable between the two groups (RR 1.04, 95% CI: 0.92, 1.17) [159, 162]. Recipients of DCC kidney transplants had an 89% increase in the risk of DGF when compared to those who received a DNC kidney transplant (RR 1.89, 95% CI: 1.80, 1.99) [19, 156, 158, 160, 162, 163, 166–169]. Although the point estimate for acute rejection suggested a 62% increased risk among DCC (vs. DNC) kidney recipients, the level of precision did not allow for definitive conclusions (RR 1.62, 95% CI: 0.77, 3.42) [19, 157, 160, 163, 167, 169, 170].

Similar to other analyses that compare recipient outcomes across specific donor characteristics, the overall certainty of the evidence was very low due to the estimates being derived from comparisons that vary in terms of bias control for confounding and selection. The inclusion of studies derived from the same data sources (e.g., national registry) may lead to double counting of subjects and events, leading to measurement bias.

##### Justification

There was a moderately desirable effect of DNC over DCC kidney transplants, particularly in the domains of mortality and delayed graft function. DCC kidney transplants clearly had a notably higher relative likelihood of DGF, which is consistent with the mechanism of donation (i.e., longer warm ischemia time in DCC than DNC). Of note, differences in graft loss and acute rejection were null or trivial. This supports the recommendation to explore ways to increase the use DCC kidneys, when available, in patient groups that may benefit from them.

##### Implementation

DCC kidney transplants are being used in many organ donation and transplantation systems across the world but there may be opportunities to expand their deployment in certain countries, regions, and jurisdictions. One must also consider the resources/costs associated with properly undertaking life-support discontinuation, donor monitoring, and rapid preservation/recovery of organs after cessation of circulation in the donor. DCC kidney recipients are almost two-fold more likely to experience DGF, which has implications of inpatient dialysis services and length of stay.

##### Research Priorities

Reliable methods to predict death following discontinuation of life-support will support the rational allocation of resources to optimise the availability of DCC organs for transplantation. Moreover, research on ways to safely extend the time from withdrawal of life-sustaining therapies until death and techniques to improve organ viability (with reductions in DGF rates) should be prioritised.

## Discussion

Organ utilization is a primary concern for a wide spectrum of healthcare users, providers, and payers. Kidney transplantation provides patients with an improved quality of life and overall life expectancy compared to remaining on dialysis and is the preferred treatment for ESKD. Not only is there clinical benefit for patients, but transplantation leads to considerable cost savings [171, 172]. Kidney underutilization exacerbates the organ shortage problem facing patients with ESKD, increasing wait times, and reducing access to transplantation. Furthermore, the public may lose trust in the organ donation and transplant systems if utilization of this precious gift is not optimized.

Clinicians are faced with the difficult decision of accepting or declining a kidney that is non-standard criteria. This clinical practice guideline summarizes the available evidence on the outcomes of non-standard criteria donor kidneys, labelled as either ECD, older age, DCC or those with AKI. We present the recommendations of six PICOs that may assist in clinical decision making. There was ample evidence in the literature, however given the limitations of the data, only conditional recommendations could be provided for all PICOs. The nature of retrospective, registry data with the potential for double-counting leads to the potential for bias, which limits the strength of these recommendations.

It is important to appreciate that the results of all these studies includes kidneys that were ultimately transplanted. There are likely many kidneys that are not transplanted which outcomes are unknown. One could assume that if all kidneys were transplanted, outcomes may be worse with non-standard criteria donor kidneys. However, we are not advocating that all kidneys are transplanted but appropriate clinical decision making to identify those that will provide patients with improved outcomes. We feel confident that our data are inclusive of all relevant literature up to April of 2024. We undertook rigorous methodological assessments following well established GRADE criteria to determine the strength of the data. Lastly, our steering committee did not have a patient partner that was guiding our PICO question development and determination of outcomes that are important to patients. However, we do believe that these are important questions that carry importance to our patients.

Overall, the majority of studies show small or trivial differences in outcomes of importance when transplanting a kidney from an older donor, ECD, DCC or those donors suffering AKI. Although some studies revealed the potential for poorer outcomes, we believe the limitations of the data call to question these results. Ultimately this decision must balance the risk of potential negative outcomes to the benefit of improved life expectancy and quality of life with a transplant compared to remaining on dialysis.

A recent study published after our search was complete present a systematic review of the literature on the outcomes of kidney transplantation from donors with AKI. Overall, their results were in concordance with ours when investigating specifically donors with AKI and graft outcomes. They do present a few studies that highlighted the impact of AKIN stages on graft outcomes. Only stage 3 AKI portended a poorer prognosis with higher rates of DGF, but this did not lead to worse long term graft outcomes. They also highlighted the differences in the aetiology of AKI and its impact on graft outcomes comparing hemodynamic, intrinsic and mixed causes. Only intrinsic AKI resulted in higher rates of DGF and a lower 1-year eGFR [173].

This guideline is only one component in our attempts to improved kidney utilization. The importance of patient knowledge and education cannot be understated. Patients awaiting transplantation must have easy to understand education provided in a time of low stress, in other words not at the time of an organ offer. Prior work from our group has confirmed this and suggested that most elderly patients are willing to accept a kidney transplant with reduced long-term outcomes to provide them freedom from dialysis for 3–5 years [174]. Identifying the right recipient for the right kidney can lead to improved patient outcomes and improved organ utilization. Ultimately as we transplant more kidneys that are at risk of underutilization, we must accept as a community the potential for reduced long-term outcomes. Individual programs that are focused on performance indicators such as long-term graft survival may have to shift their priority to other markers of success. Furthermore, the utilization of non-standard kidneys into an ever-increasing complex patient population may also lead to higher healthcare needs and the financial costs associated with it. To address this adequately, high quality prospective research is required.

There are other clinical factors which have an impact on organ underutilization. There is robust evidence that hypothermic machine perfusion (HMP) reduces the risk of DGF in deceased donor kidney transplantation [175]. Graft survival was also improved with the use of HMP in some but not all studies. An interesting pharmacoeconomic analysis proposed that the use of HMP for ECD kidneys led to an improvement in utilization with a higher number of transplants. Overall costs were higher but in their proposed scenario of HMP for ECD kidneys and static cold storage for SCD kidneys, there were cost savings realized on the fifth year [176]. Other reports also document the potential for a lower discard rate with the use of HMP [177, 178]. Despite the limited evidence that histological findings on recovered deceased donor kidneys is associated with graft outcomes, procurement biopsy for ECD kidneys is a common practice [179]. There is a strong association between biopsy findings and organ discard rate with the degree of glomerulosclerosis (>20%) and macroscopic arteriosclerosis being important histological findings affecting decision making [180, 181]. If procurement biopsies are a common practice for ECD kidneys to assist with decision making, utilizing standardized clinical pathological scores may improve utilization. Zhang and colleagues developed a kidney donor quality score (KDQS) based on deep learning assessment of procurement biopsies [182]. The KDQS in addition to clinical covariates predicted one- and five-year graft loss with an area under the curve of 0.70 and 0.64, respectively, and thus has the potential to reduce kidney discards. Lastly, dual kidney transplantation may improve utilization of ECD kidneys that would otherwise be discarded. Donor kidneys that are at risk of discard based on histological or clinical criteria can be successfully transplanted using an algorithm decision tree [183]. High risk donors based on clinical and histological criteria underwent dual kidney transplantation resulting in similar graft survival as single kidney transplantation from similar donors but lower biopsy lesions.

We highlight several research priorities and unmet needs in this topic. Further research is required to determine cost effectiveness of this strategy, patient quality of life, and more accurate ways of identifying characteristics of recipients and donor kidneys to improve overall outcomes. This clinical guideline has the potential to increase kidney utilization reducing the gap of organ shortage for patients with ESKD.

## Data Availability

The original contributions presented in the study are included in the article/Supplementary Material, further inquiries can be directed to the corresponding author.

## Abbreviations

AKI, acute kidney injury; AKIN, acute kidney injury network; CI, confidence interval; DCC, death determination by circulatory criteria; DCD, donation following circulatory death; DGF, delayed graft function; DNC, death determination by neurological criteria; ECD, extended criteria donor; eGFR, estimated glomerular filtration rate; ESKD, end stage kidney disease; EtD, evidence to decision; GRADE, Grading of Recommendations, Assessment, Development and Evaluation; HMP, hypothermic machine perfusion; KDPI, kidney donor profile index; KDQS, kidney donor quality score; KDRI, kidney donor risk index; NDD, neurological death determination; ODO, organ donation organizations; OR, odds ratio; MD, mean difference; PICO, Population, Intervention, Comparison and Outcome; RCTs, randomized controlled trials; RR, relative risk; SC, steering committee.
