# Supplementary material for: Optimizing the Use of Deceased Donor Kidneys at Risk of Discard: A Clinical Practice Guideline
Source: Transpl Int. 2025 Jun 26;38:14596. doi: 10.3389/ti.2025.14596 (PMC12240869; doi:10.3389/ti.2025.14596)
Supplement: Supplementary file 1 [file DataSheet2.PDF]

QUESTION

Should ECD vs. Waitlist be used for renal transplant in ESRD?	
**POPULATION:**	renal transplant in ESRD
**INTERVENTION:**	ECD
**COMPARISON:**	Waitlist
**MAIN OUTCOMES:**	Mortality;
**SETTING:**	
**PERSPECTIVE:**	
**BACKGROUND:**	
**CONFLICT OF INTERESTS:**	

ASSESSMENT

Problem
Is the problem a priority?

JUDGEMENT	RESEARCH EVIDENCE	ADDITIONAL CONSIDERATIONS
○ No<br>○ Probably no<br>○ Probably yes<br>● Yes<br>○ Varies<br>○ Don't know	*See Appendix 2*	

Desirable Effects
How substantial are the desirable anticipated effects?

JUDGEMENT	RESEARCH EVIDENCE	ADDITIONAL CONSIDERATIONS
○ Trivial<br>○ Small<br>○ Moderate<br>● Large<br>○ Varies<br>○ Don't know	*See Appendix 1*	

Undesirable Effects
How substantial are the undesirable anticipated effects?

JUDGEMENT	RESEARCH EVIDENCE	ADDITIONAL CONSIDERATIONS

○ Large<br>○ Moderate<br>○ Small<br>○ Trivial<br>● Varies<br>○ Don't know		

Certainty of evidence

What is the overall certainty of the evidence of effects?

JUDGEMENT	RESEARCH EVIDENCE	ADDITIONAL CONSIDERATIONS
● Very low<br>○ Low<br>○ Moderate<br>○ High<br>○ No included studies	*See Appendix 1*	

Values

Is there important uncertainty about or variability in how much people value the main outcomes?

JUDGEMENT	RESEARCH EVIDENCE	ADDITIONAL CONSIDERATIONS
○ Important uncertainty or variability<br>○ Possibly important uncertainty or variability<br>● Probably no important uncertainty or variability<br>○ No important uncertainty or variability	Balance of mortality versus quality of life differences.	

Balance of effects

Does the balance between desirable and undesirable effects favor the intervention or the comparison?

JUDGEMENT	RESEARCH EVIDENCE	ADDITIONAL CONSIDERATIONS

○ Favors the comparison<br>○ Probably favors the comparison<br>○ Does not favor either the intervention or the comparison<br>● Probably favors the intervention<br>○ Favors the intervention<br><br>○ Varies<br>○ Don't know	Favour receiving a ECD renal transplant versus staying on the weight list.	

Resources required
How large are the resource requirements (costs)?

JUDGEMENT	RESEARCH EVIDENCE	ADDITIONAL CONSIDERATIONS
○ Large costs<br>○ Moderate costs<br>○ Negligible costs and savings<br>○ Moderate savings<br>● Large savings<br><br>○ Varies<br>○ Don't know	Evidence for transplant likely leads to cost savings. Improvement in QUALYS.	Braden Manns -paper

Certainty of evidence of required resources
What is the certainty of the evidence of resource requirements (costs)?

JUDGEMENT	RESEARCH EVIDENCE	ADDITIONAL CONSIDERATIONS
○ Very low<br>○ Low<br>● Moderate<br>○ High<br><br>○ No included studies	Most studies did not examine comparator ECD versus remaining on the waitlist and continuing with dialysis.<br>Indirect evidence.<br>Staying on dialysis estimate cost is $70,000/year<br>Transplant in first year cost is 100, 000/year then subsequent years cost decreases.	Add in specific CBS data.

Cost effectiveness
Does the cost-effectiveness of the intervention favor the intervention or the comparison?

JUDGEMENT	RESEARCH EVIDENCE	ADDITIONAL CONSIDERATIONS

○ Favors the comparison<br>○ Probably favors the comparison<br>○ Does not favor either the intervention or the comparison<br>● Probably favors the intervention<br>○ Favors the intervention<br>○ Varies<br>○ No included studies	Most studies did not examine comparator ECD versus remaining on the waitlist and continuing with dialysis.<br>Indirect evidence.<br>Staying on dialysis estimate cost is $70,000/year<br>Transplant in first year cost is $100, 000/year then subsequent years cost decreases.	Caveat gap in literature pertaining specifically to ECD.

Equity
What would be the impact on health equity?

JUDGEMENT	RESEARCH EVIDENCE	ADDITIONAL CONSIDERATIONS
○ Reduced<br>○ Probably reduced<br>○ Probably no impact<br>○ Probably increased<br>○ Increased<br>● Varies<br>○ Don't know	Potentially could be geographical barriers to access	

Acceptability
Is the intervention acceptable to key stakeholders?

JUDGEMENT	RESEARCH EVIDENCE	ADDITIONAL CONSIDERATIONS
○ No<br>○ Probably no<br>○ Probably yes<br>○ Yes<br>● Varies<br>○ Don't know	May be variation in acceptability of using differnt types of ECD kidneys from a clinician standpoint.<br>From a patient prospective would be acceptable intervention.	

Feasibility
Is the intervention feasible to implement?

JUDGEMENT	RESEARCH EVIDENCE	ADDITIONAL CONSIDERATIONS
○ No<br>○ Probably no<br>● Probably yes<br>○ Yes<br>○ Varies<br>○ Don't know	ECD transplantation is already being performed. However, would be increased costs with increased uptake of ECD from administrative and logistic standpoint.	

SUMMARY OF JUDGEMENTS

	JUDGEMENT						
PROBLEM	No	Probably no	Probably yes	**Yes**		Varies	Don't know
DESIRABLE EFFECTS	Trivial	Small	Moderate	**Large**		Varies	Don't know
UNDESIRABLE EFFECTS	Large	Moderate	Small	Trivial		**Varies**	Don't know
CERTAINTY OF EVIDENCE	**Very low**	Low	Moderate	High			No included studies
VALUES	Important uncertainty or variability	Possibly important uncertainty or variability	**Probably no important uncertainty or variability**	No important uncertainty or variability			
BALANCE OF EFFECTS	Favors the comparison	Probably favors the comparison	Does not favor either the intervention or the comparison	**Probably favors the intervention**	Favors the intervention	Varies	Don't know
RESOURCES REQUIRED	Large costs	Moderate costs	Negligible costs and savings	Moderate savings	**Large savings**	Varies	Don't know
CERTAINTY OF EVIDENCE OF REQUIRED RESOURCES	Very low	Low	**Moderate**	High			No included studies
COST EFFECTIVENESS	Favors the comparison	Probably favors the comparison	Does not favor either the intervention or the comparison	**Probably favors the intervention**	Favors the intervention	Varies	No included studies
EQUITY	Reduced	Probably reduced	Probably no impact	Probably increased	Increased	**Varies**	Don't know
ACCEPTABILITY	No	Probably no	Probably yes	Yes		**Varies**	Don't know
FEASIBILITY	No	Probably no	**Probably yes**	Yes		Varies	Don't know

TYPE OF RECOMMENDATION

Strong recommendation against the intervention	Conditional recommendation against the intervention	Conditional recommendation for either the intervention or the comparison	Conditional recommendation for the intervention	Strong recommendation for the intervention
○	○	○	●	○

CONCLUSIONS

Recommendation

We suggest transplanting with ECD over remaining on the waitlist and continuing with dialysis (conditional recommendation, very low certainty of evidence).

Justification

Subgroup considerations

Implementation considerations

Monitoring and evaluation

Research priorities

Specific cost effectiveness studies on ECD use compared to remaining on the

APPENDICES

Appendix 1

Outcomes	№ of participants (studies) Follow-up	Certainty of the evidence (GRADE)	Relative effect (95% CI)	Anticipated absolute effects* (95% CI)	
				Risk with Waitlist	Risk difference with ECD
Mortality	117112<br>(2 observational studies)	⨁◯◯◯<br>Very low [a,b]	**RR 0.88**<br>(0.84 to 0.92)	Study population	
				259 per 1,000	**31 fewer per 1,000**<br>(41 fewer to 21 fewer)

a. Rated down due to high risk of bias amongst studies.
b. Rated down for inconsistency amongst results across studies.

Appendix 2

Outcomes	Anticipated absolute effects[*] (95% CI)		Relative effect (95% CI)	№ of participants (studies)	Certainty of the evidence (GRADE)	Comments
	Risk with Waitlist	Risk with ECD				
Mortality	Study population		**RR 0.88** (0.84 to 0.92)	117112 (2 observational studies)	⊕◯◯◯ Very low [a,b]	
	259 per 1,000	**228 per 1,000** (218 to 239)				

a. Rated down due to high risk of bias amongst studies.
b. Rated down for inconsistency amongst results across studies.

QUESTION

Should ECD kidneys vs. SCD kidneys be used for renal transplant?	
POPULATION:	renal transplant
INTERVENTION:	Update ECD kidneys
COMPARISON:	SCD kidneys
MAIN OUTCOMES:	Mortality (Overall); Acute Rejection; Delayed graft function; Graft Survival (Overall); Death Censored Graft Survival;
SETTING:	
PERSPECTIVE:	
BACKGROUND:	
CONFLICT OF INTERESTS:	

ASSESSMENT

Problem<br>Is the problem a priority?		
JUDGEMENT	RESEARCH EVIDENCE	ADDITIONAL CONSIDERATIONS
○ No<br>○ Probably no<br>○ Probably yes<br>● Yes<br>○ Varies<br>○ Don't know		
**Desirable Effects**<br>How substantial are the desirable anticipated effects?		
JUDGEMENT	RESEARCH EVIDENCE	ADDITIONAL CONSIDERATIONS
○ Trivial<br>○ Small<br>○ Moderate<br>○ Large<br>● Varies<br>○ Don't know	*See Appendix 2*	With respect to mortality, not all studies age adjusted recipient age. Moderate desirable effect of decreased mortality with SCD.<br><br>With acute rejection, delated graft function, graft survival and death censored graft survival, the effects are small.
**Undesirable Effects**<br>How substantial are the undesirable anticipated effects?		
JUDGEMENT	RESEARCH EVIDENCE	ADDITIONAL CONSIDERATIONS

○ Large<br>○ Moderate<br>○ Small<br>○ Trivial<br>● Varies<br>○ Don't know	*See Appendix 1*	

Certainty of evidence

What is the overall certainty of the evidence of effects?

JUDGEMENT	RESEARCH EVIDENCE	ADDITIONAL CONSIDERATIONS
● Very low<br>○ Low<br>○ Moderate<br>○ High<br>○ No included studies	Concerns re risk of bias with use of databases, potential double counting of patients, as well as different definitions used across studies.	

Values

Is there important uncertainty about or variability in how much people value the main outcomes?

JUDGEMENT	RESEARCH EVIDENCE	ADDITIONAL CONSIDERATIONS
○ Important uncertainty or variability<br>○ Possibly important uncertainty or variability<br>● Probably no important uncertainty or variability<br>○ No important uncertainty or variability	Probably no important uncertainty or variability in terms values patients, providers and policy makes.	

Balance of effects

Does the balance between desirable and undesirable effects favor the intervention or the comparison?

JUDGEMENT	RESEARCH EVIDENCE	ADDITIONAL CONSIDERATIONS

● Favors the comparison<br>○ Probably favors the comparison<br>○ Does not favor either the intervention or the comparison<br>○ Probably favors the intervention<br>○ Favors the intervention<br><br>○ Varies<br>○ Don't know	*See Appendix 1*	In terms of mortality does favour SCD. When looking at differences between SCD and ECD in terms of acute rejection, delayed graft function, graft survival and death censored graft survival difference between ECD and SCD is small to trivial.<br><br>However still overall favors SCD.
**Resources required**<br>How large are the resource requirements (costs)?		
**JUDGEMENT**	**RESEARCH EVIDENCE**	**ADDITIONAL CONSIDERATIONS**
○ Large costs<br>○ Moderate costs<br>○ Negligible costs and savings<br>○ Moderate savings<br>○ Large savings<br><br>○ Varies<br>● Don't know	No formal studies included regard cost. However, if increased utilization of ECD kidneys, there is a potential cost of longer hospitalization, and the need for dialysis post transplant in the setting of delayed graft function.	
**Certainty of evidence of required resources**<br>What is the certainty of the evidence of resource requirements (costs)?		
**JUDGEMENT**	**RESEARCH EVIDENCE**	**ADDITIONAL CONSIDERATIONS**
○ Very low<br>○ Low<br>○ Moderate<br>○ High<br><br>● No included studies		
**Cost effectiveness**<br>Does the cost-effectiveness of the intervention favor the intervention or the comparison?		
**JUDGEMENT**	**RESEARCH EVIDENCE**	**ADDITIONAL CONSIDERATIONS**

○ Favors the comparison<br>○ Probably favors the comparison<br>○ Does not favor either the intervention or the comparison<br>○ Probably favors the intervention<br>○ Favors the intervention<br>○ Varies<br>● No included studies	Cost effective not included studies.	

Equity
What would be the impact on health equity?

JUDGEMENT	RESEARCH EVIDENCE	ADDITIONAL CONSIDERATIONS
○ Reduced<br>○ Probably reduced<br>○ Probably no impact<br>○ Probably increased<br>○ Increased<br>● Varies<br>○ Don't know	Could increase equity if more ECD kidneys are utilized, particularly in older patients. The organ pool could be expanded with more organs utilized.	

Acceptability
Is the intervention acceptable to key stakeholders?

JUDGEMENT	RESEARCH EVIDENCE	ADDITIONAL CONSIDERATIONS
○ No<br>○ Probably no<br>○ Probably yes<br>● Yes<br>○ Varies<br>○ Don't know	SCD and ECD kidneys currently being used.	

Feasibility
Is the intervention feasible to implement?

JUDGEMENT	RESEARCH EVIDENCE	ADDITIONAL CONSIDERATIONS
○ No<br>○ Probably no<br>○ Probably yes<br>● Yes<br>○ Varies<br>○ Don't know	Current standard of practice is to use SCD kidney and ECD kidneyss. Potentially could be using more ECD kidneys.	

SUMMARY OF JUDGEMENTS

	JUDGEMENT						
**PROBLEM**	No	Probably no	Probably yes	**Yes**		Varies	Don't know
**DESIRABLE EFFECTS**	Trivial	Small	Moderate	Large		**Varies**	Don't know
**UNDESIRABLE EFFECTS**	Large	Moderate	Small	Trivial		**Varies**	Don't know
**CERTAINTY OF EVIDENCE**	**Very low**	Low	Moderate	High			No included studies
**VALUES**	Important uncertainty or variability	Possibly important uncertainty or variability	**Probably no important uncertainty or variability**	No important uncertainty or variability			
**BALANCE OF EFFECTS**	**Favors the comparison**	Probably favors the comparison	Does not favor either the intervention or the comparison	Probably favors the intervention	Favors the intervention	Varies	Don't know
**RESOURCES REQUIRED**	Large costs	Moderate costs	Negligible costs and savings	Moderate savings	Large savings	Varies	**Don't know**
**CERTAINTY OF EVIDENCE OF REQUIRED RESOURCES**	Very low	Low	Moderate	High			**No included studies**
**COST EFFECTIVENESS**	Favors the comparison	Probably favors the comparison	Does not favor either the intervention or the comparison	Probably favors the intervention	Favors the intervention	Varies	**No included studies**
**EQUITY**	Reduced	Probably reduced	Probably no impact	Probably increased	Increased	**Varies**	Don't know
**ACCEPTABILITY**	No	Probably no	Probably yes	**Yes**		Varies	Don't know
**FEASIBILITY**	No	Probably no	Probably yes	**Yes**		Varies	Don't know

TYPE OF RECOMMENDATION

Strong recommendation against the intervention	Conditional recommendation against the intervention	Conditional recommendation for either the intervention or the comparison	Conditional recommendation for the intervention	Strong recommendation for the intervention
○	○	●	○	○

CONCLUSIONS

Recommendation

We suggest using SCD kidneys for transplant candidates(conditional recommendation, very low certainty of evidence)
We suggest using ECD kidneys in selected transplant candidates (conditional recommendation, very low certainty of evidence)

Justification

SCD kidneys were associated with improved survival compared to ECD kidneys. However, when comparing SCD and ECD kidneys in terms of acute rejection, readmission to hospital and graft survival the difference between SCD and ECD kidneys was trivial to small. Therefore, wider utilization of ECD kidneys can be considered in selected patients.

Subgroup considerations

None

Implementation considerations

ECD kidneys are already being utilized in practice. Implementaiton considerations include how to operationalize wider utilizations, shared decision making when using ECD kidneys, patient and provider preferences.

Monitoring and evaluation

Centers could monitor the characteristics of donors on an ongoing bases
Use the national datasystem to assess practice hetergeneity and follow outcomes of patients who use receive ECD.

Research priorities

Consider a provider preferences survey including conjoint analysis that will help determine what drives decision making for using ECD kidneys.
Further studies are needed on quality of life
Cost effectiveness studies.

APPENDICES

Appendix 1

Outcomes	№ of participants (studies) Follow-up	Certainty of the evidence (GRADE)	Relative effect (95% CI)	Anticipated absolute effects* (95% CI)	
				**Risk with SCD kidneys**	**Risk difference with Update ECD kidneys**
Mortality (Overall)	58453 (19 non-randomized studies)	⊕◯◯◯ Very low [a]	**RR 1.50** (1.25 to 1.80)	Study population	
				129 per 1,000	**65 more per 1,000** (32 more to 104 more)
Acute Rejection	15307 (35 non-randomized studies)	⊕◯◯◯ Very low [a,b,c]	**RR 1.10** (0.89 to 1.37)	Study population	
				171 per 1,000	**17 more per 1,000** (19 fewer to 63 more)
Delayed graft function	287190 (46 non-randomized studies)	⊕◯◯◯ Very low [a,b,c]	**RR 1.23** (1.04 to 1.46)	Study population	
				232 per 1,000	**53 more per 1,000** (9 more to 107 more)
Graft Survival (Overall)	238427 (39 non-randomized studies)	⊕◯◯◯ Very low [a,b,c]	**RR 0.97** (0.93 to 1.01)	Study population	
				799 per 1,000	**24 fewer per 1,000** (56 fewer to 8 more)
Death Censored Graft Survival	105051 (10 RCTs)	⊕⊕◯◯ Low [a,b]	**RR 0.95** (0.90 to 0.99)	Study population	
				864 per 1,000	**43 fewer per 1,000** (86 fewer to 9 fewer)

a. rated down for heterogenity
b. rated down for risk of bias as potential for double counting across studies using similar databases
c. rated down as confidence intervals include the line of no effect

Appendix 2

Outcomes	Anticipated absolute effects* (95% CI)		Relative effect (95% CI)	№ of participants (studies)	Certainty of the evidence (GRADE)	Comments
	Risk with SCD kidneys	Risk with Update ECD kidneys				
Mortality (Overall)	Study population		**RR 1.50** (1.25 to 1.80)	58453 (19 non-randomized studies)	⊕◯◯◯ Very low [a]	
	129 per 1,000	**194 per 1,000** (162 to 233)				
Acute Rejection	Study population		**RR 1.10** (0.89 to 1.37)	15307 (35 non-randomized studies)	⊕◯◯◯ Very low [a,b,c]	
	171 per 1,000	**188 per 1,000** (152 to 234)				
Delayed graft function	Study population		**RR 1.23** (1.04 to 1.46)	287190 (46 non-randomized studies)	⊕◯◯◯ Very low [a,b,c]	
	232 per 1,000	**285 per 1,000** (241 to 339)				
Graft Survival (Overall)	Study population		**RR 0.97** (0.93 to 1.01)	238427 (39 non-randomized studies)	⊕◯◯◯ Very low [a,b,c]	
	799 per 1,000	**775 per 1,000** (743 to 807)				
Death Censored Graft Survival	Study population		**RR 0.95** (0.90 to 0.99)	105051 (10 RCTs)	⊕⊕◯◯ Low [a,b]	
	864 per 1,000	**821 per 1,000** (778 to 855)				

a. rated down for heterogeneity
b. rated down for risk of bias as potential for double counting across studies using similar databases
c. rated down as confidence intervals include the line of no effect

QUESTION

Should older donors vs. younger donors be used for Renal transplant?	
**POPULATION:**	Renal transplant
**INTERVENTION:**	older donors
**COMPARISON:**	younger donors
**MAIN OUTCOMES:**	Mortality; Death Censored Graft Survival; Acute Rejection; Patient Survival; Graft Survival; Delayed Graft Function; Rejection;
**SETTING:**	
**PERSPECTIVE:**	
**BACKGROUND:**	
**CONFLICT OF INTERESTS:**	

ASSESSMENT

Problem
Is the problem a priority?

JUDGEMENT	RESEARCH EVIDENCE	ADDITIONAL CONSIDERATIONS
○ No<br>○ Probably no<br>○ Probably yes<br>● Yes<br>○ Varies<br>○ Don't know	*See Appendix 1*	

Desirable Effects
How substantial are the desirable anticipated effects?

JUDGEMENT	RESEARCH EVIDENCE	ADDITIONAL CONSIDERATIONS
○ Trivial<br>○ Small<br>○ Moderate<br>○ Large<br>● Varies<br>○ Don't know	*See Appendix 1*	Younger kidney donors have better survival (overall and death censored graft survival), and less acute rejection moderate effects.<br><br>With respect to older kidney donors compared to younger kidney donors, overall rejection is similar, death censored graft survival are small differences.

Undesirable Effects
How substantial are the undesirable anticipated effects?

JUDGEMENT	RESEARCH EVIDENCE	ADDITIONAL CONSIDERATIONS

○ Large<br>○ Moderate<br>○ Small<br>○ Trivial<br>● Varies<br>○ Don't know	*See Appendix 1*	As above.

Certainty of evidence

What is the overall certainty of the evidence of effects?

JUDGEMENT	RESEARCH EVIDENCE	ADDITIONAL CONSIDERATIONS
● Very low<br>○ Low<br>○ Moderate<br>○ High<br>○ No included studies	Concerns with registry studies, possible double counting of events at different time points. Differences in definitions applied. All are observational studies with potential biases.	

Values

Is there important uncertainty about or variability in how much people value the main outcomes?

JUDGEMENT	RESEARCH EVIDENCE	ADDITIONAL CONSIDERATIONS
○ Important uncertainty or variability<br>○ Possibly important uncertainty or variability<br>○ Probably no important uncertainty or variability<br>● No important uncertainty or variability		

Balance of effects

Does the balance between desirable and undesirable effects favor the intervention or the comparison?

JUDGEMENT	RESEARCH EVIDENCE	ADDITIONAL CONSIDERATIONS

● Favors the comparison<br>○ Probably favors the comparison<br>○ Does not favor either the intervention or the comparison<br>○ Probably favors the intervention<br>○ Favors the intervention<br><br>○ Varies<br>○ Don't know	Younger kidney donors had improved survival (patient and graft), and less rejection. However, older kidney donor organs and similar other outcomes compared to younger kidney donors.	

Resources required

How large are the resource requirements (costs)?

JUDGEMENT	RESEARCH EVIDENCE	ADDITIONAL CONSIDERATIONS
○ Large costs<br>● Moderate costs<br>○ Negligible costs and savings<br>○ Moderate savings<br>○ Large savings<br><br>○ Varies<br>○ Don't know	Potentially larger costs if using older donors with delayed graft function, or acute rejection.	

Certainty of evidence of required resources

What is the certainty of the evidence of resource requirements (costs)?

JUDGEMENT	RESEARCH EVIDENCE	ADDITIONAL CONSIDERATIONS
○ Very low<br>○ Low<br>○ Moderate<br>○ High<br><br>● No included studies		

Cost effectiveness

Does the cost-effectiveness of the intervention favor the intervention or the comparison?

JUDGEMENT	RESEARCH EVIDENCE	ADDITIONAL CONSIDERATIONS

○ Favors the comparison<br>○ Probably favors the comparison<br>○ Does not favor either the intervention or the comparison<br>○ Probably favors the intervention<br>○ Favors the intervention<br><br>○ Varies<br>● No included studies		

Equity
What would be the impact on health equity?

JUDGEMENT	RESEARCH EVIDENCE	ADDITIONAL CONSIDERATIONS
○ Reduced<br>○ Probably reduced<br>○ Probably no impact<br>○ Probably increased<br>○ Increased<br><br>● Varies<br>○ Don't know		

Acceptability
Is the intervention acceptable to key stakeholders?

JUDGEMENT	RESEARCH EVIDENCE	ADDITIONAL CONSIDERATIONS
○ No<br>○ Probably no<br>○ Probably yes<br>● Yes<br><br>○ Varies<br>○ Don't know	Already implementing both types of kidney donors in current practice.	

Feasibility
Is the intervention feasible to implement?

JUDGEMENT	RESEARCH EVIDENCE	ADDITIONAL CONSIDERATIONS
○ No<br>○ Probably no<br>○ Probably yes<br>● Yes<br><br>○ Varies<br>○ Don't know	Yes as already implemented in current practice.	

SUMMARY OF JUDGEMENTS

	JUDGEMENT						
**PROBLEM**	No	Probably no	Probably yes	**Yes**		Varies	Don't know
**DESIRABLE EFFECTS**	Trivial	Small	Moderate	Large		**Varies**	Don't know
**UNDESIRABLE EFFECTS**	Large	Moderate	Small	Trivial		**Varies**	Don't know
**CERTAINTY OF EVIDENCE**	**Very low**	Low	Moderate	High			No included studies
**VALUES**	Important uncertainty or variability	Possibly important uncertainty or variability	Probably no important uncertainty or variability	**No important uncertainty or variability**			
**BALANCE OF EFFECTS**	**Favors the comparison**	Probably favors the comparison	Does not favor either the intervention or the comparison	Probably favors the intervention	Favors the intervention	Varies	Don't know
**RESOURCES REQUIRED**	Large costs	**Moderate costs**	Negligible costs and savings	Moderate savings	Large savings	Varies	Don't know
**CERTAINTY OF EVIDENCE OF REQUIRED RESOURCES**	Very low	Low	Moderate	High			**No included studies**
**COST EFFECTIVENESS**	Favors the comparison	Probably favors the comparison	Does not favor either the intervention or the comparison	Probably favors the intervention	Favors the intervention	Varies	**No included studies**
**EQUITY**	Reduced	Probably reduced	Probably no impact	Probably increased	Increased	**Varies**	Don't know
**ACCEPTABILITY**	No	Probably no	Probably yes	**Yes**		Varies	Don't know
**FEASIBILITY**	No	Probably no	Probably yes	**Yes**		Varies	Don't know

TYPE OF RECOMMENDATION

Strong recommendation against the intervention	**Conditional recommendation against the intervention**	Conditional recommendation for either the intervention or the comparison	Conditional recommendation for the intervention	Strong recommendation for the intervention
○	●	○	○	○

CONCLUSIONS

Recommendation

We suggest to used organs from younger kidney donors when available for kidney transplantation (conditional recommendation, very low certainty of evidence).
We suggest to use organs from older kidney donors in selected transplant candidates (conditional recommendation, very low certainty of evidence).

Justification

Younger kidney donors show improved outcomes in terms of patient survival, acute rejection, and delay graft function compared to older kidney donors. However, there was trivial to small differences between older kidney donors and younger kidney donors in terms of death censored graft survival. Older kidney donors can have wider utilization.

Subgroup considerations

Implementation considerations

There may be differences in outcomes across extremes of age of the kidney donor (ex. differences in a older donor who is 65 compared to 75). Older kidney donor are already being utilized in practice. Implementation consideration include how to operationalize wider utilization, shared decision making

Monitoring and evaluation

Centres should monitor the characteristics of donors on an ongoing bases. Use of the national data systems to assess practice heterogeneity and follow outcomes of patients who receive kidneys from older donors.

Research priorities

Future studies should consider quality of life and cost effectiveness of using older kidney donors. Research on organ donors to improve organ quality should be done and include modifiable factors as well as potential biomarkers that may identify a better quality organ from and older donor.

APPENDICES

Appendix 1

Outcomes	Anticipated absolute effects* (95% CI)		Relative effect (95% CI)	№ of participants (studies)	Certainty of the evidence (GRADE)	Comments
	Risk with younger donors	Risk with older donors				
Mortality	Study population		**RR 1.53** (1.15 to 2.02)	52609 (11 non-randomized studies)	⊕◯◯◯ Very low	
	72 per 1,000	**109 per 1,000** (82 to 144)				
Death Censored Graft Survival	Study population		**RR 0.97** (0.94 to 1.00)	33807 (14 non-randomized studies)	⊕◯◯◯ Very low	
	876 per 1,000	**850 per 1,000** (824 to 876)				
Acute Rejection	Study population		**RR 1.18** (1.02 to 1.37)	10959 (16 non-randomized studies)	⊕◯◯◯ Very low	
	215 per 1,000	**253 per 1,000** (219 to 294)				
Patient Survival	Study population		**RR 0.95** (0.93 to 0.98)	96515 (23 non-randomized studies)	⊕◯◯◯ Very low	
	766 per 1,000	**727 per 1,000** (712 to 750)				
Graft Survival	Study population		**RR 0.88** (0.86 to 0.91)	150849 (35 non-randomized studies)	⊕◯◯◯ Very low	
	792 per 1,000	**697 per 1,000** (681 to 720)				
Delayed Graft Function	Study population		**RR 1.29** (1.12 to 1.48)	21150 (27 non-randomized studies)	⊕◯◯◯ Very low	
	235 per 1,000	**303 per 1,000** (263 to 347)				
Rejection	Study population		**RR 1.23** (0.96 to 1.57)	1180 (3 non-randomized studies)	⊕◯◯◯ Very low	
	242 per 1,000	**298 per 1,000** (233 to 381)				

QUESTION

Should a kidney with AKI vs. no AKI be used for Renal Transplant?	
POPULATION:	Renal Transplant
INTERVENTION:	a kidney with AKI
COMPARISON:	no AKI
MAIN OUTCOMES:	Mortality AKI vs nonAKI - Mortality 1 year; Mortality AKI vs nonAKI; Mortality AKI vs nonAKI - Mortality 3 years; Mortality AKI vs nonAKI - Mortality follow up to 5 years; Graft survival AKI vs nonAKI - 1 year Graft survival; Graft survival AKI vs nonAKI - 3-year Graft survival; Graft survival AKI vs nonAKI - 5-year Graft survival; Graft survival AKI vs nonAKI - 10-year Graft survival; Graft failure/loss AKI vs nonAKI - Graft failure- 1 year; Graft failure/loss AKI vs nonAKI - Graft failure- 3 years; Acute rejection rate (up to a year) AKI vs nonAKI; Hemodialysis posttransplant- AKI vs nonAKI;
SETTING:	
PERSPECTIVE:	
BACKGROUND:	
CONFLICT OF INTERESTS:	

ASSESSMENT

Problem
Is the problem a priority?

JUDGEMENT	RESEARCH EVIDENCE	ADDITIONAL CONSIDERATIONS
○ No<br>○ Probably no<br>○ Probably yes<br>● Yes<br><br>○ Varies<br>○ Don't know	*See Appendix 2*	Heterogeneity in practice. AKI in context donorship.<br>Variability in definition of AKI- research definition, versus clinical definition, making this a priority.<br>AKI in kidneys used for transplant, not aki in all potential donors. Decision point is already made.

Desirable Effects
How substantial are the desirable anticipated effects?

JUDGEMENT	RESEARCH EVIDENCE	ADDITIONAL CONSIDERATIONS
● Trivial<br>○ Small<br>○ Moderate<br>○ Large<br><br>○ Varies<br>○ Don't know	*See Appendix 2*	Relative effect is trivial.<br>Considerations for changes in practice over time and differences in country practices.

Undesirable Effects
How substantial are the undesirable anticipated effects?

JUDGEMENT	RESEARCH EVIDENCE	ADDITIONAL CONSIDERATIONS

○ Large<br>○ Moderate<br>○ Small<br>● Trivial<br>○ Varies<br>○ Don't know	*See Appendix 1*	Considerations that the donors are overall young in the newest studies. Interaction between AKI donor and patient characteristics need to be considered.

Certainty of evidence

What is the overall certainty of the evidence of effects?

JUDGEMENT	RESEARCH EVIDENCE	ADDITIONAL CONSIDERATIONS
● Very low<br>○ Low<br>○ Moderate<br>○ High<br>○ No included studies		

Values

Is there important uncertainty about or variability in how much people value the main outcomes?

JUDGEMENT	RESEARCH EVIDENCE	ADDITIONAL CONSIDERATIONS
○ Important uncertainty or variability<br>○ Possibly important uncertainty or variability<br>○ Probably no important uncertainty or variability<br>● No important uncertainty or variability		Objective outcomes- patients and clinicians would value both.

Balance of effects

Does the balance between desirable and undesirable effects favor the intervention or the comparison?

JUDGEMENT	RESEARCH EVIDENCE	ADDITIONAL CONSIDERATIONS

○ Favors the comparison<br>○ Probably favors the comparison<br>● Does not favor either the intervention or the comparison<br>○ Probably favors the intervention<br>○ Favors the intervention<br><br>○ Varies<br>○ Don't know	*See Appendix 3*	

Resources required
How large are the resource requirements (costs)?

JUDGEMENT	RESEARCH EVIDENCE	ADDITIONAL CONSIDERATIONS
○ Large costs<br>○ Moderate costs<br>○ Negligible costs and savings<br>○ Moderate savings<br>○ Large savings<br><br>○ Varies<br>● Don't know	No studies identified.	

Certainty of evidence of required resources
What is the certainty of the evidence of resource requirements (costs)?

JUDGEMENT	RESEARCH EVIDENCE	ADDITIONAL CONSIDERATIONS
○ Very low<br>○ Low<br>○ Moderate<br>○ High<br><br>● No included studies		

Cost effectiveness
Does the cost-effectiveness of the intervention favor the intervention or the comparison?

JUDGEMENT	RESEARCH EVIDENCE	ADDITIONAL CONSIDERATIONS

○ Favors the comparison<br>○ Probably favors the comparison<br>○ Does not favor either the intervention or the comparison<br>○ Probably favors the intervention<br>○ Favors the intervention<br><br>○ Varies<br>● No included studies		

Equity
What would be the impact on health equity?

JUDGEMENT	RESEARCH EVIDENCE	ADDITIONAL CONSIDERATIONS
○ Reduced<br>○ Probably reduced<br>○ Probably no impact<br>○ Probably increased<br>○ Increased<br><br>○ Varies<br>● Don't know		Older recipient who is not doing well on dialysis, but could receive a kidney with AKI, this may increase equity?

Acceptability
Is the intervention acceptable to key stakeholders?

JUDGEMENT	RESEARCH EVIDENCE	ADDITIONAL CONSIDERATIONS
○ No<br>○ Probably no<br>● Probably yes<br>○ Yes<br><br>○ Varies<br>○ Don't know	Overall very low certainty of evidence between the AKI and non-AKI kidneys. Both are used in current practice.	

Feasibility
Is the intervention feasible to implement?

JUDGEMENT	RESEARCH EVIDENCE	ADDITIONAL CONSIDERATIONS
○ No<br>○ Probably no<br>○ Probably yes<br>● Yes<br><br>○ Varies<br>○ Don't know		

SUMMARY OF JUDGEMENTS

	JUDGEMENT						
PROBLEM	No	Probably no	Probably yes	**Yes**		Varies	Don't know
DESIRABLE EFFECTS	**Trivial**	Small	Moderate	Large		Varies	Don't know
UNDESIRABLE EFFECTS	Large	Moderate	Small	**Trivial**		Varies	Don't know
CERTAINTY OF EVIDENCE	**Very low**	Low	Moderate	High			No included studies
VALUES	Important uncertainty or variability	Possibly important uncertainty or variability	Probably no important uncertainty or variability	**No important uncertainty or variability**			
BALANCE OF EFFECTS	Favors the comparison	Probably favors the comparison	**Does not favor either the intervention or the comparison**	Probably favors the intervention	Favors the intervention	Varies	Don't know
RESOURCES REQUIRED	Large costs	Moderate costs	Negligible costs and savings	Moderate savings	Large savings	Varies	**Don't know**
CERTAINTY OF EVIDENCE OF REQUIRED RESOURCES	Very low	Low	Moderate	High			**No included studies**
COST EFFECTIVENESS	Favors the comparison	Probably favors the comparison	Does not favor either the intervention or the comparison	Probably favors the intervention	Favors the intervention	Varies	**No included studies**
EQUITY	Reduced	Probably reduced	Probably no impact	Probably increased	Increased	Varies	**Don't know**
ACCEPTABILITY	No	Probably no	**Probably yes**	Yes		Varies	Don't know
FEASIBILITY	No	Probably no	Probably yes	**Yes**		Varies	Don't know

TYPE OF RECOMMENDATION

Strong recommendation against the intervention ○	Conditional recommendation against the intervention ○	**Conditional recommendation for either the intervention or the comparison ●**	Conditional recommendation for the intervention ○	Strong recommendation for the intervention ○

CONCLUSIONS

Recommendation

We suggest either a AKI or non-AKI kidney could be used based on clinician assessment and donor factors (conditional recommendation, very low certainty of evidence).

Justification

Subgroup considerations

Implementation considerations

Monitoring and evaluation

Research priorities

Future research should use standardized definition of AKI (ex KDIGO) to examine the differences between AKI and non-AKI kidneys with consistent donor selection.
Use of large multicenter and multinational studies.

APPENDICES

Appendix 1

Outcomes	№ of participants (studies) Follow-up	Certainty of the evidence (GRADE)	Relative effect (95% CI)	Anticipated absolute effects* (95% CI)	
				**Risk with no AKI**	**Risk difference with a kidney with AKI**
Mortality AKI vs nonAKI - Mortality 1 year	2364 (3 observational studies)	⨁◯◯◯ Very low [a,b]	**RR 0.75** (0.33 to 1.71)	Study population	
				63 per 1,000	**16 fewer per 1,000** (42 fewer to 44 more)
Mortality AKI vs nonAKI	4487 (11 observational studies)	⨁◯◯◯ Very low	**RR 0.80** (0.56 to 1.14)	Study population	
				61 per 1,000	**12 fewer per 1,000** (27 fewer to 8 more)
Mortality AKI vs nonAKI - Mortality 3 years	165 (1 observational study)	⨁◯◯◯ Very low	**RR 1.00** (0.36 to 2.78)	Study population	
				91 per 1,000	**0 fewer per 1,000** (58 fewer to 162 more)
Mortality AKI vs nonAKI - Mortality follow up to 5 years	569 (2 observational studies)	⨁◯◯◯ Very low [a,b,c]	**RR 0.79** (0.17 to 3.61)	Study population	
				48 per 1,000	**10 fewer per 1,000** (40 fewer to 126 more)
Graft survival AKI vs nonAKI - 1 year Graft survival	16072 (14 observational studies)	⨁◯◯◯ Very low [d,e]	**RR 1.03** (0.98 to 1.08)	Study population	
				886 per 1,000	**27 more per 1,000** (18 fewer to 71 more)
Graft survival AKI vs nonAKI - 3-year Graft survival	12118 (5 observational studies)	⨁◯◯◯ Very low [d,e]	**RR 1.03** (0.97 to 1.09)	Study population	
				854 per 1,000	**26 more per 1,000** (26 fewer to 77 more)
Graft survival AKI vs nonAKI - 5-year Graft survival	13409 (7 observational studies)	⨁◯◯◯ Very low [d,e]	**RR 0.98** (0.96 to 1.01)	Study population	
				775 per 1,000	**15 fewer per 1,000** (31 fewer to 8 more)
Graft survival AKI vs nonAKI - 10-year Graft survival	402 (2 observational studies)	⨁◯◯◯ Very low [e]	**RR 1.08** (0.95 to 1.23)	Study population	
				677 per 1,000	**54 more per 1,000** (34 fewer to 156 more)
Graft failure/loss AKI vs nonAKI - Graft failure- 1 year	17995 (12 observational studies)	⨁◯◯◯ Very low [e,f]	**RR 1.14** (1.02 to 1.27)	Study population	
				89 per 1,000	**12 more per 1,000** (2 more to 24 more)
Graft failure/loss AKI vs nonAKI - Graft failure- 3 years	2679 (3 observational studies)	⨁◯◯◯ Very low [b,e,f]	**RR 1.03** (0.84 to 1.26)	Study population	
				155 per 1,000	**5 more per 1,000** (25 fewer to 40 more)
Acute rejection rate (up to a year) AKI vs nonAKI	7334 (24 observational studies)	⨁◯◯◯ Very low [b,e,f]	**RR 1.02** (0.94 to 1.11)	Study population	
				247 per 1,000	**5 more per 1,000** (15 fewer to 27 more)

Hemodialysis posttransplant- AKI vs nonAKI	98<br>(1 observational study)	⨁◯◯◯<br>Very low [b,e]	**RR 1.53**<br>(0.88 to 2.68)	Study population	
				277 per 1,000	**147 more per 1,000**<br>(33 fewer to 465 more)

a. Rated down one level for heterogeneity
b. Rated down for few number of events, and wide confidence intervals that include the line of no effect
c. Could not formally assess due to <10 studies
d. Rated down as it includes the line of no effect
e. Rated down for possible double counting of events between studies
f. Rated down for inconsistency of effect between studies.

Appendix 2

Outcomes	Anticipated absolute effects* (95% CI)		Relative effect (95% CI)	№ of participants (studies)	Certainty of the evidence (GRADE)	Comments
	Risk with no AKI	Risk with a kidney with AKI				
Mortality AKI vs nonAKI - Mortality 1 year	Study population		**RR 0.75** (0.33 to 1.71)	2364 (3 observational studies)	⊕◯◯◯ Very low [a,b]	
	63 per 1,000	**47 per 1,000** (21 to 107)				
Mortality AKI vs nonAKI	Study population		**RR 0.80** (0.56 to 1.14)	4487 (11 observational studies)	⊕◯◯◯ Very low	
	61 per 1,000	**49 per 1,000** (34 to 69)				
Mortality AKI vs nonAKI - Mortality 3 years	Study population		**RR 1.00** (0.36 to 2.78)	165 (1 observational study)	⊕◯◯◯ Very low	
	91 per 1,000	**91 per 1,000** (33 to 253)				
Mortality AKI vs nonAKI - Mortality follow up to 5 years	Study population		**RR 0.79** (0.17 to 3.61)	569 (2 observational studies)	⊕◯◯◯ Very low [a,b,c]	
	48 per 1,000	**38 per 1,000** (8 to 174)				
Graft survival AKI vs nonAKI - 1 year Graft survival	Study population		**RR 1.03** (0.98 to 1.08)	16072 (14 observational studies)	⊕◯◯◯ Very low [d,e]	
	886 per 1,000	**912 per 1,000** (868 to 957)				
Graft survival AKI vs nonAKI - 3-year Graft survival	Study population		**RR 1.03** (0.97 to 1.09)	12118 (5 observational studies)	⊕◯◯◯ Very low [d,e]	
	854 per 1,000	**880 per 1,000** (829 to 931)				
Graft survival AKI vs nonAKI - 5-year Graft survival	Study population		**RR 0.98** (0.96 to 1.01)	13409 (7 observational studies)	⊕◯◯◯ Very low [d,e]	
	775 per 1,000	**759 per 1,000** (744 to 783)				
Graft survival AKI vs nonAKI - 10-year Graft survival	Study population		**RR 1.08** (0.95 to 1.23)	402 (2 observational studies)	⊕◯◯◯ Very low [e]	
	677 per 1,000	**731 per 1,000** (643 to 833)				
Graft failure/loss AKI vs nonAKI - Graft failure- 1 year	Study population		**RR 1.14** (1.02 to 1.27)	17995 (12 observational studies)	⊕◯◯◯ Very low [e,f]	
	89 per 1,000	**102 per 1,000** (91 to 113)				
Graft failure/loss AKI vs nonAKI - Graft failure- 3 years	Study population		**RR 1.03** (0.84 to 1.26)	2679 (3 observational studies)	⊕◯◯◯ Very low [b,e,f]	
	155 per 1,000	**160 per 1,000** (130 to 195)				
Acute rejection rate (up to a year) AKI vs nonAKI	Study population		**RR 1.02** (0.94 to 1.11)	7334 (24 observational studies)	⊕◯◯◯ Very low [b,e,f]	
	247 per 1,000	**252 per 1,000** (232 to 274)				
Hemodialysis posttransplant-AKI vs nonAKI	Study population		**RR 1.53** (0.88 to 2.68)	98 (1 observational study)	⊕◯◯◯ Very low [b,e]	

	277 per 1,000	**424 per 1,000**<br>(244 to 742)				

a. Rated down one level for heterogeneity
b. Rated down for few number of events, and wide confidence intervals that include the line of no effect
c. Could not formally assess due to <10 studies
d. Rated down as it includes the line of no effect
e. Rated down for possible double counting of events between studies
f. Rated down for inconsistency of effect between studies.

Appendix 3

Outcomes	Importance	Certainty of the evidence (GRADE)
Mortality AKI vs nonAKI - Mortality 1 year	CRITICAL	⨁◯◯◯ Very low [a,b]
Mortality AKI vs nonAKI	CRITICAL	⨁◯◯◯ Very low
Mortality AKI vs nonAKI - Mortality 3 years	CRITICAL	⨁◯◯◯ Very low
Mortality AKI vs nonAKI - Mortality follow up to 5 years	CRITICAL	⨁◯◯◯ Very low [a,b,c]
Graft survival AKI vs nonAKI - 1 year Graft survival	CRITICAL	⨁◯◯◯ Very low [d,e]
Graft survival AKI vs nonAKI - 3-year Graft survival	CRITICAL	⨁◯◯◯ Very low [d,e]
Graft survival AKI vs nonAKI - 5-year Graft survival	CRITICAL	⨁◯◯◯ Very low [d,e]
Graft survival AKI vs nonAKI - 10-year Graft survival	CRITICAL	⨁◯◯◯ Very low [e]
Graft failure/loss AKI vs nonAKI - Graft failure- 1 year	CRITICAL	⨁◯◯◯ Very low [e,f]
Graft failure/loss AKI vs nonAKI - Graft failure- 3 years	CRITICAL	⨁◯◯◯ Very low [b,e,f]
Acute rejection rate (up to a year) AKI vs nonAKI	IMPORTANT	⨁◯◯◯ Very low [b,e,f]
Hemodialysis posttransplant- AKI vs nonAKI	IMPORTANT	⨁◯◯◯ Very low [b,e]

a. Rated down one level for heterogeneity
b. Rated down for few number of events, and wide confidence intervals that include the line of no effect
c. Could not formally assess due to <10 studies
d. Rated down as it includes the line of no effect
e. Rated down for possible double counting of events between studies
f. Rated down for inconsistency of effect between studies.

QUESTION

Should Standard criteria kidney with AKI vs. Extended criteria kidney with AKI be used for Renal transplant?	
**POPULATION:**	Renal transplant
**INTERVENTION:**	Standard criteria kidney with AKI
**COMPARISON:**	Extended criteria kidney with AKI
**MAIN OUTCOMES:**	Mortality AKI-SCD vs AKI-ECD; Graft survival AKI-SCD vs AKI-ECD; Delayed graft function AKI-SCD vs AKI-ECD; Acute rejection rate AKI-SCD vs AKI-ECD; eGFR at one year AKI-SCD vs AKI-ECD; Infection rate AKI-SCD vs AKI-ECD;
**SETTING:**	
**PERSPECTIVE:**	
**BACKGROUND:**	
**CONFLICT OF INTERESTS:**	

ASSESSMENT

Problem

Is the problem a priority?

JUDGEMENT	RESEARCH EVIDENCE	ADDITIONAL CONSIDERATIONS
○ No<br>○ Probably no<br>○ Probably yes<br>● Yes<br>○ Varies<br>○ Don't know	see table below	

Outcomes	№ of participants (studies) Follow-up	Certainty of the evidence (GRADE)	Relative effect (95% CI)	Anticipated absolute effects* (95% CI)	
				Risk with Extended criteria kidney with AKI	Risk difference with Standard criteria kidney with AKI
Mortality AKI-SCD vs AKI-ECD	777 (3 observational studies)	⨁◯◯◯ Very low[a,b,c]	**RR 1.1** (0.6 to 2.0)	Study population	
				63 per 1,000	**6 more per 1,000** (25 fewer to 63 more)
Graft survival AKI-SCD vs AKI-ECD	626 (3 observational studies)	⨁◯◯◯ Very low[c,d]	**RR 1.1** (1.0 to 1.2)	Study population	
				769 per 1,000	**77 more per 1,000**

					(0 fewer to 154 more)
Delayed graft function AKI-SCD vs AKI-ECD	1091 (6 observational studies)	⨁◯◯◯ Very low[a,b,c,e]	**RR 0.99** (0.88 to 1.11)	Study population	
				411 per 1,000	**4 fewer per 1,000** (49 fewer to 45 more)
Acute rejection rate AKI-SCD vs AKI-ECD	810 (5 observational studies)	⨁◯◯◯ Very low[a,b,c]	**RR 1.19** (0.89 to 1.59)	Study population	
				246 per 1,000	**47 more per 1,000** (27 fewer to 145 more)
eGFR at one year AKI-SCD vs AKI-ECD	38 (1 observational study)	⨁◯◯◯ Very low[d]	-	The mean eGFR at one year AKI-SCD vs AKI-ECD was **0**	MD **9.5 higher** (0.98 higher to 18.02 higher)
Infection rate AKI-SCD vs AKI-ECD	162 (1 observational study)	⨁◯◯◯ Very low[d]	**RR 0.56** (0.23 to 1.38)	Study population	
				217 per 1,000	**96 fewer per 1,000** (167 fewer to 83 more)

a. Rated down as point estimates vary across studies
b. Rated down for imprecision as low number of events, and confidence intervals overlap.
c. Assessment of publication bias could not be done as less then 9 studies identified.
d. Rated down for few number of events
e. Observational studies with possible duplicate populations.

Desirable Effects

How substantial are the desirable anticipated effects?

JUDGEMENT	RESEARCH EVIDENCE	ADDITIONAL CONSIDERATIONS
● Trivial<br>○ Small<br>○ Moderate<br>○ Large<br>○ Varies	*See Appendix 1*	

○ Don't know		

Undesirable Effects

How substantial are the undesirable anticipated effects?

JUDGEMENT	RESEARCH EVIDENCE	ADDITIONAL CONSIDERATIONS
○ Large<br>○ Moderate<br>○ Small<br>● Trivial<br>○ Varies<br>○ Don't know	*See Appendix 1*	Graft survival favours AKI-SCD

Certainty of evidence

What is the overall certainty of the evidence of effects?

JUDGEMENT	RESEARCH EVIDENCE	ADDITIONAL CONSIDERATIONS
● Very low<br>○ Low<br>○ Moderate<br>○ High<br>○ No included studies		

Values

Is there important uncertainty about or variability in how much people value the main outcomes?

JUDGEMENT	RESEARCH EVIDENCE	ADDITIONAL CONSIDERATIONS
○ Important uncertainty or variability<br>○ Possibly important uncertainty or variability<br>● Probably no important uncertainty or variability<br>○ No important uncertainty or		

variability		

Balance of effects

Does the balance between desirable and undesirable effects favor the intervention or the comparison?

JUDGEMENT	RESEARCH EVIDENCE	ADDITIONAL CONSIDERATIONS
○ Favors the comparison<br>○ Probably favors the comparison<br>● Does not favor either the intervention or the comparison<br>○ Probably favors the intervention<br>○ Favors the intervention<br>○ Varies<br>○ Don't know	(see table below)	

Outcomes	Relative effect (95% CI)	Anticipated absolute effects[*] (95% CI)			Certainty of the evidence (GRADE)	What happens
		Without Standard criteria kidney with AKI	With Standard criteria kidney with AKI	Difference		
Mortality AKI-SCD vs AKI-ECD<br>№ of participants: 777<br>(3 observational studies)	**RR 1.1** (0.6 to 2.0)	Study population			⨁◯◯◯ Very low[a,b,c]	
		6.3%	**6.9%** (3.8 to 12.6)	**0.6% more** (2.5 fewer to 6.3 more)		
Graft survival AKI-SCD vs AKI-ECD<br>№ of participants: 626<br>(3 observational studies)	**RR 1.1** (1.0 to 1.2)	Study population			⨁◯◯◯ Very low[c,d]	
		76.9%	**84.6%** (76.9 to 92.3)	**7.7% more** (0 fewer to 15.4 more)		
Delayed graft function AKI-SCD vs AKI-ECD<br>№ of participants: 1091<br>(6 observational studies)	**RR 0.99** (0.88 to 1.11)	Study population			⨁◯◯◯ Very low[a,b,c,e]	
		41.1%	**40.7%** (36.1 to 45.6)	**0.4% fewer** (4.9 fewer to 4.5 more)		
Acute rejection rate AKI-SCD vs AKI-ECD<br>№ of participants: 810	**RR 1.19** (0.89 to 1.59)	Study population			⨁◯◯◯ Very low[a,b,c]	
		24.6%	**29.3%** (21.9 to 39.2)	**4.7% more** (2.7 fewer to 14.5 more)		

(5 observational studies)						
eGFR at one year AKI-SCD vs AKI-ECD<br>№ of participants: 38<br>(1 observational study)	-	The mean eGFR at one year AKI-SCD vs AKI-ECD without standard criteria kidney with AKI was **0**	-	MD **9.5 higher** (0.98 higher to 18.02 higher)	⊕◯◯◯<br>Very low[d]	
Infection rate AKI-SCD vs AKI-ECD<br>№ of participants: 162<br>(1 observational study)	**RR 0.56** (0.23 to 1.38)	Study population			⊕◯◯◯<br>Very low[d]	
		21.7%	**12.2%** (5 to 30)	**9.6% fewer** (16.7 fewer to 8.3 more)		

a. Rated down as point estimates vary across studies
b. Rated down for imprecision as low number of events, and confidence intervals overlap.
c. Assessment of publication bias could not be done as less then 9 studies identified.
d. Rated down for few number of events
e. Observational studies with possible duplicate populations.

Resources required

How large are the resource requirements (costs)?

JUDGEMENT	RESEARCH EVIDENCE	ADDITIONAL CONSIDERATIONS

○ Large costs<br>○ Moderate costs<br>● Negligible costs and savings<br>○ Moderate savings<br>○ Large savings<br>○ Varies<br>○ Don't know	No studies specifically looking at this comparison and resources required. However, similar costs between the two.	

Certainty of evidence of required resources

What is the certainty of the evidence of resource requirements (costs)?

JUDGEMENT	RESEARCH EVIDENCE	ADDITIONAL CONSIDERATIONS
○ Very low<br>○ Low<br>○ Moderate<br>○ High<br>● No included studies		

Cost effectiveness

Does the cost-effectiveness of the intervention favor the intervention or the comparison?

JUDGEMENT	RESEARCH EVIDENCE	ADDITIONAL CONSIDERATIONS
○ Favors the comparison<br>○ Probably favors the comparison<br>○ Does not favor either the intervention or the comparison<br>○ Probably favors the intervention<br>○ Favors the intervention<br>○ Varies<br>● No included studies		

Equity
What would be the impact on health equity?

JUDGEMENT	RESEARCH EVIDENCE	ADDITIONAL CONSIDERATIONS
○ Reduced<br>○ Probably reduced<br>○ Probably no impact<br>○ Probably increased<br>○ Increased<br>○ Varies<br>● Don't know		

Acceptability
Is the intervention acceptable to key stakeholders?

JUDGEMENT	RESEARCH EVIDENCE	ADDITIONAL CONSIDERATIONS
○ No<br>○ Probably no<br>○ Probably yes<br>○ Yes<br>● Varies<br>○ Don't know	Acceptability of AKI-SCD or AKI-ECD could be variable.	Reference survey study of provider preferences.

Feasibility
Is the intervention feasible to implement?

JUDGEMENT	RESEARCH EVIDENCE	ADDITIONAL CONSIDERATIONS
○ No<br>○ Probably no<br>○ Probably yes<br>● Yes<br>○ Varies<br>○ Don't know	It is feasible to transplant AKI ECD and AKI SCD kidneys. Considerations are needed for a national program. Also may require a biopsy of the kidney prior to transplant, and has logistical considerations.	

SUMMARY OF JUDGEMENTS

	JUDGEMENT						
**PROBLEM**	No	Probably no	Probably yes	**Yes**		Varies	Don't know
**DESIRABLE EFFECTS**	**Trivial**	Small	Moderate	Large		Varies	Don't know

	JUDGEMENT						
UNDESIRABLE EFFECTS	Large	Moderate	Small	**Trivial**		Varies	Don't know
CERTAINTY OF EVIDENCE	**Very low**	Low	Moderate	High			No included studies
VALUES	Important uncertainty or variability	Possibly important uncertainty or variability	**Probably no important uncertainty or variability**	No important uncertainty or variability			
BALANCE OF EFFECTS	Favors the comparison	Probably favors the comparison	**Does not favor either the intervention or the comparison**	Probably favors the intervention	Favors the intervention	Varies	Don't know
RESOURCES REQUIRED	Large costs	Moderate costs	**Negligible costs and savings**	Moderate savings	Large savings	Varies	Don't know
CERTAINTY OF EVIDENCE OF REQUIRED RESOURCES	Very low	Low	Moderate	High			**No included studies**
COST EFFECTIVENESS	Favors the comparison	Probably favors the comparison	Does not favor either the intervention or the comparison	Probably favors the intervention	Favors the intervention	Varies	**No included studies**
EQUITY	Reduced	Probably reduced	Probably no impact	Probably increased	Increased	Varies	**Don't know**
ACCEPTABILITY	No	Probably no	Probably yes	Yes		**Varies**	Don't know
FEASIBILITY	No	Probably no	Probably yes	**Yes**		Varies	Don't know

TYPE OF RECOMMENDATION

Strong recommendation against the intervention	Conditional recommendation against the intervention	**Conditional recommendation for either the intervention or the comparison**	Conditional recommendation for the intervention	Strong recommendation for the intervention
○	○	●	○	○

CONCLUSIONS

Recommendation

We suggest that a AKI-SCD or a AKI-ECD for renal transplant (weak recommendation, very low certainty of evidence).

Justification

Subgroup considerations

Implementation considerations

Monitoring and evaluation

Research priorities

APPENDICES

Appendix 1

Outcomes	Anticipated absolute effects* (95% CI)		Relative effect (95% CI)	№ of participants (studies)	Certainty of the evidence (GRADE)	Comments
	**Risk with Extended criteria kidney with AKI**	**Risk with Standard criteria kidney with AKI**				
Mortality AKI-SCD vs AKI-ECD	Study population		**RR 1.1** (0.6 to 2.0)	777 (3 observational studies)	⨁◯◯◯ Very low[a,b,c]	
	63 per 1,000	**69 per 1,000** (38 to 126)				
Graft survival AKI-SCD vs AKI-ECD	Study population		**RR 1.1** (1.0 to 1.2)	626 (3 observational studies)	⨁◯◯◯ Very low[c,d]	
	769 per 1,000	**846 per 1,000** (769 to 923)				
Delayed graft function AKI-SCD vs AKI-ECD	Study population		**RR 0.99** (0.88 to 1.11)	1091 (6 observational studies)	⨁◯◯◯ Very low[a,b,c,e]	
	411 per 1,000	**407 per 1,000** (361 to 456)				
Acute rejection rate AKI-SCD vs AKI-ECD	Study population		**RR 1.19** (0.89 to 1.59)	810 (5 observational studies)	⨁◯◯◯ Very low[a,b,c]	
	246 per 1,000	**293 per 1,000** (219 to 392)				
eGFR at one year AKI-SCD vs AKI-ECD	The mean eGFR at one year AKI-SCD vs AKI-ECD was **0**	MD **9.5 higher** (0.98 higher to 18.02 higher)	-	38 (1 observational study)	⨁◯◯◯ Very low[d]	
Infection rate AKI-SCD vs AKI-ECD	Study population		**RR 0.56** (0.23 to 1.38)	162 (1 observational study)	⨁◯◯◯ Very low[d]	
	217 per 1,000	**122 per 1,000** (50 to 300)				

a. Rated down as point estimates vary across studies
b. Rated down for imprecision as low number of events, and confidence intervals overlap.
c. Assessment of publication bias could not be done as less then 9 studies identified.
d. Rated down for few number of events
e. Observational studies with possible duplicate populations.

QUESTION

Should DCD donors vs. NDD donors be used for renal transplant?	
**POPULATION:**	renal transplant
**INTERVENTION:**	DCD donors
**COMPARISON:**	NDD donors
**MAIN OUTCOMES:**	Mortality; Graft Loss; Death Censored-Graft Loss; Acute Rejection; Delayed graft function;
**SETTING:**	
**PERSPECTIVE:**	
**BACKGROUND:**	
**CONFLICT OF INTERESTS:**	

ASSESSMENT

Problem
Is the problem a priority?

JUDGEMENT	RESEARCH EVIDENCE	ADDITIONAL CONSIDERATIONS
○ No<br>○ Probably no<br>○ Probably yes<br>● Yes<br>○ Varies<br>○ Don't know		

Desirable Effects
How substantial are the desirable anticipated effects?

JUDGEMENT	RESEARCH EVIDENCE	ADDITIONAL CONSIDERATIONS
○ Trivial<br>○ Small<br>○ Moderate<br>○ Large<br>● Varies<br>○ Don't know	*See Appendix 1*	With respect to NDD there was moderate desirable effects with less delayed graft function with NDD kidneys, and improved mortality with NDD kidneys.

		There was more delayed graft function in DCD kidneys compared to NDD kidneys (undesirable effects).<br><br>There was trivial effects on graft loss, acute rejection. between DCD kidneys and NDD kidneys.

Undesirable Effects

How substantial are the undesirable anticipated effects?

JUDGEMENT	RESEARCH EVIDENCE	ADDITIONAL CONSIDERATIONS
○ Large<br>○ Moderate<br>○ Small<br>● Trivial<br>○ Varies<br>○ Don't know		

Certainty of evidence

What is the overall certainty of the evidence of effects?

JUDGEMENT	RESEARCH EVIDENCE	ADDITIONAL CONSIDERATIONS
● Very low<br>○ Low<br>○ Moderate<br>○ High<br>○ No included studies	With database studies there is the potential for double counting of subjects, and overall low event rates.	

Values

Is there important uncertainty about or variability in how much people value the main outcomes?

JUDGEMENT	RESEARCH EVIDENCE	ADDITIONAL CONSIDERATIONS

○ Important uncertainty or variability<br>○ Possibly important uncertainty or variability<br>○ Probably no important uncertainty or variability<br>● No important uncertainty or variability		

Balance of effects
Does the balance between desirable and undesirable effects favor the intervention or the comparison?

JUDGEMENT	RESEARCH EVIDENCE	ADDITIONAL CONSIDERATIONS
● Favors the comparison<br>○ Probably favors the comparison<br>○ Does not favor either the intervention or the comparison<br>○ Probably favors the intervention<br>○ Favors the intervention<br>○ Varies<br>○ Don't know	The balance of effects favours the comparator, NDD kidneys, compared to DCD with respect to mortality and delayed graft function. However, trivial difference between NDD and DCD with respect to other outcomes.	

Resources required
How large are the resource requirements (costs)?

JUDGEMENT	RESEARCH EVIDENCE	ADDITIONAL CONSIDERATIONS
○ Large costs<br>● Moderate costs<br>○ Negligible costs and savings<br>○ Moderate savings<br>○ Large savings<br>○ Varies<br>○ Don't know	Anticipate moderate costs with the use of DCD kidneys as more delayed graft function. No studies included that assess the cost.	

Certainty of evidence of required resources
What is the certainty of the evidence of resource requirements (costs)?

JUDGEMENT	RESEARCH EVIDENCE	ADDITIONAL CONSIDERATIONS

○ Very low<br>○ Low<br>○ Moderate<br>○ High<br>● No included studies		

Cost effectiveness
Does the cost-effectiveness of the intervention favor the intervention or the comparison?

JUDGEMENT	RESEARCH EVIDENCE	ADDITIONAL CONSIDERATIONS
○ Favors the comparison<br>○ Probably favors the comparison<br>○ Does not favor either the intervention or the comparison<br>○ Probably favors the intervention<br>○ Favors the intervention<br>○ Varies<br>● No included studies	Anticipate moderate costs with the use of DCD kidneys as more delayed graft function. No studies included that assess the cost.	

Equity
What would be the impact on health equity?

JUDGEMENT	RESEARCH EVIDENCE	ADDITIONAL CONSIDERATIONS
○ Reduced<br>○ Probably reduced<br>○ Probably no impact<br>○ Probably increased<br>○ Increased<br>○ Varies<br>● Don't know		

Acceptability
Is the intervention acceptable to key stakeholders?

JUDGEMENT	RESEARCH EVIDENCE	ADDITIONAL CONSIDERATIONS
○ No<br>○ Probably no<br>○ Probably yes<br>● Yes<br>○ Varies<br>○ Don't know	Already using both types of kidneys in current practice.	

Feasibility

Is the intervention feasible to implement?

JUDGEMENT	RESEARCH EVIDENCE	ADDITIONAL CONSIDERATIONS
○ No<br>○ Probably no<br>○ Probably yes<br>● Yes<br>○ Varies<br>○ Don't know	Already using both types of kidneys in current practice.	

SUMMARY OF JUDGEMENTS

	JUDGEMENT						
PROBLEM	No	Probably no	Probably yes	**Yes**		Varies	Don't know
DESIRABLE EFFECTS	Trivial	Small	Moderate	Large		**Varies**	Don't know
UNDESIRABLE EFFECTS	Large	Moderate	Small	**Trivial**		Varies	Don't know
CERTAINTY OF EVIDENCE	**Very low**	Low	Moderate	High			No included studies
VALUES	Important uncertainty or variability	Possibly important uncertainty or variability	Probably no important uncertainty or variability	**No important uncertainty or variability**			
BALANCE OF EFFECTS	**Favors the comparison**	Probably favors the comparison	Does not favor either the intervention or the comparison	Probably favors the intervention	Favors the intervention	Varies	Don't know

	JUDGEMENT						
**RESOURCES REQUIRED**	Large costs	**Moderate costs**	Negligible costs and savings	Moderate savings	Large savings	Varies	Don't know
**CERTAINTY OF EVIDENCE OF REQUIRED RESOURCES**	Very low	Low	Moderate	High			**No included studies**
**COST EFFECTIVENESS**	Favors the comparison	Probably favors the comparison	Does not favor either the intervention or the comparison	Probably favors the intervention	Favors the intervention	Varies	**No included studies**
**EQUITY**	Reduced	Probably reduced	Probably no impact	Probably increased	Increased	Varies	**Don't know**
**ACCEPTABILITY**	No	Probably no	Probably yes	**Yes**		Varies	Don't know
**FEASIBILITY**	No	Probably no	Probably yes	**Yes**		Varies	Don't know

TYPE OF RECOMMENDATION

Strong recommendation against the intervention	Conditional recommendation against the intervention	Conditional recommendation for either the intervention or the comparison	Conditional recommendation for the intervention	Strong recommendation for the intervention
○	●	○	○	○

CONCLUSIONS

Recommendation

We suggest using NDD kidneys when available for transplantation (conditional recommendation, very low certainty of evidence)
We suggest using DCD kidneys when offered to most transplant candidates based on candidate characteristics (conditional recommendation, very low certainty of evidence)
and

Remarks:
NDD compared to DCD were different in terms of delayed graft function and all cause mortality, however, were similar across other outcomes.

Justification

NDD compared to DCD were different in terms of delayed graft function and all cause mortality, however, were similar across other outcomes.

Subgroup considerations

Considerations for older donors and functional ischemic time as there could be effect modifiers.

Implementation considerations

Already being utilized in current practice. No further considerations required.

Monitoring and evaluation

Centers should monitor the characteristics of donors on an ongoing bases as well as outcomes for organs not utilized.

Research priorities

Research priorities include the impact of functional ischemic time after discontinuation of life support. Research is required to examined the differences in DCD donors who have received medical assistance in dying (MAiD) compared to non-MAiD DCD donors.

APPENDICES

Appendix 1

Outcomes	Anticipated absolute effects* (95% CI)		Relative effect (95% CI)	№ of participants (studies)	Certainty of the evidence (GRADE)	Comments
	**Risk with NDD donors**	**Risk with DCD donors**				
Mortality	Study population		**RR 1.33** (1.15 to 1.54)	59538 (9 non-randomized studies)	⨁◯◯◯ Very low	
	75 per 1,000	**100 per 1,000** (87 to 116)				
Graft Loss	Study population		**RR 1.08** (1.00 to 1.17)	26605 (7 non-randomized studies)	⨁◯◯◯ Very low	
	211 per 1,000	**228 per 1,000** (211 to 246)				
Death Censored-Graft Loss	Study population		**RR 1.04** (0.92 to 1.17)	50855 (2 non-randomized studies)	⨁◯◯◯ Very low	
	90 per 1,000	**94 per 1,000** (83 to 106)				
Acute Rejection	Study population		**RR 1.62** (0.77 to 3.42)	36511 (7 non-randomized studies)	⨁◯◯◯ Very low	
	264 per 1,000	**428 per 1,000** (203 to 903)				
Delayed graft function	Study population		**RR 1.89** (1.80 to 1.99)	164985 (10 non-randomized studies)	⨁◯◯◯ Very low	
	216 per 1,000	**409 per 1,000** (390 to 431)				
