# Supplementary material for: Optimizing the Use of Deceased Donor Kidneys at Risk of Discard: A Clinical Practice Guideline
Source: Transpl Int. 2025 Jun 26;38:14596. doi: 10.3389/ti.2025.14596 (PMC12240869; doi:10.3389/ti.2025.14596)

# Supplement 1: Evidence Summaries

## 1. **Evidence Summary:** ECD compared to Waitlist for renal transplant in ESRD

| Certainty assessment | | | | | | | № of patients | | Effect | | Certainty | Importance |
|---|---|---|---|---|---|---|---|---|---|---|---|---|
| № of studies | Study design | Risk of bias | Inconsistency | Indirectness | Imprecision | Other considerations | ECD | Waitlist | Relative (95% CI) | Absolute (95% CI) | | |
| **Mortality** | | | | | | | | | | | | |
| 2 | non-randomized studies | serious[a] | serious[b] | not serious | not serious | none | 1785/7821 (22.8%) | 28346/109291 (25.9%) | **RR 0.88** (0.84 to 0.92) | **31 fewer per 1,000** (from 41 fewer to 21 fewer) | ⨁◯◯◯ Very low | CRITICAL |

**CI:** confidence interval; **RR:** risk ratio

**Explanations**

a. Rated down due to high risk of bias amongst studies.
b. Rated down for inconsistency amongst results across studies.

# Forest Plots: ECD Versus Waitlist

## Mortality

| Study or Subgroup | ECD Events | ECD Total | Waitlist Events | Waitlist Total | Weight | Risk Ratio M-H, Fixed, 95% CI |
|---|---|---|---|---|---|---|
| MartinNavarro 2009 | 6 | 31 | 24 | 164 | 0.2% | 1.32 [0.59, 2.97] |
| Merion 2005 | 1779 | 7790 | 28322 | 109127 | 99.8% | 0.88 [0.84, 0.92] |
| Total (95% CI) | | 7821 | | 109291 | 100.0% | 0.88 [0.84, 0.92] |
| Total events | 1785 | | 28346 | | | |

Heterogeneity: $Chi^2 = 0.97$, $df = 1$ ($P = 0.32$); $I^2 = 0\%$
Test for overall effect: $Z = 5.93$ ($P < 0.00001$)

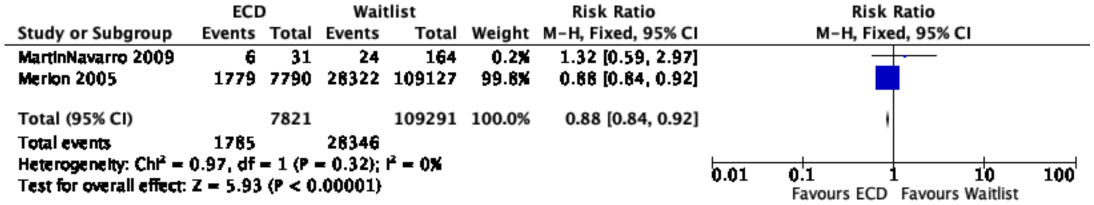

## 2. Evidence Summary: ECD Kidneys compared to Non-ECD (SCD) Kidneys

| Certainty assessment | | | | | | | № of patients | | Effect | | Certainty | Importance |
|---|---|---|---|---|---|---|---|---|---|---|---|---|
| № of studies | Study design | Risk of bias | Inconsistency | Indirectness | Imprecision | Other considerations | Update ECD kidneys | SCD kidneys | Relative (95% CI) | Absolute (95% CI) | | |
| **Mortality (Overall)** | | | | | | | | | | | | |
| 19 | non-randomized studies | not serious | serious[a] | not serious | not serious | none | 2206/10810 (20.4%) | 6165/47643 (12.9%) | **RR 1.50** (1.25 to 1.80) | **65 more per 1,000** (from 32 more to 104 more) | ⨁◯◯◯ Very low | CRITICAL |
| **Acute Rejection** | | | | | | | | | | | | |
| 35 | non-randomized studies | serious[b] | serious[a] | not serious | serious[c] | none | 905/5141 (17.6%) | 1740/10166 (17.1%) | **RR 1.10** (0.89 to 1.37) | **17 more per 1,000** (from 19 fewer to 63 more) | ⨁◯◯◯ Very low | CRITICAL |
| **Delayed graft function** | | | | | | | | | | | | |
| 46 | non-randomized studies | serious[b] | serious[a] | not serious | serious[c] | none | 14125/55475 (25.5%) | 53728/231715 (23.2%) | **RR 1.23** (1.04 to 1.46) | **53 more per 1,000** (from 9 more to 107 more) | ⨁◯◯◯ Very low | CRITICAL |
| **Graft Survival (Overall)** | | | | | | | | | | | | |
| 39 | non-randomized studies | serious[b] | serious[a] | not serious | serious[c] | none | 29654/42989 (69.0%) | 156191/195438 (79.9%) | **RR 0.97** (0.93 to 1.01) | **24 fewer per 1,000** (from 56 fewer to 8 more) | ⨁◯◯◯ Very low | CRITICAL |
| **Death Censored Graft Survival** | | | | | | | | | | | | |
| 10 | randomized trials | serious[b] | serious[a] | not serious | not serious | none | 14742/18441 (79.9%) | 74832/86610 (86.4%) | **RR 0.95** (0.90 to 0.99) | **43 fewer per 1,000** (from 86 fewer to 9 fewer) | ⨁⨁◯◯ Low | IMPORTANT |

**CI:** confidence interval; **RR:** risk ratio

**Explanations**

a. rated down for heterogeneity
b. rated down for risk of bias as potential for double counting across studies using similar databases
c. rated down as confidence intervals include the line of no effect

# Forest Plots: ECD Versus Non ECD

## Mortality

| Study or Subgroup | ECD Events Events | ECD Events Total | Non ECD (SCD) Events Events | Non ECD (SCD) Events Total | Weight | Risk Ratio M-H, Random, 95% CI |
|---|---|---|---|---|---|---|
| Al-Shraideh 2014 | 94 | 356 | 93 | 528 | 11.1% | 1.50 [1.16, 1.93] |
| Aubert 2015 | 135 | 916 | 128 | 1847 | 11.6% | 2.13 [1.69, 2.67] |
| Barba 2013 | 16 | 163 | 16 | 244 | 4.8% | 1.50 [0.77, 2.91] |
| Bell 2019 | 2 | 237 | 1 | 668 | 0.5% | 5.64 [0.51, 61.88] |
| Domagala 2009 | 12 | 14 | 83 | 86 | 11.8% | 0.89 [0.71, 1.10] |
| Ferreira 2017 | 18 | 150 | 42 | 467 | 6.5% | 1.33 [0.79, 2.25] |
| Golpani 2010 | 12 | 43 | 24 | 115 | 5.5% | 1.34 [0.74, 2.43] |
| Jung 2016 | 1 | 11 | 0 | 16 | 0.3% | 4.25 [0.19, 95.68] |
| Keitel 2004 | 20 | 148 | 35 | 434 | 6.5% | 1.68 [1.00, 2.81] |
| Khan 2006 | 9 | 55 | 0 | 55 | 0.4% | 19.00 [1.13, 318.63] |
| Kim 2014 | 1 | 14 | 4 | 31 | 0.7% | 0.55 [0.07, 4.51] |
| Klein 2013 | 23 | 253 | 94 | 1264 | 7.8% | 1.22 [0.79, 1.89] |
| Lee 2020 | 3 | 80 | 4 | 124 | 1.4% | 1.16 [0.27, 5.06] |
| Moore 2007 | 4 | 61 | 6 | 53 | 1.9% | 0.58 [0.17, 1.94] |
| Ojo 2005 | 1779 | 7790 | 5585 | 41052 | 14.1% | 1.68 [1.60, 1.76] |
| Rigotti 2014 | 42 | 231 | 17 | 231 | 6.3% | 2.47 [1.45, 4.21] |
| Singh 2011 | 31 | 222 | 29 | 356 | 7.1% | 1.71 [1.06, 2.76] |
| Whiting 1998 | 1 | 38 | 2 | 40 | 0.6% | 0.53 [0.05, 5.57] |
| Wiwattanathum 2016 | 3 | 28 | 2 | 32 | 1.0% | 1.71 [0.31, 9.53] |
| Total (95% CI) | | 10810 | | 47643 | 100.0% | 1.50 [1.25, 1.80] |
| Total events | 2206 | | 6165 | | | |

Heterogeneity: Tau² = 0.06; Chi² = 52.37, df = 18 (P < 0.0001); I² = 66%
Test for overall effect: Z = 4.43 (P < 0.00001)

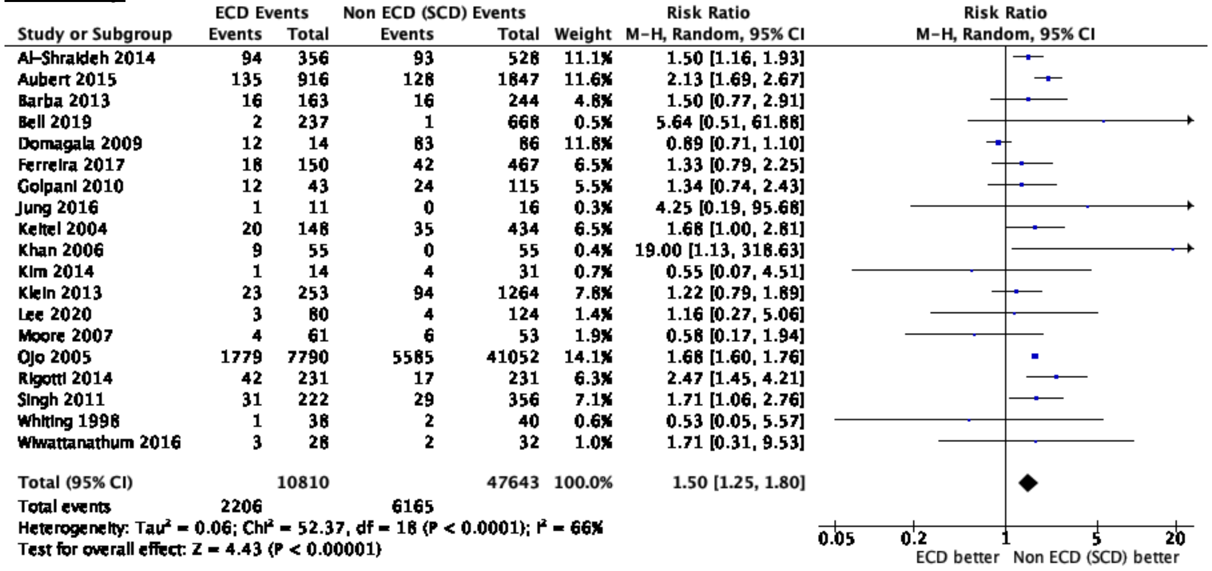

## Graft Loss

| Study or Subgroup | ECD Better Events | ECD Better Total | Non ECD (SCD) Better Events | Non ECD (SCD) Better Total | Weight | Risk Ratio M-H, Random, 95% CI |
|---|---|---|---|---|---|---|
| **1.12.1 Overall** | | | | | | |
| Barba 2013 | 47 | 163 | 43 | 244 | 34.3% | 1.64 [1.14, 2.35] |
| Bonsignore 2019 | 6 | 73 | 2 | 42 | 1.9% | 1.73 [0.36, 8.17] |
| Wiwattanathum 2016 | 3 | 28 | 0 | 32 | 0.5% | 7.97 [0.43, 147.82] |
| Subtotal (95% CI) | | 264 | | 318 | 36.7% | 1.68 [1.18, 2.38] |
| Total events | 56 | | 45 | | | |
| Heterogeneity: $\text{Tau}^2 = 0.00$; $\text{Chi}^2 = 1.13$, df = 2 ($P = 0.57$); $I^2 = 0\%$ | | | | | | |
| Test for overall effect: $Z = 2.89$ ($P = 0.004$) | | | | | | |
| **1.12.2 at 1 year** | | | | | | |
| Farney 2008 | 23 | 138 | 18 | 178 | 13.7% | 1.65 [0.93, 2.93] |
| Lee 2020 | 2 | 80 | 4 | 124 | 1.6% | 0.78 [0.15, 4.13] |
| Nakamura 2018 | 2 | 10 | 3 | 18 | 1.7% | 1.20 [0.24, 6.02] |
| Subtotal (95% CI) | | 228 | | 320 | 17.0% | 1.49 [0.89, 2.49] |
| Total events | 27 | | 25 | | | |
| Heterogeneity: $\text{Tau}^2 = 0.00$; $\text{Chi}^2 = 0.77$, df = 2 ($P = 0.68$); $I^2 = 0\%$ | | | | | | |
| Test for overall effect: $Z = 1.50$ ($P = 0.13$) | | | | | | |
| **1.12.3 at 3 years** | | | | | | |
| Subtotal (95% CI) | | 0 | | 0 | | Not estimable |
| Total events | 0 | | 0 | | | |
| Heterogeneity: Not applicable | | | | | | |
| Test for overall effect: Not applicable | | | | | | |
| **1.12.4 at 5 years** | | | | | | |
| Singh 2011 | 62 | 222 | 60 | 356 | 46.2% | 1.66 [1.21, 2.27] |
| Subtotal (95% CI) | | 222 | | 356 | 46.2% | 1.66 [1.21, 2.27] |
| Total events | 62 | | 60 | | | |
| Heterogeneity: Not applicable | | | | | | |
| Test for overall effect: $Z = 3.16$ ($P = 0.002$) | | | | | | |
| **Total (95% CI)** | | 714 | | 994 | 100.0% | 1.63 [1.32, 2.02] |
| Total events | 145 | | 130 | | | |
| Heterogeneity: $\text{Tau}^2 = 0.00$; $\text{Chi}^2 = 2.05$, df = 6 ($P = 0.92$); $I^2 = 0\%$ | | | | | | |
| Test for overall effect: $Z = 4.53$ ($P < 0.00001$) | | | | | | |
| Test for subgroup differences: $\text{Chi}^2 = 0.16$, df = 2 ($P = 0.92$), $I^2 = 0\%$ | | | | | | |

Risk Ratio
M-H, Random, 95% CI

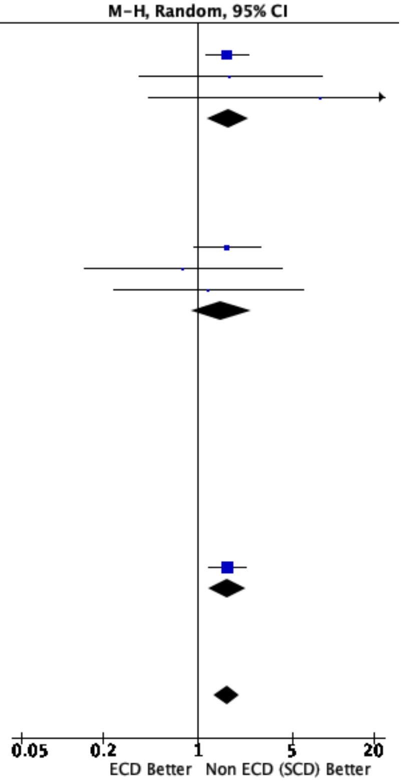

## Delayed Graft Function

| Study or Subgroup | ECD Better Events | ECD Better Total | Non ECD (SCD) Better Events | Non ECD (SCD) Better Total | Weight | Risk Ratio M-H, Random, 95% CI |
|---|---|---|---|---|---|---|
| Al-Shraideh 2014 | 91 | 356 | 185 | 528 | 2.6% | 0.73 [0.59, 0.90] |
| Aubert 2015 | 297 | 916 | 410 | 1847 | 2.7% | 1.46 [1.29, 1.66] |
| Balaz 2013 | 69 | 143 | 57 | 201 | 2.6% | 1.70 [1.29, 2.25] |
| Barba 2013 | 40 | 163 | 26 | 244 | 2.3% | 2.30 [1.47, 3.62] |
| Bell 2019 | 69 | 237 | 108 | 668 | 2.6% | 1.80 [1.38, 2.34] |
| Bonsignore 2019 | 41 | 73 | 10 | 42 | 2.1% | 2.36 [1.32, 4.20] |
| Cardinal 2019 | 23 | 41 | 26 | 81 | 2.3% | 1.75 [1.15, 2.65] |
| Domagala 2009 | 5 | 14 | 4 | 19 | 1.2% | 1.70 [0.55, 5.19] |
| Farney 2008 | 26 | 138 | 34 | 178 | 2.3% | 0.99 [0.62, 1.56] |
| Farney 2011 | 4 | 19 | 69 | 115 | 1.6% | 0.35 [0.15, 0.85] |
| Ferreira 2017 | 18 | 150 | 42 | 467 | 2.2% | 1.33 [0.79, 2.25] |
| Ferrer 2009 | 35 | 100 | 57 | 309 | 2.4% | 1.90 [1.33, 2.71] |
| Frutos 2006 | 46 | 77 | 141 | 265 | 2.6% | 1.12 [0.91, 1.39] |
| Golpani 2010 | 18 | 43 | 31 | 115 | 2.3% | 1.55 [0.98, 2.47] |
| Han 2014 | 7 | 40 | 26 | 259 | 1.7% | 1.74 [0.81, 3.75] |
| Heilman 2014 | 54 | 160 | 219 | 611 | 2.6% | 0.94 [0.74, 1.20] |
| Helantera 2020 | 4496 | 26066 | 21570 | 77979 | 2.7% | 0.62 [0.61, 0.64] |
| Hwang 2014 | 10 | 31 | 21 | 164 | 1.9% | 2.52 [1.32, 4.82] |
| Jacobi 2017 | 49 | 174 | 51 | 208 | 2.5% | 1.15 [0.82, 1.61] |
| Jung 2016 | 4 | 11 | 1 | 16 | 0.5% | 5.82 [0.75, 45.31] |
| Keitel 2004 | 9 | 148 | 219 | 434 | 2.0% | 0.12 [0.06, 0.23] |
| Khan 2006 | 33 | 55 | 49 | 55 | 2.6% | 0.67 [0.53, 0.85] |
| Kim 2013 | 5 | 26 | 9 | 117 | 1.4% | 2.50 [0.91, 6.85] |
| Kim 2014 | 3 | 14 | 2 | 31 | 0.7% | 3.32 [0.62, 17.72] |
| Klein 2013 | 159 | 253 | 711 | 1265 | 2.7% | 1.12 [1.01, 1.24] |
| Ko 2018 | 9 | 53 | 21 | 149 | 1.8% | 1.20 [0.59, 2.46] |
| Kumar 2006 | 33 | 55 | 33 | 55 | 2.5% | 1.00 [0.74, 1.36] |
| Lee 2020 | 27 | 80 | 33 | 124 | 2.3% | 1.27 [0.83, 1.94] |
| Lionaki 2014 | 87 | 140 | 96 | 187 | 2.7% | 1.21 [1.00, 1.46] |
| Locke 2007 | 3843 | 12812 | 12246 | 62800 | 2.7% | 1.54 [1.49, 1.59] |
| Mohan 2012 | 3880 | 11087 | 16528 | 79846 | 2.7% | 1.69 [1.64, 1.74] |
| Moore 2007 | 14 | 61 | 13 | 53 | 1.9% | 0.94 [0.48, 1.81] |
| Nakamura 2018 | 2 | 10 | 3 | 18 | 0.8% | 1.20 [0.24, 6.02] |
| Papachristou 2014 | 35 | 53 | 57 | 162 | 2.5% | 1.88 [1.41, 2.49] |
| Saidi 2007 | 25 | 163 | 24 | 163 | 2.2% | 1.04 [0.62, 1.75] |
| Serur 2013 | 105 | 250 | 142 | 427 | 2.6% | 1.26 [1.04, 1.54] |
| Serwanska-Swietek 2018 | 29 | 152 | 114 | 316 | 2.4% | 0.53 [0.37, 0.76] |
| Small 2013 | 77 | 243 | 59 | 280 | 2.5% | 1.50 [1.12, 2.01] |
| Song 2018 | 11 | 31 | 42 | 192 | 2.1% | 1.62 [0.94, 2.80] |
| Stratta 2016 | 211 | 571 | 95 | 351 | 2.6% | 1.37 [1.12, 1.67] |
| Sundberg 2004 | 4 | 37 | 11 | 53 | 1.3% | 0.52 [0.18, 1.51] |
| Swami 2012 | 8 | 19 | 4 | 25 | 1.3% | 2.63 [0.93, 7.46] |
| Tomita 2017 | 47 | 50 | 42 | 47 | 2.7% | 1.05 [0.93, 1.19] |
| Wiwattanathum 2016 | 21 | 28 | 10 | 32 | 2.1% | 2.40 [1.38, 4.19] |
| Yap 2021 | 30 | 41 | 52 | 94 | 2.6% | 1.32 [1.02, 1.71] |
| Zadori 2015 | 16 | 91 | 25 | 123 | 2.1% | 0.87 [0.49, 1.52] |
| Total (95% CI) | | 55475 | | 231715 | 100.0% | 1.23 [1.04, 1.46] |
| Total events | 14125 | | 53728 | | | |

Heterogeneity: $Tau^2 = 0.27$; $Chi^2 = 3110.20$, df = 45 ($P < 0.00001$); $I^2 = 99\%$

Test for overall effect: Z = 2.45 (P = 0.01)

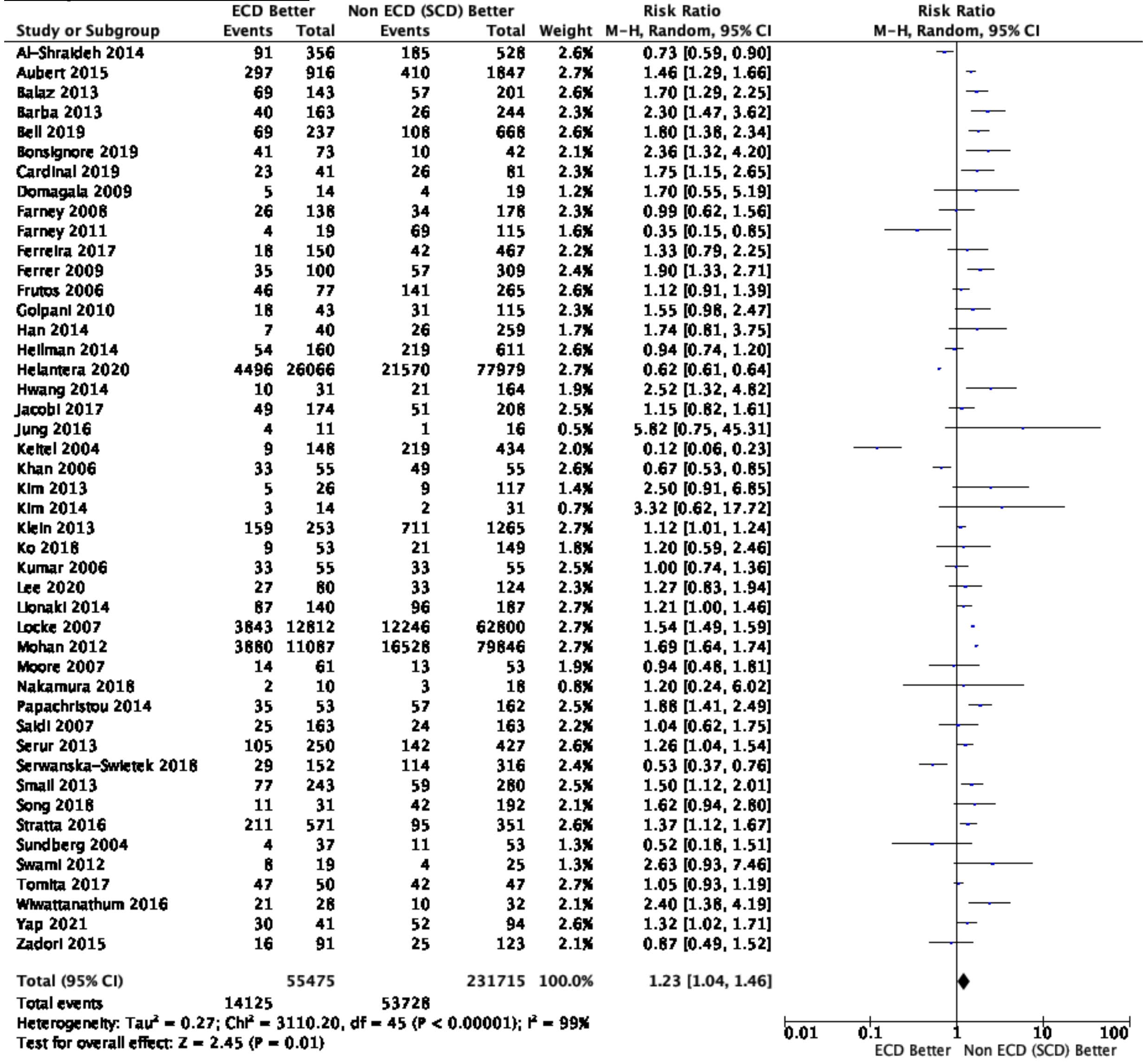

<u>Death-Censored Graft Survival</u>

| Study or Subgroup | ECD Better Events | ECD Better Total | Non ECD (SCD) Better Events | Non ECD (SCD) Better Total | Weight | Risk Ratio M-H, Random, 95% CI |
|---|---|---|---|---|---|---|
| **1.10.1 at 1 year** | | | | | | |
| Cabello 2010 | 158 | 180 | 173 | 180 | 7.9% | 0.91 [0.86, 0.97] |
| Farney 2011 | 17 | 19 | 108 | 115 | 4.5% | 0.95 [0.81, 1.12] |
| Merion 2005 | 6777 | 7790 | 38589 | 41052 | 9.0% | 0.93 [0.92, 0.93] |
| Rigotti 2014 | 222 | 231 | 408 | 416 | 8.7% | 0.98 [0.95, 1.01] |
| Small 2013 | 208 | 243 | 247 | 280 | 7.7% | 0.97 [0.91, 1.04] |
| Song 2018 | 575 | 0 | 0 | 0 | | Not estimable |
| Subtotal (95% CI) | | 8463 | | 42043 | 37.8% | 0.95 [0.91, 0.98] |
| Total events | 7957 | | 39525 | | | |
| Heterogeneity: Tau² = 0.00; Chi² = 15.91, df = 4 (P = 0.003); I² = 75% | | | | | | |
| Test for overall effect: Z = 2.93 (P = 0.003) | | | | | | |
| **1.10.2 at 3 years** | | | | | | |
| Rigotti 2014 | 219 | 231 | 395 | 416 | 8.6% | 1.00 [0.96, 1.04] |
| Subtotal (95% CI) | | 231 | | 416 | 8.6% | 1.00 [0.96, 1.04] |
| Total events | 219 | | 395 | | | |
| Heterogeneity: Not applicable | | | | | | |
| Test for overall effect: Z = 0.08 (P = 0.94) | | | | | | |
| **1.10.3 at 5 years** | | | | | | |
| Al-Shraideh 2014 | 260 | 356 | 429 | 528 | 7.4% | 0.90 [0.83, 0.97] |
| Merion 2005 | 5141 | 7790 | 32431 | 41052 | 8.9% | 0.84 [0.82, 0.85] |
| Rigotti 2014 | 213 | 231 | 379 | 416 | 8.3% | 1.01 [0.96, 1.06] |
| Small 2013 | 159 | 243 | 212 | 280 | 6.0% | 0.86 [0.77, 0.97] |
| Subtotal (95% CI) | | 8620 | | 42276 | 30.6% | 0.90 [0.80, 1.01] |
| Total events | 5773 | | 33451 | | | |
| Heterogeneity: Tau² = 0.01; Chi² = 60.26, df = 3 (P < 0.00001); I² = 95% | | | | | | |
| Test for overall effect: Z = 1.79 (P = 0.07) | | | | | | |
| **1.10.4 Overall** | | | | | | |
| Antonieta-Azancot 2013 | 104 | 117 | 40 | 44 | 6.0% | 0.98 [0.87, 1.10] |
| Klein 2013 | 212 | 253 | 1129 | 1265 | 8.0% | 0.94 [0.89, 0.99] |
| Small 2013 | 61 | 243 | 108 | 280 | 2.4% | 0.65 [0.50, 0.85] |
| Stratta 2016 | 416 | 514 | 184 | 286 | 6.6% | 1.26 [1.14, 1.38] |
| Subtotal (95% CI) | | 1127 | | 1875 | 23.0% | 0.96 [0.80, 1.16] |
| Total events | 793 | | 1461 | | | |
| Heterogeneity: Tau² = 0.03; Chi² = 37.07, df = 3 (P < 0.00001); I² = 92% | | | | | | |
| Test for overall effect: Z = 0.43 (P = 0.67) | | | | | | |
| **Total (95% CI)** | | 18441 | | 86610 | 100.0% | 0.95 [0.90, 0.99] |
| Total events | 14742 | | 74832 | | | |
| Heterogeneity: Tau² = 0.01; Chi² = 290.06, df = 13 (P < 0.00001); I² = 96% | | | | | | |
| Test for overall effect: Z = 2.21 (P = 0.03) | | | | | | |
| Test for subgroup differences: Chi² = 5.56, df = 3 (P = 0.14), I² = 46.0% | | | | | | |

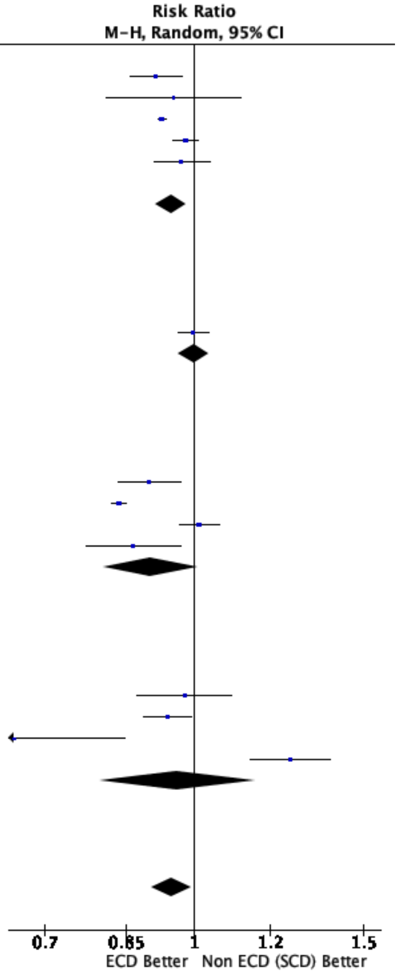

## Acute Rejection

| Study or Subgroup | ECD Better Events | ECD Better Total | Non ECD (SCD) Better Events | Non ECD (SCD) Better Total | Weight | Risk Ratio M-H, Random, 95% CI |
|---|---|---|---|---|---|---|
| Aubert 2015 | 142 | 916 | 128 | 1847 | 4.4% | 2.24 [1.79, 2.80] |
| Barba 2013 | 53 | 163 | 92 | 244 | 4.3% | 0.86 [0.66, 1.13] |
| Bell 2019 | 17 | 237 | 1 | 668 | 0.9% | 47.92 [6.41, 358.09] |
| Bonsignore 2019 | 9 | 73 | 2 | 42 | 1.5% | 2.59 [0.59, 11.42] |
| Dedinska 2019 | 18 | 50 | 20 | 107 | 3.6% | 1.93 [1.12, 3.31] |
| Farney 2008 | 17 | 138 | 15 | 178 | 3.3% | 1.46 [0.76, 2.82] |
| Farney 2011 | 3 | 19 | 22 | 115 | 2.1% | 0.83 [0.27, 2.49] |
| Ferreira 2017 | 1 | 150 | 10 | 467 | 0.9% | 0.31 [0.04, 2.41] |
| Ferrer 2009 | 34 | 100 | 49 | 309 | 4.1% | 2.14 [1.47, 3.12] |
| Gambino 2006 | 6 | 32 | 16 | 94 | 2.7% | 1.10 [0.47, 2.57] |
| Golpani 2010 | 10 | 43 | 24 | 115 | 3.3% | 1.11 [0.58, 2.13] |
| Hamed 2015 | 2 | 288 | 0 | 465 | 0.5% | 8.06 [0.39, 167.34] |
| Heilman 2014 | 21 | 160 | 113 | 611 | 3.9% | 0.71 [0.46, 1.09] |
| Jacobi 2017 | 39 | 144 | 40 | 181 | 4.1% | 1.23 [0.84, 1.80] |
| Jung 2016 | 3 | 11 | 1 | 16 | 0.8% | 4.36 [0.52, 36.68] |
| Keitel 2004 | 98 | 148 | 219 | 434 | 4.6% | 1.31 [1.13, 1.52] |
| Khan 2006 | 7 | 55 | 7 | 55 | 2.4% | 1.00 [0.38, 2.66] |
| Kim 2014 | 7 | 26 | 21 | 117 | 3.0% | 1.50 [0.71, 3.15] |
| Klein 2013 | 74 | 253 | 411 | 1265 | 4.5% | 0.90 [0.73, 1.11] |
| Ko 2018 | 17 | 53 | 58 | 149 | 3.9% | 0.82 [0.53, 1.28] |
| Kumar 2006 | 7 | 55 | 7 | 55 | 2.4% | 1.00 [0.38, 2.66] |
| Lee 2020 | 31 | 80 | 40 | 124 | 4.1% | 1.20 [0.83, 1.75] |
| Lionaki 2014 | 7 | 140 | 2 | 187 | 1.4% | 4.67 [0.99, 22.16] |
| Mezrich 2012 | 78 | 358 | 44 | 358 | 4.2% | 1.77 [1.26, 2.49] |
| Moore 2007 | 7 | 61 | 7 | 53 | 2.4% | 0.87 [0.33, 2.32] |
| Nakamura 2018 | 1 | 10 | 6 | 18 | 1.0% | 0.30 [0.04, 2.15] |
| Nardo 2011 | 15 | 152 | 47 | 182 | 3.6% | 0.38 [0.22, 0.66] |
| Palkoci 2018 | 9 | 50 | 22 | 107 | 3.1% | 0.88 [0.44, 1.76] |
| Papachristou 2014 | 3 | 53 | 13 | 162 | 1.9% | 0.71 [0.21, 2.38] |
| Serur 2013 | 28 | 250 | 73 | 427 | 4.0% | 0.66 [0.44, 0.98] |
| Serwanska-Swietek 2018 | 24 | 152 | 25 | 316 | 3.7% | 2.00 [1.18, 3.38] |
| Song 2018 | 3 | 31 | 24 | 192 | 2.0% | 0.77 [0.25, 2.42] |
| Stratta 2016 | 108 | 571 | 149 | 351 | 4.5% | 0.45 [0.36, 0.55] |
| Wiwattanathum 2016 | 6 | 28 | 10 | 32 | 2.6% | 0.69 [0.29, 1.65] |
| Zadori 2015 | 0 | 91 | 22 | 123 | 0.5% | 0.03 [0.00, 0.49] |
| Total (95% CI) | | 5141 | | 10166 | 100.0% | 1.10 [0.89, 1.37] |
| Total events | 905 | | 1740 | | | |

Heterogeneity: Tau² = 0.26; Chi² = 213.34, df = 34 (P < 0.00001); I² = 84%
Test for overall effect: Z = 0.88 (P = 0.38)

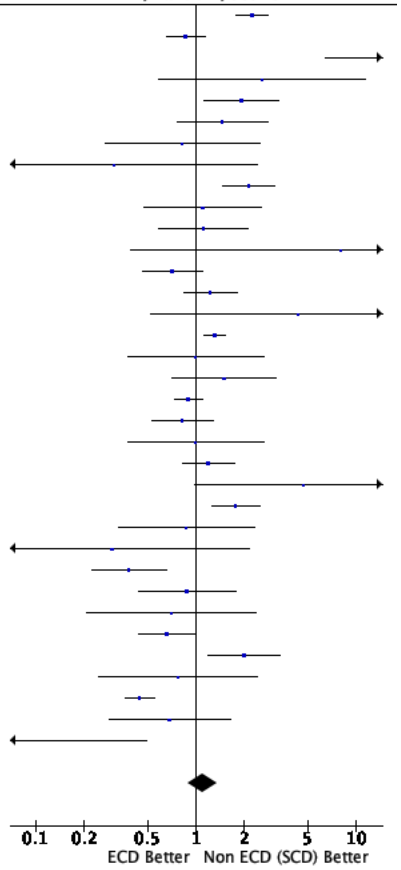


## Hospital readmission

| Study or Subgroup | ECD Better Events | ECD Better Total | Non ECD (SCD) Better Events | Non ECD (SCD) Better Total | Weight | Risk Ratio M-H, Random, 95% CI |
|---|---|---|---|---|---|---|
| Bonsignore 2019 | 18 | 73 | 11 | 42 | 12.5% | 0.94 [0.49, 1.80] |
| Farney 2008 | 48 | 138 | 54 | 178 | 14.9% | 1.15 [0.83, 1.58] |
| Mezrich 2012 | 152 | 201 | 128 | 358 | 15.6% | 2.12 [1.80, 2.48] |
| Moore 2007 | 23 | 61 | 20 | 53 | 13.9% | 1.00 [0.62, 1.60] |
| Saidi 2007 | 15 | 44 | 24 | 163 | 13.3% | 2.32 [1.33, 4.02] |
| Stratta 2016 | 144 | 571 | 149 | 351 | 15.5% | 0.59 [0.49, 0.72] |
| Sundberg 2004 | 20 | 37 | 30 | 53 | 14.5% | 0.95 [0.65, 1.40] |
| Total (95% CI) | | 1125 | | 1198 | 100.0% | 1.17 [0.71, 1.92] |
| Total events | 420 | | 416 | | | |

Heterogeneity: Tau² = 0.41; Chi² = 113.41, df = 6 (P < 0.00001); I² = 95%
Test for overall effect: Z = 0.60 (P = 0.55)

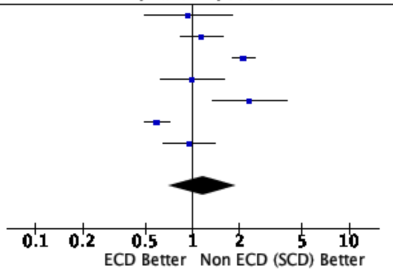

## 3. **Evidence Summary:** Older donors compared to younger donors for Renal transplant

| Certainty assessment | | | | | | | № of patients | | Effect | | Certainty | Importance |
|---|---|---|---|---|---|---|---|---|---|---|---|---|
| № of studies | Study design | Risk of bias | Inconsistency | Indirectness | Imprecision | Other considerations | older donors | younger donors | Relative (95% CI) | Absolute (95% CI) | | |
| **Mortality** | | | | | | | | | | | | |
| 11 | non-randomized studies | serious[a] | serious[b] | not serious | not serious | none | 1784/13116 (13.6%) | 2824/39493 (7.2%) | **RR 1.53** (1.15 to 2.02) | **38 more per 1,000** (from 11 more to 73 more) | ⊕◯◯◯ Very low | CRITICAL |
| **Death Censored Graft Survival** | | | | | | | | | | | | |
| 14 | non-randomized studies | serious[a] | serious[b] | not serious | not serious | none | 4636/5723 (81.0%) | 24607/28084 (87.6%) | **RR 0.97** (0.94 to 1.00) | **26 fewer per 1,000** (from 53 fewer to 0 fewer) | ⊕◯◯◯ Very low | CRITICAL |
| **Acute Rejection** | | | | | | | | | | | | |
| 16 | non-randomized studies | serious[a] | not serious | not serious | not serious | none | 459/1766 (26.0%) | 1974/9193 (21.5%) | **RR 1.18** (1.02 to 1.37) | **39 more per 1,000** (from 4 more to 79 more) | ⊕◯◯◯ Very low | CRITICAL |
| **Patient Survival** | | | | | | | | | | | | |
| 23 | non-randomized studies | serious[a] | serious[b] | not serious | not serious | none | 10055/14172 (70.9%) | 63053/82343 (76.6%) | **RR 0.95** (0.93 to 0.98) | **38 fewer per 1,000** (from 54 fewer to 15 fewer) | ⊕◯◯◯ Very low | CRITICAL |
| **Graft Survival** | | | | | | | | | | | | |
| 35 | non-randomized studies | serious[a] | serious[b] | not serious | not serious | none | 12388/18286 (67.7%) | 104938/132563 (79.2%) | **RR 0.88** (0.86 to 0.91) | **95 fewer per 1,000** (from 111 fewer to 71 fewer) | ⊕◯◯◯ Very low | CRITICAL |
| **Delayed Graft Function** | | | | | | | | | | | | |
| 27 | non-randomized studies | serious[a] | serious[b] | not serious | not serious | none | 1245/3804 (32.7%) | 4072/17346 (23.5%) | **RR 1.29** (1.12 to 1.48) | **68 more per 1,000** (from 28 more to 113 more) | ⊕◯◯◯ Very low | IMPORTANT |
| **Rejection** | | | | | | | | | | | | |
| 3 | non-randomized studies | serious[a] | serious | not serious | serious[c] | none | 65/190 (34.2%) | 240/990 (24.2%) | **RR 1.23** (0.96 to 1.57) | **56 more per 1,000** (from 10 fewer to 138 more) | ⊕◯◯◯ Very low | IMPORTANT |

**CI:** confidence interval; **RR:** risk ratio

**Explanations**

a. Rated down for ROB given high risk of bias across studies
b. Rated down for heterogeneity
c. Rated down for wide confidence intervals that include the line of no effect

# Forest Plots: Young Versus Old Donors

## Patient Survival

| Study or Subgroup | Older Donors Events | Older Donors Total | Younger Donors Events | Younger Donors Total | Weight | Risk Ratio M-H, Random, 95% CI |
|---|---|---|---|---|---|---|
| **1.4.1 At 1 year** | | | | | | |
| Basar 1999 | 35 | 40 | 173 | 190 | 1.8% | 0.96 [0.85, 1.09] |
| Carmellini 2000 | 44 | 49 | 207 | 216 | 2.1% | 0.94 [0.85, 1.03] |
| Collins 2009 | 518 | 540 | 3390 | 3489 | 2.8% | 0.99 [0.97, 1.01] |
| Di Cocco 2011 | 30 | 32 | 172 | 176 | 2.2% | 0.96 [0.87, 1.05] |
| Favi 2018 | 38 | 40 | 62 | 72 | 1.9% | 1.10 [0.98, 1.24] |
| Ferreira 2017 | 105 | 111 | 255 | 266 | 2.6% | 0.99 [0.94, 1.04] |
| Galaeno 2010 | 186 | 201 | 150 | 153 | 2.7% | 0.94 [0.90, 0.99] |
| Han 2020 | 203 | 217 | 758 | 789 | 2.7% | 0.97 [0.94, 1.01] |
| Kumar 1998 | 22 | 24 | 25 | 25 | 1.6% | 0.92 [0.80, 1.06] |
| Olaverri 2011 | 105 | 112 | 395 | 400 | 2.6% | 0.95 [0.90, 1.00] |
| Peters Sengers 2017 | 336 | 385 | 3056 | 3215 | 2.7% | 0.92 [0.88, 0.95] |
| Rao 1990 | 43 | 48 | 548 | 615 | 2.1% | 1.01 [0.91, 1.11] |
| Saudan 2001 | 67 | 68 | 242 | 247 | 2.8% | 1.01 [0.97, 1.04] |
| Sekta 2016 | 93 | 99 | 416 | 436 | 2.6% | 0.98 [0.93, 1.04] |
| Snoejis 2008 | 49 | 52 | 141 | 147 | 2.4% | 0.98 [0.91, 1.06] |
| Tekin 2015 | 268 | 294 | 2262 | 2339 | 2.7% | 0.94 [0.91, 0.98] |
| Subtotal (95% CI) | | 2312 | | 12775 | 38.2% | 0.97 [0.95, 0.99] |
| Total events | 2142 | | 12252 | | | |
| Heterogeneity: $Tau^2 = 0.00$; $Chi^2 = 30.42$, df = 15 (P = 0.01); $I^2 = 51\%$ | | | | | | |
| Test for overall effect: Z = 3.32 (P = 0.0009) | | | | | | |
| **1.4.2 at 3 years** | | | | | | |
| Carmellini 2000 | 42 | 49 | 206 | 216 | 1.9% | 0.90 [0.80, 1.01] |
| Di Cocco 2011 | 30 | 32 | 172 | 176 | 2.2% | 0.96 [0.87, 1.05] |
| Ferreira 2017 | 102 | 111 | 239 | 266 | 2.4% | 1.02 [0.96, 1.10] |
| Galaeno 2010 | 181 | 201 | 148 | 153 | 2.6% | 0.93 [0.88, 0.98] |
| Han 2020 | 200 | 217 | 749 | 789 | 2.7% | 0.97 [0.93, 1.01] |
| Kumar 1998 | 22 | 24 | 25 | 25 | 1.6% | 0.92 [0.80, 1.06] |
| Morrissey 2004 | 50 | 55 | 252 | 269 | 2.2% | 0.97 [0.89, 1.06] |
| Olaverri 2011 | 95 | 112 | 390 | 400 | 2.3% | 0.87 [0.80, 0.94] |
| Rao 1990 | 39 | 48 | 498 | 615 | 1.6% | 1.00 [0.87, 1.16] |
| Tekin 2015 | 260 | 294 | 2222 | 2339 | 2.7% | 0.93 [0.89, 0.97] |
| Subtotal (95% CI) | | 1143 | | 5248 | 22.1% | 0.95 [0.92, 0.98] |
| Total events | 1021 | | 4901 | | | |
| Heterogeneity: $Tau^2 = 0.00$; $Chi^2 = 13.60$, df = 9 (P = 0.14); $I^2 = 34\%$ | | | | | | |
| Test for overall effect: Z = 3.69 (P = 0.0002) | | | | | | |
| **1.4.3 At 5 years** | | | | | | |
| Basar 1999 | 29 | 40 | 149 | 190 | 1.1% | 0.92 [0.75, 1.13] |
| Bilgin 1998 | 127 | 155 | 558 | 649 | 2.3% | 0.95 [0.88, 1.03] |
| Carmellini 2000 | 39 | 49 | 201 | 216 | 1.6% | 0.86 [0.74, 0.99] |
| Collins 2009 | 470 | 540 | 3196 | 3489 | 2.8% | 0.95 [0.92, 0.98] |
| Di Cocco 2011 | 30 | 32 | 170 | 176 | 2.1% | 0.97 [0.88, 1.07] |
| Favi 2018 | 34 | 40 | 48 | 72 | 1.1% | 1.27 [1.03, 1.57] |
| Ferreira 2017 | 99 | 111 | 231 | 266 | 2.3% | 1.03 [0.95, 1.11] |
| Galaeno 2010 | 171 | 201 | 146 | 153 | 2.4% | 0.89 [0.83, 0.95] |
| Gerbase De Lima 2020 | 867 | 1027 | 3819 | 4330 | 2.8% | 0.96 [0.93, 0.98] |
| Hermanowicz 2016 | 271 | 358 | 6349 | 7207 | 2.5% | 0.86 [0.81, 0.91] |
| Kumar 1998 | 21 | 24 | 25 | 25 | 1.4% | 0.88 [0.74, 1.04] |
| Messa 2006 | 184 | 198 | 190 | 198 | 2.6% | 0.97 [0.92, 1.02] |
| Messina 2017 | 490 | 556 | 80 | 91 | 2.3% | 1.00 [0.92, 1.09] |
| Peters Sengers 2017 | 206 | 385 | 2659 | 3215 | 2.1% | 0.65 [0.59, 0.71] |
| Saudan 2001 | 62 | 68 | 230 | 247 | 2.3% | 0.98 [0.90, 1.06] |
| Snoejis 2008 | 43 | 52 | 128 | 147 | 1.6% | 0.95 [0.83, 1.09] |
| Subtotal (95% CI) | | 3836 | | 20671 | 33.3% | 0.93 [0.89, 0.98] |
| Total events | 3143 | | 18179 | | | |
| Heterogeneity: $Tau^2 = 0.01$; $Chi^2 = 112.86$, df = 15 (P < 0.00001); $I^2 = 87\%$ | | | | | | |
| Test for overall effect: Z = 2.90 (P = 0.004) | | | | | | |
| **1.4.4 at 10 years** | | | | | | |
| Bilgin 1998 | 115 | 155 | 506 | 649 | 2.0% | 0.95 [0.86, 1.05] |
| Keith 2004 | 3578 | 6658 | 27064 | 42753 | 2.8% | 0.85 [0.83, 0.87] |
| Saudan 2001 | 56 | 68 | 151 | 247 | 1.5% | 1.35 [1.16, 1.56] |
| Subtotal (95% CI) | | 6881 | | 43649 | 6.4% | 1.02 [0.81, 1.28] |
| Total events | 3749 | | 27721 | | | |
| Heterogeneity: $Tau^2 = 0.04$; $Chi^2 = 40.60$, df = 2 (P < 0.00001); $I^2 = 95\%$ | | | | | | |
| Test for overall effect: Z = 0.15 (P = 0.88) | | | | | | |
| **1.4.5 Overall** | | | | | | |
| Subtotal (95% CI) | | 0 | | 0 | | Not estimable |
| Total events | 0 | | 0 | | | |
| Heterogeneity: Not applicable | | | | | | |
| Test for overall effect: Not applicable | | | | | | |
| Total (95% CI) | | 14172 | | 82343 | 100.0% | 0.95 [0.93, 0.98] |
| Total events | 10055 | | 63053 | | | |
| Heterogeneity: $Tau^2 = 0.01$; $Chi^2 = 434.55$, df = 44 (P < 0.00001); $I^2 = 90\%$ | | | | | | |
| Test for overall effect: Z = 3.54 (P = 0.0004) | | | | | | |
| Test for subgroup differences: $Chi^2 = 3.59$, df = 3 (P = 0.31), $I^2 = 16.4\%$ | | | | | | |

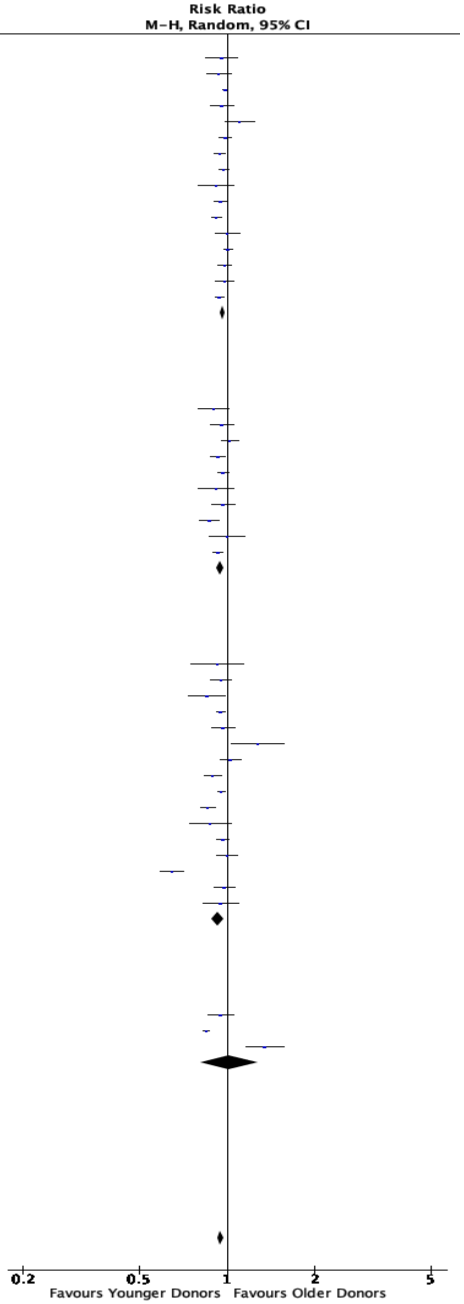

# Graft Survival

| | Older Donors | | Younger Donors | | | Risk Ratio |
|---|---|---|---|---|---|---|
| Study or Subgroup | Events | Total | Events | Total | Weight | M-H, Random, 95% CI |
| **1.6.1 At 1 year** | | | | | | |
| Akoh 2013 | 35 | 45 | 90 | 102 | 1.3% | 0.88 [0.74, 1.05] |
| Basar 1999 | 29 | 40 | 165 | 190 | 1.2% | 0.83 [0.68, 1.02] |
| Cohen 1997 | 1712 | 2255 | 20215 | 23366 | 2.1% | 0.88 [0.86, 0.90] |
| Collins 2009 | 453 | 540 | 3159 | 3489 | 2.1% | 0.93 [0.89, 0.96] |
| Di Cocco 2011 | 29 | 32 | 169 | 172 | 1.7% | 0.92 [0.82, 1.03] |
| Donnelly 1990 | 21 | 34 | 95 | 107 | 0.8% | 0.70 [0.53, 0.91] |
| Favi 2018 | 34 | 40 | 42 | 72 | 1.0% | 1.46 [1.15, 1.84] |
| Ferreira 2017 | 102 | 111 | 235 | 266 | 1.9% | 1.04 [0.97, 1.12] |
| Foster 1988 | 15 | 29 | 71 | 101 | 0.5% | 0.74 [0.51, 1.07] |
| Galaeno 2010 | 171 | 201 | 132 | 153 | 1.8% | 0.99 [0.91, 1.07] |
| Kim 1998 | 43 | 75 | 64 | 73 | 1.1% | 0.65 [0.53, 0.81] |
| Kumar 1998 | 19 | 24 | 25 | 25 | 1.1% | 0.80 [0.64, 0.99] |
| Kwon 2004 | 160 | 172 | 398 | 426 | 2.0% | 1.00 [0.95, 1.04] |
| Lloveras 2015 | 772 | 915 | 3729 | 4139 | 2.1% | 0.94 [0.91, 0.96] |
| Mizatani 1997 | 78 | 102 | 161 | 185 | 1.6% | 0.88 [0.78, 0.99] |
| Morling 1975 | 23 | 76 | 133 | 275 | 0.6% | 0.63 [0.44, 0.90] |
| Morrissey 2004 | 50 | 55 | 254 | 269 | 1.8% | 0.96 [0.88, 1.05] |
| Nakatani 2001 | 9 | 15 | 35 | 38 | 0.5% | 0.65 [0.43, 0.99] |
| Olaverri 2011 | 97 | 112 | 361 | 400 | 1.9% | 0.96 [0.89, 1.04] |
| Pessione 2003 | 489 | 554 | 6092 | 6665 | 2.1% | 0.97 [0.94, 1.00] |
| Peters Sengers 2017 | 300 | 385 | 2790 | 3215 | 2.0% | 0.90 [0.85, 0.95] |
| Pirsch 1992 | 46 | 49 | 422 | 477 | 1.9% | 1.06 [0.98, 1.15] |
| Rao 1990 | 34 | 48 | 473 | 615 | 1.2% | 0.92 [0.76, 1.11] |
| Saudan 2001 | 61 | 68 | 207 | 247 | 1.8% | 1.07 [0.97, 1.18] |
| Sekta 2016 | 89 | 99 | 405 | 436 | 1.9% | 0.97 [0.90, 1.04] |
| Shaheen 2010 | 35 | 43 | 427 | 481 | 1.5% | 0.92 [0.79, 1.06] |
| Snoejis 2006 | 18 | 24 | 134 | 176 | 0.9% | 0.99 [0.77, 1.26] |
| Vivas 1997 | 20 | 31 | 25 | 31 | 0.7% | 0.80 [0.59, 1.09] |
| Wahlberg 1990 | 19 | 37 | 12 | 49 | 0.3% | 2.10 [1.17, 3.76] |
| Subtotal (95% CI) | | 6211 | | 46240 | 41.4% | 0.94 [0.91, 0.97] |
| Total events | 4963 | | 40520 | | | |
| Heterogeneity: $Tau^2 = 0.00$; $Chi^2 = 131.87$, df = 28 ($P < 0.00001$); $I^2 = 79\%$ | | | | | | |
| Test for overall effect: Z = 3.59 (P = 0.0003) | | | | | | |
| **1.6.2 at 3 years** | | | | | | |
| Cohen 1997 | 1418 | 2255 | 18019 | 23366 | 2.1% | 0.82 [0.79, 0.84] |
| Di Cocco 2011 | 28 | 32 | 167 | 176 | 1.5% | 0.92 [0.81, 1.06] |
| Donnelly 1990 | 19 | 34 | 93 | 107 | 0.7% | 0.64 [0.47, 0.87] |
| Ferreira 2017 | 98 | 111 | 231 | 266 | 1.9% | 1.02 [0.94, 1.10] |
| Foster 1988 | 10 | 29 | 59 | 101 | 0.3% | 0.59 [0.35, 1.00] |
| Galaeno 2010 | 146 | 201 | 127 | 153 | 1.7% | 0.88 [0.78, 0.98] |
| Kim 1998 | 43 | 75 | 63 | 75 | 1.1% | 0.68 [0.55, 0.85] |
| Kumar 1998 | 15 | 24 | 24 | 25 | 0.7% | 0.65 [0.47, 0.90] |
| Kwon 2004 | 147 | 172 | 384 | 426 | 1.9% | 0.95 [0.88, 1.02] |
| Mizatani 1997 | 56 | 102 | 144 | 185 | 1.2% | 0.71 [0.58, 0.85] |
| Morrissey 2004 | 48 | 55 | 230 | 269 | 1.7% | 1.02 [0.91, 1.14] |
| Navarro 2011 | 23 | 32 | 92 | 104 | 1.0% | 0.81 [0.65, 1.02] |
| Olaverri 2011 | 88 | 112 | 346 | 400 | 1.7% | 0.91 [0.82, 1.01] |
| Pessione 2003 | 447 | 554 | 5732 | 6665 | 2.0% | 0.94 [0.90, 0.98] |
| Pirsch 1992 | 36 | 49 | 396 | 477 | 1.3% | 0.88 [0.74, 1.05] |
| Rao 1990 | 26 | 48 | 399 | 615 | 0.9% | 0.83 [0.64, 1.09] |
| Shaheen 2010 | 33 | 43 | 403 | 481 | 1.3% | 0.92 [0.77, 1.08] |
| Subtotal (95% CI) | | 3928 | | 33891 | 23.1% | 0.87 [0.82, 0.92] |
| Total events | 2681 | | 26909 | | | |
| Heterogeneity: $Tau^2 = 0.01$; $Chi^2 = 85.08$, df = 16 ($P < 0.00001$); $I^2 = 81\%$ | | | | | | |
| Test for overall effect: Z = 4.50 (P < 0.00001) | | | | | | |
| **1.6.3 At 5 years** | | | | | | |
| Akoh 2013 | 32 | 45 | 81 | 102 | 1.1% | 0.90 [0.73, 1.11] |
| Basar 1999 | 21 | 40 | 125 | 190 | 0.7% | 0.80 [0.58, 1.09] |
| Bilgin 1998 | 104 | 155 | 467 | 649 | 1.6% | 0.93 [0.83, 1.05] |
| Cohen 1997 | 1195 | 2255 | 16186 | 23366 | 2.1% | 0.77 [0.74, 0.80] |
| Collins 2009 | 334 | 549 | 2781 | 3489 | 1.9% | 0.76 [0.71, 0.82] |
| Di Cocco 2011 | 28 | 32 | 166 | 176 | 1.5% | 0.93 [0.81, 1.06] |
| Favi 2018 | 30 | 40 | 28 | 72 | 0.6% | 1.93 [1.37, 2.71] |
| Ferreira 2017 | 93 | 111 | 231 | 266 | 1.8% | 0.96 [0.88, 1.06] |
| Galaeno 2010 | 146 | 201 | 124 | 153 | 1.7% | 0.90 [0.80, 1.00] |
| Hermanowicz 2016 | 212 | 358 | 5412 | 7207 | 1.8% | 0.79 [0.72, 0.86] |
| Kumar 1998 | 13 | 24 | 22 | 25 | 0.5% | 0.62 [0.41, 0.91] |
| Kwon 2004 | 134 | 172 | 367 | 426 | 1.8% | 0.90 [0.83, 0.99] |
| Lloveras 2015 | 558 | 915 | 3212 | 4139 | 2.0% | 0.79 [0.74, 0.83] |
| Magott–Procelewska 2018 | 44 | 58 | 264 | 287 | 1.5% | 0.82 [0.71, 0.96] |
| Messina 2017 | 413 | 556 | 67 | 91 | 1.6% | 1.01 [0.88, 1.15] |
| Mizatani 1997 | 46 | 102 | 130 | 185 | 1.0% | 0.64 [0.51, 0.81] |
| Morling 1975 | 15 | 76 | 107 | 275 | 0.4% | 0.51 [0.31, 0.82] |
| Nakatani 2001 | 6 | 15 | 29 | 38 | 0.2% | 0.52 [0.28, 1.00] |
| Navarro 2011 | 22 | 32 | 84 | 104 | 0.9% | 0.85 [0.66, 1.09] |
| Peters Sengers 2017 | 185 | 385 | 2346 | 3215 | 1.7% | 0.66 [0.59, 0.73] |
| Saudan 2001 | 52 | 68 | 165 | 247 | 1.4% | 1.14 [0.98, 1.34] |
| Snoejis 2006 | 12 | 24 | 120 | 176 | 0.5% | 0.73 [0.49, 1.11] |
| Vivas 1997 | 10 | 31 | 321 | 527 | | Not estimable |
| Subtotal (95% CI) | | 6213 | | 44878 | 28.3% | 0.85 [0.79, 0.91] |
| Total events | 3695 | | 32514 | | | |
| Heterogeneity: $Tau^2 = 0.02$; $Chi^2 = 132.27$, df = 21 ($P < 0.00001$); $I^2 = 84\%$ | | | | | | |
| Test for overall effect: Z = 4.90 (P < 0.00001) | | | | | | |
| **1.6.4 at 10 years** | | | | | | |
| Bilgin 1998 | 75 | 155 | 350 | 649 | 1.3% | 0.90 [0.75, 1.07] |
| Lloveras 2015 | 367 | 915 | 2438 | 4139 | 1.9% | 0.68 [0.63, 0.74] |
| Magott–Procelewska 2018 | 31 | 58 | 224 | 287 | 0.9% | 0.68 [0.53, 0.88] |
| Nakatani 2001 | 3 | 15 | 27 | 38 | 0.1% | 0.28 [0.10, 0.79] |
| Saudan 2001 | 41 | 68 | 119 | 247 | 1.0% | 1.25 [0.99, 1.58] |
| Subtotal (95% CI) | | 1211 | | 5360 | 5.2% | 0.80 [0.61, 1.04] |
| Total events | 517 | | 3158 | | | |
| Heterogeneity: $Tau^2 = 0.07$; $Chi^2 = 32.30$, df = 4 ($P < 0.00001$); $I^2 = 88\%$ | | | | | | |
| Test for overall effect: Z = 1.67 (P = 0.10) | | | | | | |
| **1.6.5 Overall** | | | | | | |
| Sirchia 1998 | 532 | 723 | 1837 | 2194 | 2.0% | 0.88 [0.84, 0.92] |
| Subtotal (95% CI) | | 723 | | 2194 | 2.0% | 0.88 [0.84, 0.92] |
| Total events | 532 | | 1837 | | | |
| Heterogeneity: Not applicable | | | | | | |
| Test for overall effect: Z = 5.34 (P < 0.00001) | | | | | | |
| **Total (95% CI)** | | 18286 | | 132563 | 100.0% | 0.88 [0.86, 0.91] |
| Total events | 12388 | | 104938 | | | |
| Heterogeneity: $Tau^2 = 0.01$; $Chi^2 = 618.10$, df = 73 ($P < 0.00001$); $I^2 = 88\%$ | | | | | | |
| Test for overall effect: Z = 7.58 (P < 0.00001) | | | | | | |
| Test for subgroup differences: $Chi^2 = 12.21$, df = 4 (P = 0.02), $I^2 = 67.3\%$ | | | | | | |

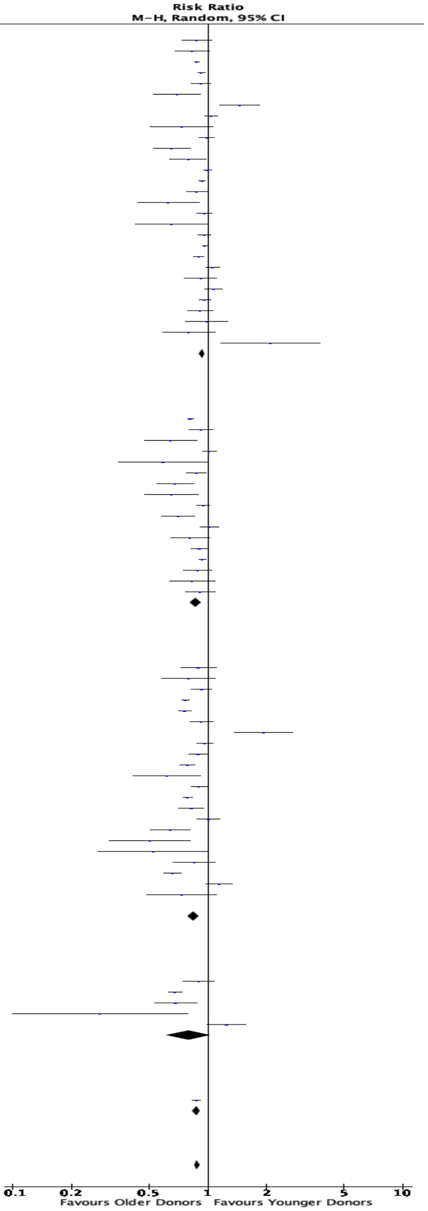

## Delayed Graft Function

| Study or Subgroup | Older Donors Events | Older Donors Total | Younger Donors Events | Younger Donors Total | Weight | Risk Ratio M-H, Random, 95% CI |
|---|---|---|---|---|---|---|
| Ballesteros 2020 | 4 | 18 | 9 | 57 | 1.3% | 1.41 [0.49, 4.03] |
| Basar 1999 | 19 | 40 | 57 | 190 | 3.9% | 1.58 [1.07, 2.34] |
| Bilgin 1998 | 0 | 0 | 0 | 0 | | Not estimable |
| Burrows 1999 | 8 | 22 | 35 | 90 | 2.7% | 0.94 [0.51, 1.72] |
| Buxeda 2021 | 54 | 172 | 11 | 41 | 3.0% | 1.17 [0.67, 2.03] |
| Carmellini 2000 | 20 | 49 | 68 | 216 | 3.9% | 1.30 [0.88, 1.92] |
| Collins 2009 | 192 | 540 | 670 | 3489 | 5.5% | 1.85 [1.62, 2.11] |
| Di Cocco 2011 | 5 | 32 | 36 | 176 | 1.8% | 0.76 [0.32, 1.80] |
| Favi 2018 | 17 | 49 | 50 | 72 | 3.8% | 0.50 [0.33, 0.76] |
| Han 2020 | 23 | 217 | 63 | 789 | 3.6% | 1.33 [0.84, 2.09] |
| Lim 2012 | 76 | 221 | 189 | 816 | 5.0% | 1.48 [1.19, 1.85] |
| Magott-Procelewska 2018 | 37 | 58 | 95 | 287 | 4.8% | 1.93 [1.49, 2.49] |
| Martins 2020 | 7 | 21 | 35 | 126 | 2.5% | 1.20 [0.62, 2.34] |
| Messina 2017 | 176 | 556 | 34 | 91 | 4.6% | 0.85 [0.63, 1.14] |
| Morrissey 2004 | 7 | 55 | 26 | 269 | 2.0% | 1.32 [0.60, 2.88] |
| Navarro 2011 | 15 | 32 | 23 | 104 | 3.2% | 2.12 [1.27, 3.55] |
| Oppenhemier 2004 | 186 | 478 | 831 | 2887 | 5.5% | 1.35 [1.19, 1.53] |
| Perez-Saez 2019 | 75 | 135 | 199 | 426 | 5.2% | 1.19 [0.99, 1.43] |
| Peters Sengers 2017 | 147 | 385 | 1041 | 3215 | 5.5% | 1.18 [1.03, 1.35] |
| Resende 2009 | 11 | 73 | 19 | 368 | 2.3% | 2.92 [1.45, 5.87] |
| Roselli 2007 | 39 | 57 | 29 | 127 | 4.1% | 3.00 [2.08, 4.32] |
| Saudan 2001 | 14 | 68 | 52 | 247 | 3.1% | 0.98 [0.58, 1.65] |
| Sekta 2016 | 28 | 99 | 136 | 436 | 4.3% | 0.91 [0.64, 1.28] |
| Snoejis 2006 | 12 | 24 | 104 | 175 | 3.8% | 0.84 [0.55, 1.28] |
| Snoejis 2008 | 26 | 52 | 96 | 147 | 4.6% | 0.77 [0.57, 1.03] |
| Tekin 2015 | 14 | 294 | 105 | 2339 | 3.0% | 1.06 [0.62, 1.83] |
| Thornton 2011 | 13 | 19 | 47 | 117 | 4.0% | 1.70 [1.17, 2.48] |
| Wahlberg 1990 | 20 | 38 | 12 | 49 | 2.9% | 2.15 [1.21, 3.83] |
| Total (95% CI) | | 3804 | | 17346 | 100.0% | 1.29 [1.12, 1.48] |
| Total events | 1245 | | 4072 | | | |

Heterogeneity: $Tau^2 = 0.09$; $Chi^2 = 128.36$, df = 26 ($P < 0.00001$); $I^2 = 80\%$
Test for overall effect: Z = 3.58 (P = 0.0003)

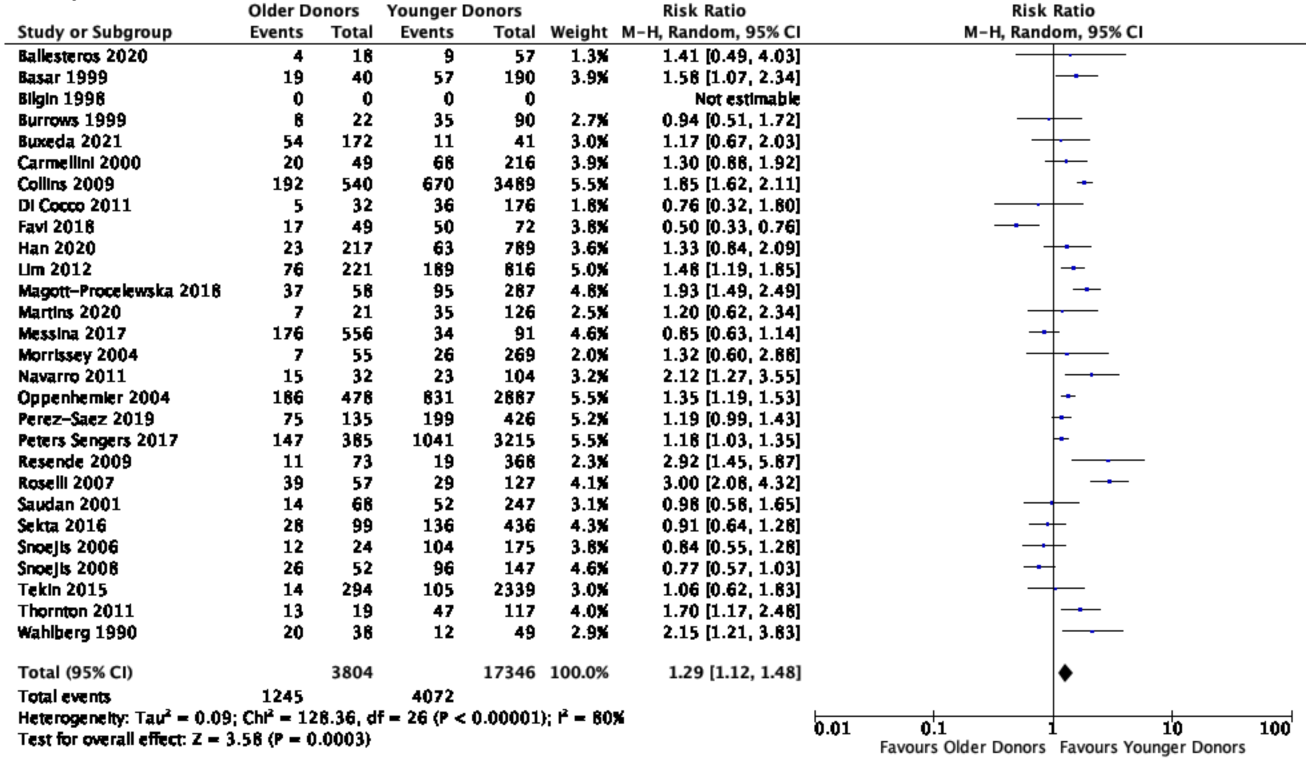


## Acute Rejection

| Study or Subgroup | Older Donors Events | Older Donors Total | Younger Donors Events | Younger Donors Total | Weight | Risk Ratio M-H, Random, 95% CI |
|---|---|---|---|---|---|---|
| Akoh 2013 | 12 | 40 | 15 | 96 | 3.9% | 1.92 [0.99, 3.73] |
| Burrows 1999 | 12 | 22 | 32 | 90 | 6.5% | 1.53 [0.96, 2.46] |
| Collins 2009 | 190 | 540 | 916 | 3489 | 18.2% | 1.34 [1.18, 1.52] |
| Di Cocco 2011 | 5 | 32 | 21 | 176 | 2.3% | 1.31 [0.53, 3.22] |
| Hricik 2013 | 11 | 41 | 8 | 77 | 2.7% | 2.58 [1.13, 5.91] |
| Martins 2020 | 3 | 21 | 14 | 126 | 1.5% | 1.29 [0.40, 4.09] |
| Messa 2006 | 57 | 198 | 62 | 198 | 11.1% | 0.92 [0.68, 1.24] |
| Morrissey 2004 | 10 | 55 | 58 | 269 | 4.5% | 0.84 [0.46, 1.54] |
| Nakatani 2001 | 5 | 15 | 15 | 38 | 2.7% | 0.84 [0.37, 1.91] |
| Navarro 2011 | 7 | 32 | 13 | 104 | 2.7% | 1.75 [0.76, 4.01] |
| Perez-Saez 2019 | 4 | 135 | 27 | 426 | 1.8% | 0.47 [0.17, 1.31] |
| Peters Sengers 2017 | 64 | 385 | 523 | 3215 | 13.5% | 1.02 [0.81, 1.30] |
| Resende 2009 | 26 | 73 | 135 | 368 | 9.9% | 0.97 [0.69, 1.36] |
| Roselli 2007 | 6 | 57 | 12 | 127 | 2.2% | 1.11 [0.44, 2.82] |
| Saudan 2001 | 37 | 68 | 91 | 247 | 12.2% | 1.48 [1.13, 1.94] |
| Snoejis 2008 | 10 | 52 | 32 | 147 | 4.2% | 0.88 [0.47, 1.67] |
| Total (95% CI) | | 1766 | | 9193 | 100.0% | 1.18 [1.02, 1.37] |
| Total events | 459 | | 1974 | | | |

Heterogeneity: $Tau^2 = 0.03$; $Chi^2 = 24.84$, df = 15 ($P = 0.05$); $I^2 = 40\%$
Test for overall effect: Z = 2.25 (P = 0.02)

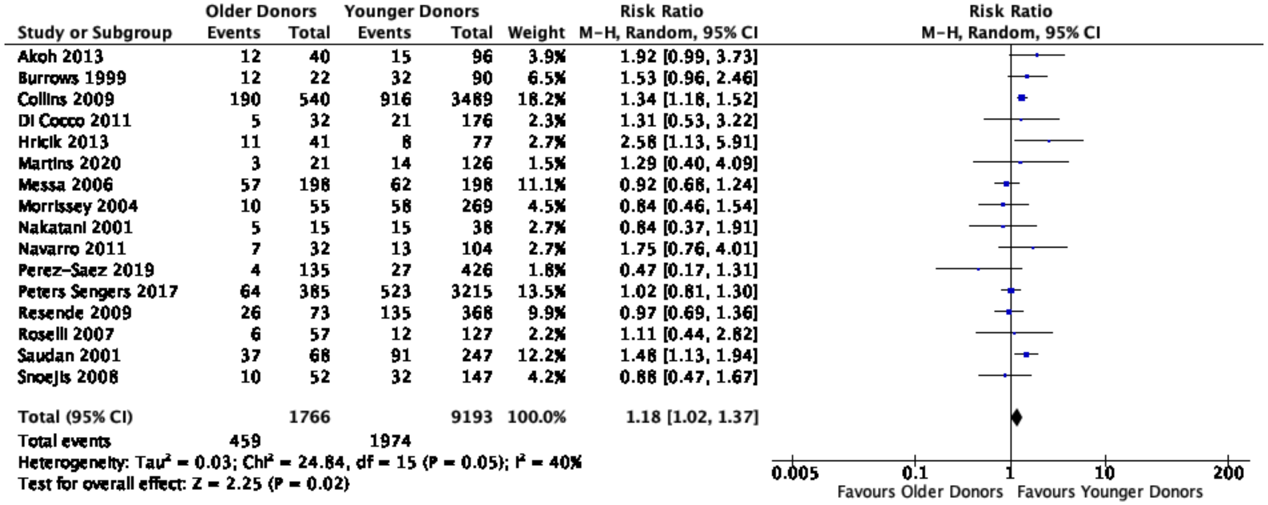

## Death Censored Graft Survival

| Study or Subgroup | Older Donors Events | Older Donors Total | Younger Donors Events | Younger Donors Total | Weight | Risk Ratio M-H, Random, 95% CI |
|---|---|---|---|---|---|---|
| **1.2.1 at 1 year** | | | | | | |
| Collins 2009 | 473 | 540 | 3253 | 3489 | 5.6% | 0.94 [0.91, 0.97] |
| Favi 2018 | 36 | 40 | 55 | 72 | 2.3% | 1.18 [1.00, 1.39] |
| Ferreira 2017 | 102 | 111 | 250 | 266 | 4.9% | 0.98 [0.92, 1.04] |
| Han 2020 | 208 | 217 | 767 | 789 | 5.7% | 0.99 [0.96, 1.02] |
| Morrissey 2004 | 54 | 55 | 262 | 269 | 5.4% | 1.01 [0.97, 1.05] |
| Peters Sengers 2017 | 338 | 385 | 2906 | 3215 | 5.5% | 0.97 [0.93, 1.01] |
| Saudan 2001 | 58 | 68 | 222 | 247 | 3.6% | 0.95 [0.85, 1.06] |
| Snoejis 2008 | 32 | 35 | 116 | 147 | 3.0% | 1.16 [1.02, 1.32] |
| Tekin 2015 | 287 | 294 | 2281 | 2339 | 5.9% | 1.00 [0.98, 1.02] |
| Subtotal (95% CI) | | 1745 | | 10833 | 41.9% | 0.99 [0.96, 1.02] |
| Total events | 1588 | | 10112 | | | |
| Heterogeneity: Tau² = 0.00; Chi² = 27.17, df = 8 (P = 0.0007); I² = 71% | | | | | | |
| Test for overall effect: Z = 0.71 (P = 0.47) | | | | | | |
| **1.2.2 at 3 years** | | | | | | |
| Ferreira 2017 | 98 | 111 | 231 | 266 | 4.3% | 1.02 [0.94, 1.10] |
| Han 2020 | 206 | 217 | 760 | 789 | 5.6% | 0.99 [0.95, 1.02] |
| Morrissey 2004 | 54 | 55 | 254 | 269 | 5.3% | 1.04 [0.99, 1.09] |
| Tekin 2015 | 277 | 294 | 2227 | 2339 | 5.7% | 0.99 [0.96, 1.02] |
| Subtotal (95% CI) | | 677 | | 3663 | 20.9% | 1.00 [0.98, 1.03] |
| Total events | 635 | | 3472 | | | |
| Heterogeneity: Tau² = 0.00; Chi² = 4.13, df = 3 (P = 0.25); I² = 27% | | | | | | |
| Test for overall effect: Z = 0.06 (P = 0.96) | | | | | | |
| **1.2.3 at 5 years** | | | | | | |
| Collins 2009 | 383 | 540 | 3033 | 3489 | 5.1% | 0.82 [0.77, 0.86] |
| Favi 2018 | 33 | 40 | 45 | 72 | 1.5% | 1.32 [1.05, 1.66] |
| Gerbase De Lima 2020 | 795 | 1027 | 3768 | 4330 | 5.6% | 0.89 [0.86, 0.92] |
| Lim 2012 | 0 | 0 | 0 | 0 | | Not estimable |
| Messina 2017 | 464 | 556 | 75 | 91 | 3.7% | 1.01 [0.91, 1.12] |
| Peters Sengers 2017 | 311 | 385 | 2722 | 3215 | 5.2% | 0.95 [0.91, 1.00] |
| Resende 2009 | 56 | 73 | 324 | 368 | 3.0% | 0.87 [0.76, 0.99] |
| Saudan 2001 | 48 | 68 | 200 | 247 | 2.3% | 0.87 [0.74, 1.03] |
| Snoejis 2008 | 27 | 52 | 103 | 147 | 1.1% | 0.74 [0.56, 0.98] |
| Subtotal (95% CI) | | 2741 | | 11959 | 27.4% | 0.91 [0.85, 0.98] |
| Total events | 2117 | | 10270 | | | |
| Heterogeneity: Tau² = 0.01; Chi² = 36.16, df = 7 (P < 0.00001); I² = 81% | | | | | | |
| Test for overall effect: Z = 2.55 (P = 0.01) | | | | | | |
| **1.2.4 at 10 years** | | | | | | |
| Resende 2009 | 50 | 73 | 302 | 368 | 2.4% | 0.83 [0.71, 0.98] |
| Saudan 2001 | 46 | 68 | 170 | 247 | 2.0% | 0.98 [0.82, 1.18] |
| Subtotal (95% CI) | | 141 | | 615 | 4.4% | 0.90 [0.77, 1.06] |
| Total events | 96 | | 472 | | | |
| Heterogeneity: Tau² = 0.01; Chi² = 1.70, df = 1 (P = 0.19); I² = 41% | | | | | | |
| Test for overall effect: Z = 1.29 (P = 0.20) | | | | | | |
| **1.2.5 Overall** | | | | | | |
| Lim 2012 | 36 | 221 | 91 | 816 | 0.7% | 1.46 [1.02, 2.09] |
| Messa 2006 | 164 | 198 | 190 | 198 | 4.7% | 0.86 [0.81, 0.93] |
| Subtotal (95% CI) | | 419 | | 1014 | 5.4% | 1.10 [0.55, 2.23] |
| Total events | 200 | | 281 | | | |
| Heterogeneity: Tau² = 0.24; Chi² = 14.99, df = 1 (P = 0.0001); I² = 93% | | | | | | |
| Test for overall effect: Z = 0.28 (P = 0.78) | | | | | | |
| **Total (95% CI)** | | 5723 | | 28084 | 100.0% | 0.97 [0.94, 1.00] |
| Total events | 4636 | | 24607 | | | |
| Heterogeneity: Tau² = 0.00; Chi² = 195.34, df = 24 (P < 0.00001); I² = 88% | | | | | | |
| Test for overall effect: Z = 2.00 (P = 0.05) | | | | | | |
| Test for subgroup differences: Chi² = 7.32, df = 4 (P = 0.12), I² = 45.4% | | | | | | |

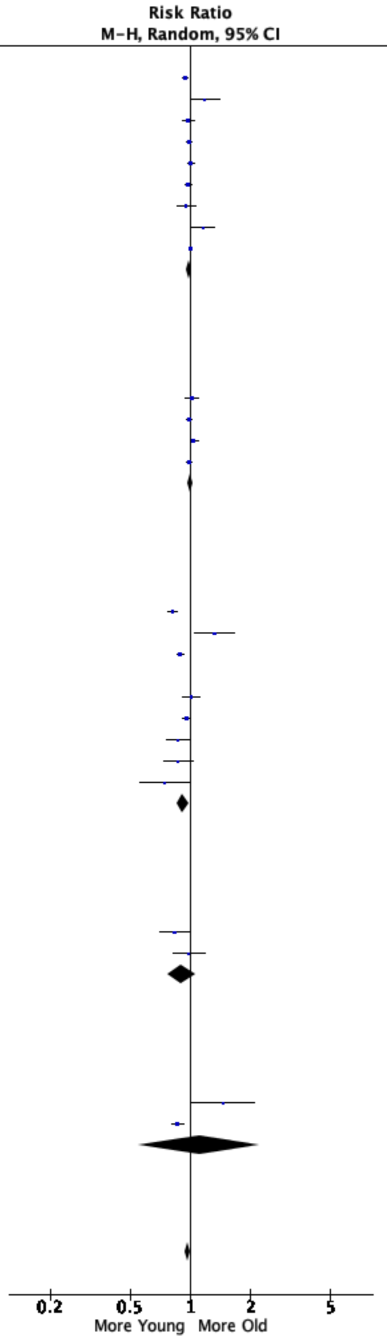

## Overall Rejection

| Study or Subgroup | Older Donors Events | Older Donors Total | Younger Donors Events | Younger Donors Total | Weight | Risk Ratio M-H, Random, 95% CI |
|---|---|---|---|---|---|---|
| Basar 1999 | 12 | 40 | 46 | 190 | 20.5% | 1.24 [0.72, 2.12] |
| Mizatani 1997 | 43 | 102 | 59 | 185 | 61.4% | 1.32 [0.97, 1.80] |
| Rao 1990 | 10 | 48 | 135 | 615 | 18.1% | 0.95 [0.54, 1.68] |
| Total (95% CI) | | 190 | | 990 | 100.0% | 1.23 [0.96, 1.57] |
| Total events | 65 | | 240 | | | |

Heterogeneity: Tau² = 0.00; Chi² = 1.01, df = 2 (P = 0.60); I² = 0%

Test for overall effect: Z = 1.66 (P = 0.10)

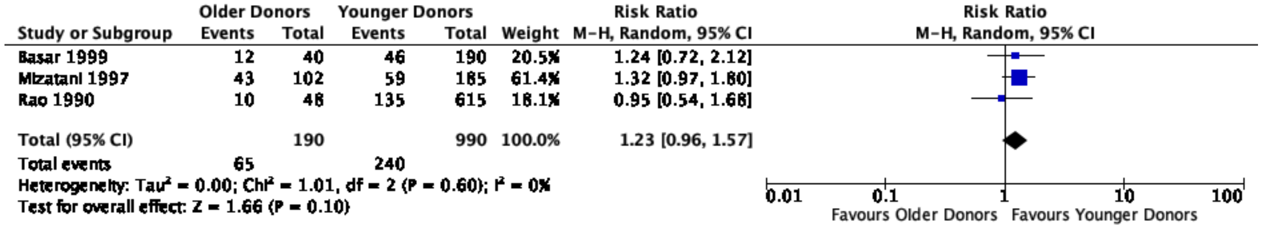

# 4. **Evidence Summary:** AKI kidney compared to no AKI Kidney for Renal Transplant

| Certainty assessment | | | | | | | № of patients | | Effect | | Certainty | Importance |
|---|---|---|---|---|---|---|---|---|---|---|---|---|
| № of studies | Study design | Risk of bias | Inconsistency | Indirectness | Imprecision | Other considerations | a kidney with AKI | no AKI | Relative (95% CI) | Absolute (95% CI) | | |
| **Mortality AKI vs nonAKI - Mortality 1 year** | | | | | | | | | | | | |
| 3 | non-randomized studies | serious | serious[a] | not serious | serious[b] | none | 47/703 (6.7%) | 104/1661 (6.3%) | **RR 0.75** (0.33 to 1.71) | **16 fewer per 1,000** (from 42 fewer to 44 more) | ⊕◯◯◯ Very low | CRITICAL |
| **Mortality AKI vs nonAKI** | | | | | | | | | | | | |
| 11 | non-randomized studies | serious | serious | not serious | serious | none | 89/1687 (5.3%) | 170/2800 (6.1%) | **RR 0.80** (0.56 to 1.14) | **12 fewer per 1,000** (from 27 fewer to 8 more) | ⊕◯◯◯ Very low | CRITICAL |
| **Mortality AKI vs nonAKI - Mortatity 3 years** | | | | | | | | | | | | |
| 1 | non-randomized studies | serious | not serious | not serious | not serious | none | 5/55 (9.1%) | 10/110 (9.1%) | **RR 1.00** (0.36 to 2.78) | **0 fewer per 1,000** (from 58 fewer to 162 more) | ⊕◯◯◯ Very low | CRITICAL |
| **Mortality AKI vs nonAKI - Mortality follow up to 5 years** | | | | | | | | | | | | |
| 2 | non-randomized studies | serious | serious[a] | not serious | serious[b] | none[c] | 5/174 (2.9%) | 19/395 (4.8%) | **RR 0.79** (0.17 to 3.61) | **10 fewer per 1,000** (from 40 fewer to 126 more) | ⊕◯◯◯ Very low | CRITICAL |
| **Graft survival AKI vs nonAKI - 1 year Graft survival** | | | | | | | | | | | | |
| 14 | non-randomized studies | serious[d] | serious | not serious | not serious[e] | none | 2967/3372 (88.0%) | 11248/12700 (88.6%) | **RR 1.03** (0.98 to 1.08) | **27 more per 1,000** (from 18 fewer to 71 more) | ⊕◯◯◯ Very low | CRITICAL |
| **Graft survival AKI vs nonAKI - 3-year Graft survival** | | | | | | | | | | | | |
| 5 | non-randomized studies | serious[d] | serious | not serious | serious[e] | none | 1794/2134 (84.1%) | 8530/9984 (85.4%) | **RR 1.03** (0.97 to 1.09) | **26 more per 1,000** (from 26 fewer to 77 more) | ⊕◯◯◯ Very low | CRITICAL |
| **Graft survival AKI vs nonAKI - 5-year Graft survival** | | | | | | | | | | | | |

| | | | | | | | | | | | | |
|---|---|---|---|---|---|---|---|---|---|---|---|---|
| 7 | non-randomized studies | serious[d] | serious | not serious | serious[e] | none | 2116/2744 (77.1%) | 8264/10665 (77.5%) | **RR 0.98** (0.96 to 1.01) | **15 fewer per 1,000** (from 31 fewer to 8 more) | ⨁◯◯◯ Very low | CRITICAL |

**Graft survival AKI vs nonAKI - 10-year Graft survival**

| Certainty assessment | | | | | | | № of patients | | Effect | | Certainty | Importance |
|---|---|---|---|---|---|---|---|---|---|---|---|---|
| № of studies | Study design | Risk of bias | Inconsistency | Indirectness | Imprecision | Other considerations | a kidney with AKI | no AKI | Relative (95% CI) | Absolute (95% CI) | | |
| 2 | non-randomized studies | serious[d] | serious | not serious | serious | none | 125/173 (72.3%) | 155/229 (67.7%) | **RR 1.08** (0.95 to 1.23) | **54 more per 1,000** (from 34 fewer to 156 more) | ⊕◯◯◯ Very low | CRITICAL |
| **Graft failure/loss AKI vs nonAKI - Graft failure- 1 year** | | | | | | | | | | | | |
| 12 | non-randomized studies | serious[d] | serious[f] | not serious | not serious | none | 428/4210 (10.2%) | 1229/13785 (8.9%) | **RR 1.14** (1.02 to 1.27) | **12 more per 1,000** (from 2 more to 24 more) | ⊕◯◯◯ Very low | CRITICAL |
| **Graft failure/loss AKI vs nonAKI - Graft failure- 3 years** | | | | | | | | | | | | |
| 3 | non-randomized studies | serious[d] | serious[f] | not serious | serious[b] | none | 104/668 (15.6%) | 312/2011 (15.5%) | **RR 1.03** (0.84 to 1.26) | **5 more per 1,000** (from 25 fewer to 40 more) | ⊕◯◯◯ Very low | CRITICAL |
| **Acute rejection rate (up to a year) AKI vs nonAKI** | | | | | | | | | | | | |
| 24 | non-randomized studies | serious[d] | serious[f] | not serious | serious[b] | none | 661/2497 (26.5%) | 1193/4837 (24.7%) | **RR 1.02** (0.94 to 1.11) | **5 more per 1,000** (from 15 fewer to 27 more) | ⊕◯◯◯ Very low | IMPORTANT |
| **Hemodialysis posttransplant- AKI vs nonAKI** | | | | | | | | | | | | |
| 1 | non-randomized studies | serious[d] | not serious | not serious | very serious[b] | none | 14/33 (42.4%) | 18/65 (27.7%) | **RR 1.53** (0.88 to 2.68) | **147 more per 1,000** (from 33 fewer to 465 more) | ⊕◯◯◯ Very low | IMPORTANT |

**CI:** confidence interval; **RR:** risk ratio

## Explanations

a. Rated down one level for heterogeneity
b. Rated down for few number of events, and wide confidence intervals that include the line of no effect
c. Could not formally assess due to <10 studies
d. Rated down for possible double counting of events between studies
e. Rated down as it includes the line of no effect
f. Rated down for inconsistency of effect between studies.

## Forest Plots: AKI Versus Non AKI Donors

Graft Loss

| | AKI | | nonAKI | | | Risk Ratio | |
|---|---|---|---|---|---|---|---|
| Study or Subgroup | Events | Total | Events | Total | Weight | M-H, Random, 95% CI | Year |
| 2.8.1 Graft failure- 1 year | | | | | | | |
| Klein 2013 | 66 | 485 | 109 | 1033 | 14.2% | 1.29 [0.97, 1.72] | 2013 |
| Yu 2014 | 0 | 28 | 4 | 56 | 0.1% | 0.22 [0.01, 3.92] | 2014 |
| Hall 2015 | 55 | 585 | 150 | 1845 | 13.4% | 1.16 [0.86, 1.55] | 2015 |
| Wiwattanathum 2016 | 0 | 51 | 3 | 60 | 0.1% | 0.17 [0.01, 3.17] | 2016 |
| Jung 2016 | 1 | 12 | 1 | 15 | 0.2% | 1.25 [0.09, 17.98] | 2016 |
| Boffa 2017 | 202 | 1869 | 849 | 9350 | 55.2% | 1.19 [1.03, 1.38] | 2017 |
| Yu 2018 | 26 | 275 | 19 | 138 | 3.8% | 0.69 [0.39, 1.20] | 2018 |
| Torabi 2019 | 6 | 122 | 4 | 193 | 0.8% | 2.37 [0.68, 8.24] | 2019 |
| Kwon 2019 | 2 | 30 | 8 | 151 | 0.5% | 1.26 [0.28, 5.63] | 2019 |
| Park 2019 | 38 | 270 | 35 | 239 | 6.4% | 0.96 [0.63, 1.47] | 2019 |
| van der Windt 2020 | 5 | 96 | 25 | 435 | 1.3% | 0.91 [0.36, 2.31] | 2020 |
| Park 2021 | 27 | 387 | 22 | 270 | 4.0% | 0.86 [0.50, 1.47] | 2021 |
| Subtotal (95% CI) | | 4210 | | 13785 | 100.0% | 1.14 [1.02, 1.27] | |
| Total events | 428 | | 1229 | | | | |
| Heterogeneity: Tau² = 0.00; Chi² = 10.45, df = 11 (P = 0.49); I² = 0% | | | | | | | |
| Test for overall effect: Z = 2.38 (P = 0.02) | | | | | | | |
| 2.8.2 Graft failrue- 3 years | | | | | | | |
| Kumar 2006 | 5 | 55 | 14 | 110 | 4.4% | 0.71 [0.27, 1.88] | 2006 |
| Yu 2014 | 1 | 28 | 1 | 56 | 0.6% | 2.00 [0.13, 30.80] | 2014 |
| Hall 2015 | 98 | 585 | 297 | 1845 | 95.0% | 1.04 [0.84, 1.28] | 2015 |
| Subtotal (95% CI) | | 668 | | 2011 | 100.0% | 1.03 [0.84, 1.26] | |
| Total events | 104 | | 312 | | | | |
| Heterogeneity: Tau² = 0.00; Chi² = 0.78, df = 2 (P = 0.68); I² = 0% | | | | | | | |
| Test for overall effect: Z = 0.26 (P = 0.80) | | | | | | | |
| 2.8.3 All-cause graft failure at follow up (median 3.7 years) | | | | | | | |
| Hall 2019 | 148 | 585 | 475 | 1845 | 100.0% | 0.98 [0.84, 1.15] | 2019 |
| Subtotal (95% CI) | | 585 | | 1845 | 100.0% | 0.98 [0.84, 1.15] | |
| Total events | 148 | | 475 | | | | |
| Heterogeneity: Not applicable | | | | | | | |
| Test for overall effect: Z = 0.21 (P = 0.83) | | | | | | | |

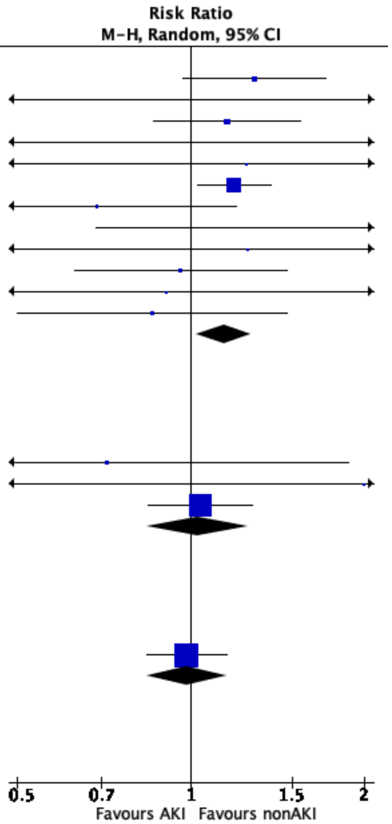


Test for subgroup differences: Chi² = 2.55, df = 2 (P = 0.28), I² = 21.4%

<u>Mortality</u>

| Study or Subgroup | AKI Events | AKI Total | nonAKI Events | nonAKI Total | Weight | Risk Ratio M-H, Random, 95% CI | Year |
|---|---|---|---|---|---|---|---|
| **2.15.1 Mortality 1 year** | | | | | | | |
| Klein 2013 | 42 | 485 | 75 | 1033 | 28.8% | 1.19 [0.83, 1.71] | 2013 |
| Torabi 2019 | 3 | 122 | 14 | 193 | 7.0% | 0.34 [0.10, 1.16] | 2019 |
| van der Windt 2020 | 2 | 96 | 15 | 435 | 5.2% | 0.60 [0.14, 2.60] | 2020 |
| Subtotal (95% CI) | | 703 | | 1661 | 41.0% | 0.75 [0.33, 1.71] | |
| Total events | 47 | | 104 | | | | |
| Heterogeneity: $Tau^2 = 0.30$; $Chi^2 = 4.36$, df = 2 (P = 0.11); $I^2 = 54\%$ | | | | | | | |
| Test for overall effect: Z = 0.69 (P = 0.49) | | | | | | | |
| **2.15.2 Mortatliy follow up to 2 years** | | | | | | | |
| Jung 2016 | 0 | 12 | 1 | 15 | 1.3% | 0.41 [0.02, 9.25] | 2016 |
| Wiwattanathum 2016 | 3 | 51 | 5 | 60 | 5.7% | 0.71 [0.18, 2.81] | 2016 |
| Subtotal (95% CI) | | 63 | | 75 | 7.0% | 0.65 [0.18, 2.28] | |
| Total events | 3 | | 6 | | | | |
| Heterogeneity: $Tau^2 = 0.00$; $Chi^2 = 0.10$, df = 1 (P = 0.75); $I^2 = 0\%$ | | | | | | | |
| Test for overall effect: Z = 0.68 (P = 0.50) | | | | | | | |
| **2.15.3 Mortatity 3 years** | | | | | | | |
| Kumar 2006 | 5 | 55 | 10 | 110 | 9.3% | 1.00 [0.36, 2.78] | 2006 |
| Subtotal (95% CI) | | 55 | | 110 | 9.3% | 1.00 [0.36, 2.78] | |
| Total events | 5 | | 10 | | | | |
| Heterogeneity: Not applicable | | | | | | | |
| Test for overall effect: Z = 0.00 (P = 1.00) | | | | | | | |
| **2.15.4 Mortality median follow up 4 years** | | | | | | | |
| Yu 2018 | 10 | 275 | 12 | 138 | 13.0% | 0.42 [0.19, 0.94] | 2018 |
| Park 2021 | 17 | 387 | 16 | 270 | 16.9% | 0.74 [0.38, 1.44] | 2021 |
| Subtotal (95% CI) | | 662 | | 408 | 29.9% | 0.59 [0.34, 1.02] | |
| Total events | 27 | | 28 | | | | |
| Heterogeneity: $Tau^2 = 0.02$; $Chi^2 = 1.14$, df = 1 (P = 0.29); $I^2 = 12\%$ | | | | | | | |
| Test for overall effect: Z = 1.90 (P = 0.06) | | | | | | | |
| **2.15.5 Mortality follow up up to 5 years** | | | | | | | |
| Farney 2013 | 2 | 84 | 17 | 283 | 5.3% | 0.40 [0.09, 1.68] | 2013 |
| Ko 2018 | 3 | 90 | 2 | 112 | 3.7% | 1.87 [0.32, 10.93] | 2018 |
| Subtotal (95% CI) | | 174 | | 395 | 9.0% | 0.79 [0.17, 3.61] | |
| Total events | 5 | | 19 | | | | |
| Heterogeneity: $Tau^2 = 0.54$; $Chi^2 = 1.80$, df = 1 (P = 0.18); $I^2 = 44\%$ | | | | | | | |
| Test for overall effect: Z = 0.30 (P = 0.76) | | | | | | | |
| **2.15.6 Mortality mean follow up 54-69 months** | | | | | | | |
| Kwon 2019 | 2 | 30 | 3 | 151 | 3.8% | 3.36 [0.59, 19.23] | 2019 |
| Subtotal (95% CI) | | 30 | | 151 | 3.8% | 3.36 [0.59, 19.23] | |
| Total events | 2 | | 3 | | | | |
| Heterogeneity: Not applicable | | | | | | | |
| Test for overall effect: Z = 1.36 (P = 0.17) | | | | | | | |
| **Total (95% CI)** | | 1687 | | 2800 | 100.0% | 0.80 [0.56, 1.14] | |
| Total events | 69 | | 170 | | | | |
| Heterogeneity: $Tau^2 = 0.08$; $Chi^2 = 13.34$, df = 10 (P = 0.21); $I^2 = 25\%$ | | | | | | | |
| Test for overall effect: Z = 1.25 (P = 0.21) | | | | | | | |
| Test for subgroup differences: $Chi^2 = 3.96$, df = 5 (P = 0.56), $I^2 = 0\%$ | | | | | | | |

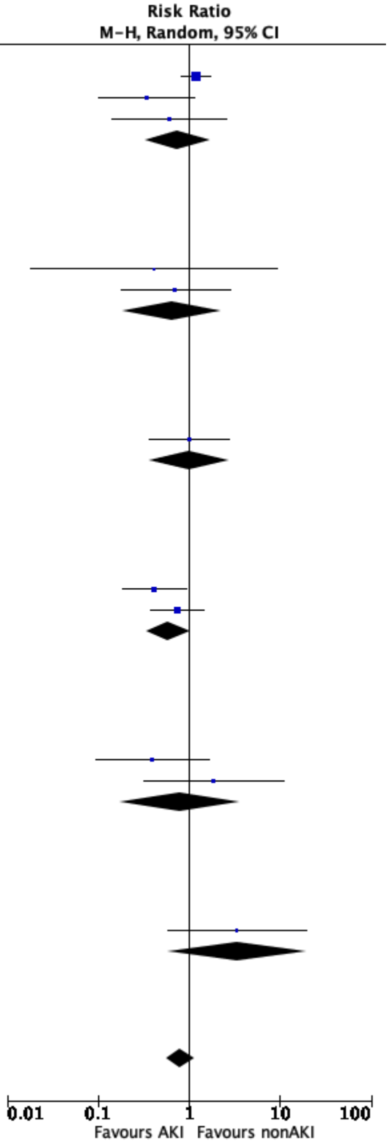

# Delayed Graft Function

| Study or Subgroup | AKI Events | AKI Total | nonAKI Events | nonAKI Total | Weight | Risk Ratio M-H, Random, 95% CI | Year |
|---|---|---|---|---|---|---|---|
| Ugarte 2005 | 38 | 65 | 80 | 197 | 4.4% | 1.44 [1.10, 1.88] | 2005 |
| Kumar 2006 | 48 | 55 | 59 | 110 | 5.0% | 1.63 [1.33, 1.99] | 2006 |
| Sohrabi 2007 | 2 | 9 | 19 | 40 | 0.6% | 0.47 [0.13, 1.66] | 2007 |
| Zuckerman 2009 | 8 | 25 | 19 | 86 | 1.6% | 1.45 [0.72, 2.90] | 2009 |
| Rodrigo 2010 | 17 | 33 | 83 | 279 | 3.4% | 1.73 [1.19, 2.52] | 2010 |
| Kolonko 2011 | 4 | 10 | 17 | 51 | 1.2% | 1.20 [0.51, 2.81] | 2011 |
| Farney 2013 | 34 | 84 | 77 | 283 | 3.8% | 1.49 [1.08, 2.05] | 2013 |
| Klein 2013 | 337 | 485 | 533 | 1033 | 5.9% | 1.35 [1.24, 1.46] | 2013 |
| Jung 2013 | 7 | 36 | 0 | 18 | 0.1% | 7.70 [0.46, 127.78] | 2013 |
| Yu 2014 | 14 | 28 | 18 | 56 | 2.3% | 1.56 [0.91, 2.65] | 2014 |
| Jacobi 2014 | 21 | 63 | 79 | 319 | 3.2% | 1.35 [0.90, 2.00] | 2014 |
| Yuan 2014 | 8 | 29 | 10 | 60 | 1.2% | 1.66 [0.73, 3.75] | 2014 |
| Lee 2014 | 18 | 43 | 14 | 113 | 2.0% | 3.38 [1.85, 6.18] | 2014 |
| Hellman 2014 | 109 | 162 | 164 | 609 | 5.3% | 2.50 [2.11, 2.96] | 2014 |
| Hall 2015 | 257 | 622 | 543 | 1945 | 5.7% | 1.48 [1.32, 1.66] | 2015 |
| Molina 2015 | 40 | 59 | 21 | 59 | 3.3% | 1.90 [1.30, 2.80] | 2015 |
| Ali 2015 | 47 | 114 | 43 | 170 | 3.7% | 1.63 [1.16, 2.29] | 2015 |
| Jung 2016 | 2 | 12 | 3 | 15 | 0.4% | 0.83 [0.16, 4.21] | 2016 |
| Wiwattanathum 2016 | 29 | 51 | 31 | 60 | 3.7% | 1.10 [0.78, 1.55] | 2016 |
| Boffa 2017 | 541 | 1869 | 2618 | 9350 | 6.0% | 1.03 [0.96, 1.12] | 2017 |
| Ko 2018 | 22 | 90 | 8 | 112 | 1.4% | 3.42 [1.60, 7.32] | 2018 |
| Gwon 2018 | 11 | 64 | 1 | 37 | 0.2% | 6.36 [0.85, 47.31] | 2018 |
| Bauer 2018 | 104 | 303 | 97 | 339 | 4.7% | 1.20 [0.95, 1.51] | 2018 |
| Yu 2018 | 35 | 275 | 10 | 138 | 1.7% | 1.76 [0.90, 3.44] | 2018 |
| Kwon 2019 | 12 | 30 | 11 | 151 | 1.5% | 5.49 [2.68, 11.26] | 2019 |
| Jiang 2019 | 21 | 60 | 26 | 135 | 2.6% | 1.82 [1.12, 2.96] | 2019 |
| Cima 2019 | 12 | 64 | 33 | 271 | 2.0% | 1.54 [0.84, 2.81] | 2019 |
| Domagala 2019 | 37 | 66 | 37 | 106 | 3.7% | 1.61 [1.15, 2.25] | 2019 |
| Torabi 2019 | 81 | 122 | 104 | 193 | 5.2% | 1.23 [1.03, 1.48] | 2019 |
| Schutte-Nutgen 2019 | 35 | 107 | 20 | 107 | 2.6% | 1.75 [1.08, 2.83] | 2019 |
| Liu 2020 | 3643 | 12513 | 2779 | 12810 | 6.1% | 1.34 [1.29, 1.40] | 2020 |
| van der Windt 2020 | 14 | 53 | 66 | 280 | 2.5% | 1.12 [0.68, 1.84] | 2020 |
| Park 2021 | 93 | 287 | 26 | 270 | 3.2% | 3.37 [2.25, 5.03] | 2021 |
| **Total (95% CI)** | | 17888 | | 29802 | 100.0% | 1.57 [1.42, 1.74] | |
| Total events | 5701 | | 7649 | | | | |

Heterogeneity: $Tau^2 = 0.04$; $Chi^2 = 172.97$, $df = 32$ ($P < 0.00001$); $I^2 = 82\%$

Test for overall effect: $Z = 8.71$ ($P < 0.00001$)

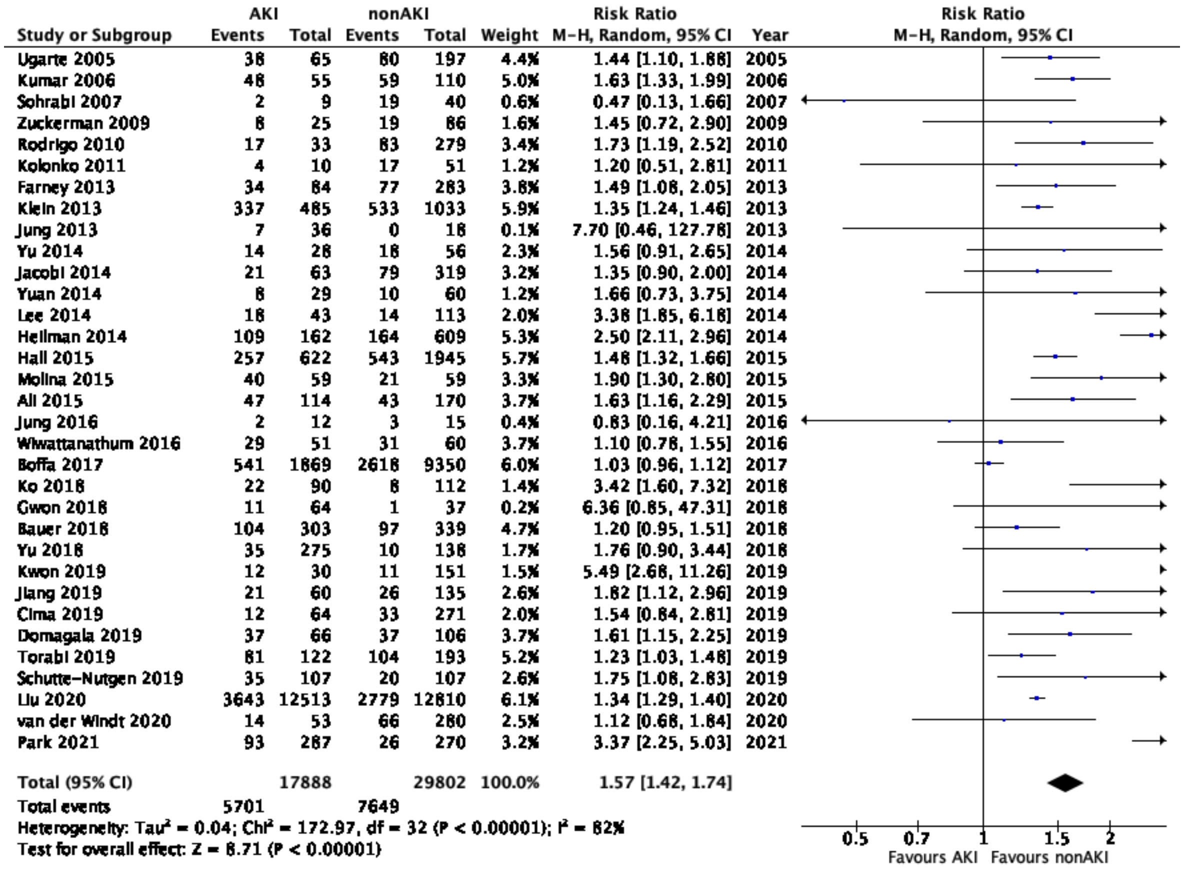

## Acute Rejection

| | AKI | | nonAKI | | | Risk Ratio | |
|---|---|---|---|---|---|---|---|
| Study or Subgroup | Events | Total | Events | Total | Weight | M-H, Random, 95% CI | Year |
| Ugarte 2005 | 25 | 65 | 71 | 197 | 5.4% | 1.07 [0.74, 1.53] | 2005 |
| Kumar 2006 | 6 | 55 | 14 | 110 | 0.9% | 0.86 [0.35, 2.11] | 2006 |
| Sohrabi 2007 | 1 | 9 | 11 | 40 | 0.2% | 0.40 [0.06, 2.74] | 2007 |
| Zuckerman 2009 | 3 | 25 | 11 | 86 | 0.5% | 0.94 [0.28, 3.10] | 2009 |
| Rodrigo 2010 | 9 | 33 | 87 | 279 | 2.1% | 0.87 [0.49, 1.57] | 2010 |
| Klein 2013 | 162 | 485 | 323 | 1033 | 29.2% | 1.07 [0.92, 1.25] | 2013 |
| Jacobi 2014 | 12 | 51 | 67 | 274 | 2.4% | 0.96 [0.56, 1.65] | 2014 |
| Yuan 2014 | 3 | 29 | 5 | 60 | 0.4% | 1.24 [0.32, 4.84] | 2014 |
| Yu 2014 | 6 | 28 | 12 | 56 | 0.9% | 1.00 [0.42, 2.38] | 2014 |
| Hellman 2015 | 19 | 162 | 115 | 609 | 3.4% | 0.62 [0.39, 0.98] | 2015 |
| Molina 2015 | 14 | 59 | 14 | 59 | 1.7% | 1.00 [0.52, 1.91] | 2015 |
| Benck 2015 | 5 | 33 | 6 | 65 | 0.6% | 1.64 [0.54, 4.98] | 2015 |
| Ali 2015 | 22 | 114 | 24 | 170 | 2.5% | 1.37 [0.81, 2.32] | 2015 |
| Wiwattanathum 2016 | 10 | 51 | 11 | 60 | 1.2% | 1.07 [0.49, 2.31] | 2016 |
| Jung 2016 | 0 | 12 | 3 | 15 | 0.1% | 0.18 [0.01, 3.11] | 2016 |
| Yu 2018 | 146 | 275 | 76 | 138 | 19.9% | 0.96 [0.80, 1.16] | 2018 |
| Ko 2018 | 31 | 90 | 44 | 112 | 5.2% | 0.88 [0.61, 1.26] | 2018 |
| Bauer 2018 | 86 | 303 | 89 | 339 | 11.0% | 1.08 [0.84, 1.39] | 2018 |
| Jiang 2019 | 10 | 60 | 22 | 135 | 1.5% | 1.02 [0.52, 2.02] | 2019 |
| Torabi 2019 | 5 | 122 | 9 | 193 | 0.6% | 0.88 [0.30, 2.56] | 2019 |
| Kwon 2019 | 4 | 30 | 35 | 151 | 0.8% | 0.58 [0.22, 1.50] | 2019 |
| Domagala 2019 | 7 | 66 | 13 | 106 | 0.9% | 0.86 [0.36, 2.06] | 2019 |
| van der Windt 2020 | 20 | 53 | 103 | 280 | 4.9% | 1.03 [0.70, 1.50] | 2020 |
| Park 2021 | 55 | 287 | 28 | 270 | 3.9% | 1.85 [1.21, 2.82] | 2021 |
| Total (95% CI) | | 2497 | | 4837 | 100.0% | 1.02 [0.94, 1.11] | |
| Total events | 661 | | 1193 | | | | |

Heterogeneity: $Tau^2 = 0.00$; $Chi^2 = 20.15$, df = 23 (P = 0.63); $I^2 = 0\%$

Test for overall effect: Z = 0.56 (P = 0.58)

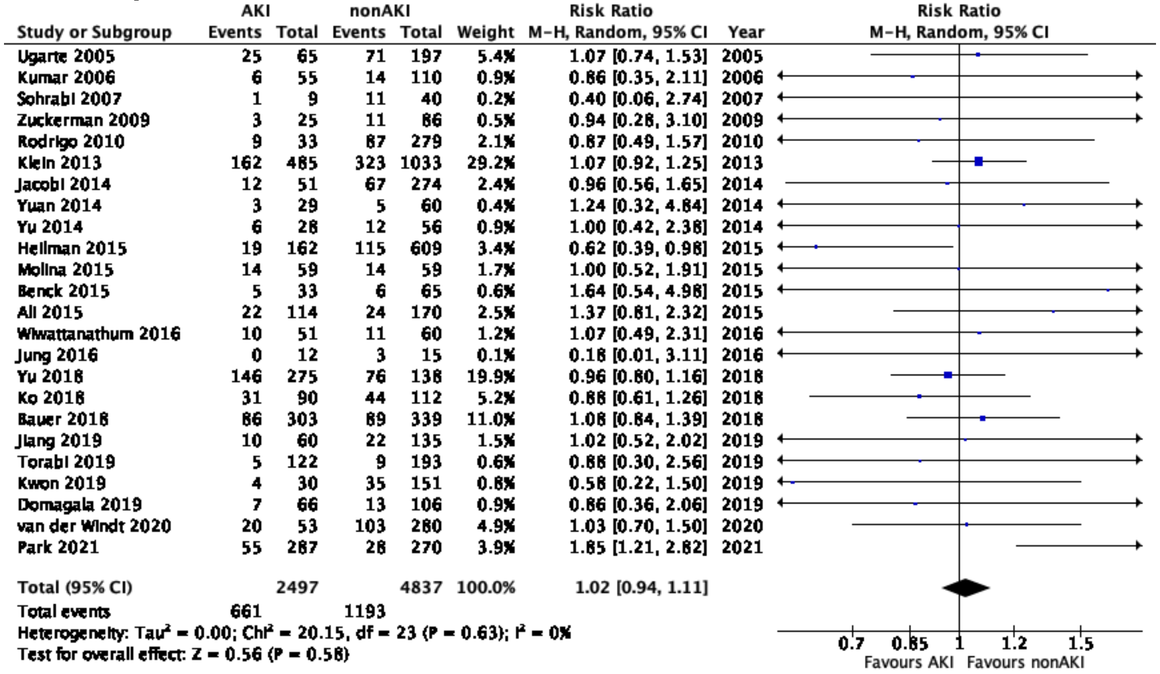

# 5. Evidence Summary: ECD with AKI and Non ECD (SCD) with AKI for renal transplant

| Certainty assessment | | | | | | | № of patients | | Effect | | Certainty | Importance |
|---|---|---|---|---|---|---|---|---|---|---|---|---|
| № of studies | Study design | Risk of bias | Inconsistency | Indirectness | Imprecision | Other considerations | Standard criteria kidney with AKI | Extended criteria kidney with AKI | Relative (95% CI) | Absolute (95% CI) | | |
| **Mortality AKI-SCD vs AKI-ECD** | | | | | | | | | | | | |
| 3 | non-randomized studies | serious | serious[a] | not serious | very serious[b] | none[c] | 41/539 (7.6%) | 15/238 (6.3%) | **RR 1.1** (0.6 to 2.0) | **6 more per 1,000** (from 25 fewer to 63 more) | ⨁◯◯◯ Very low | CRITICAL |
| **Graft survival AKI-SCD vs AKI-ECD** | | | | | | | | | | | | |
| 3 | non-randomized studies | serious | serious | not serious | serious[d] | none[c] | 365/444 (82.2%) | 140/182 (76.9%) | **RR 1.1** (1.0 to 1.2) | **77 more per 1,000** (from 0 fewer to 154 more) | ⨁◯◯◯ Very low | CRITICAL |
| **Delayed graft function AKI-SCD vs AKI-ECD** | | | | | | | | | | | | |
| 6 | non-randomized studies | serious[e] | serious[a] | not serious | very serious[b] | none[c] | 391/755 (51.8%) | 138/336 (41.1%) | **RR 0.99** (0.88 to 1.11) | **4 fewer per 1,000** (from 49 fewer to 45 more) | ⨁◯◯◯ Very low | IMPORTANT |
| **Acute rejection rate AKI-SCD vs AKI-ECD** | | | | | | | | | | | | |
| 5 | non-randomized studies | serious | serious[a] | not serious | very serious[b] | none[c] | 172/599 (28.7%) | 52/211 (24.6%) | **RR 1.19** (0.89 to 1.59) | **47 more per 1,000** (from 27 fewer to 145 more) | ⨁◯◯◯ Very low | IMPORTANT |
| **eGFR at one year AKI-SCD vs AKI-ECD** | | | | | | | | | | | | |
| 1 | non-randomized studies | serious | not serious | not serious | serious[d] | none | 19 | 19 | - | MD **9.5 higher** (0.98 higher to 18.02 higher) | ⨁◯◯◯ Very low | IMPORTANT |
| **Infection rate AKI-SCD vs AKI-ECD** | | | | | | | | | | | | |
| 1 | non-randomized studies | serious | not serious | not serious | serious[d] | none | 17/139 (12.2%) | 5/23 (21.7%) | **RR 0.56** (0.23 to 1.38) | **96 fewer per 1,000** (from 167 fewer to 83 more) | ⨁◯◯◯ Very low | IMPORTANT |

**CI:** confidence interval; **MD:** mean difference; **RR:** risk ratio

**Explanations**

a. Rated down as point estimates vary across studies
b. Rated down for imprecision as low number of events, and confidence intervals overlap.
c. Assessment of publication bias could not be done as less then 9 studies identified.
d. Rated down for few number of events
e. Observational studies with possible duplicate populations.

# Forest Plot: ECD and Non ECD with AKI

## Mortality

| Study or Subgroup | AKI-SCD Events | AKI-SCD Total | AKI-ECD Events | AKI-ECD Total | Weight | Risk Ratio M-H, Random, 95% CI | Year |
|---|---|---|---|---|---|---|---|
| 1.31.1 Mortality one year | | | | | | | |
| Klein 2013 | 34 | 369 | 8 | 116 | 65.4% | 1.34 [0.64, 2.80] | 2013 |
| Subtotal (95% CI) | | 369 | | 116 | 65.4% | 1.34 [0.64, 2.80] | |
| Total events | 34 | | 8 | | | | |
| Heterogeneity: Not applicable | | | | | | | |
| Test for overall effect: Z = 0.77 (P = 0.44) | | | | | | | |
| 1.31.2 Mortality 2 years | | | | | | | |
| Jung 2016 | 0 | 6 | 0 | 6 | | Not estimable | 2016 |
| Subtotal (95% CI) | | 6 | | 6 | | Not estimable | |
| Total events | 0 | | 0 | | | | |
| Heterogeneity: Not applicable | | | | | | | |
| Test for overall effect: Not applicable | | | | | | | |
| 1.31.3 Mortality 4 years | | | | | | | |
| Park 2019 | 7 | 154 | 7 | 116 | 34.6% | 0.75 [0.27, 2.09] | 2019 |
| Subtotal (95% CI) | | 154 | | 116 | 34.6% | 0.75 [0.27, 2.09] | |
| Total events | 7 | | 7 | | | | |
| Heterogeneity: Not applicable | | | | | | | |
| Test for overall effect: Z = 0.54 (P = 0.59) | | | | | | | |
| Total (95% CI) | | 529 | | 238 | 100.0% | 1.10 [0.60, 2.00] | |
| Total events | 41 | | 15 | | | | |
| Heterogeneity: $Tau^2 = 0.00$; $Chi^2 = 0.79$, df = 1 (P = 0.37); $I^2 = 0\%$ | | | | | | | |
| Test for overall effect: Z = 0.30 (P = 0.76) | | | | | | | |
| Test for subgroup differences: $Chi^2 = 0.79$, df = 1 (P = 0.37), $I^2 = 0\%$ | | | | | | | |

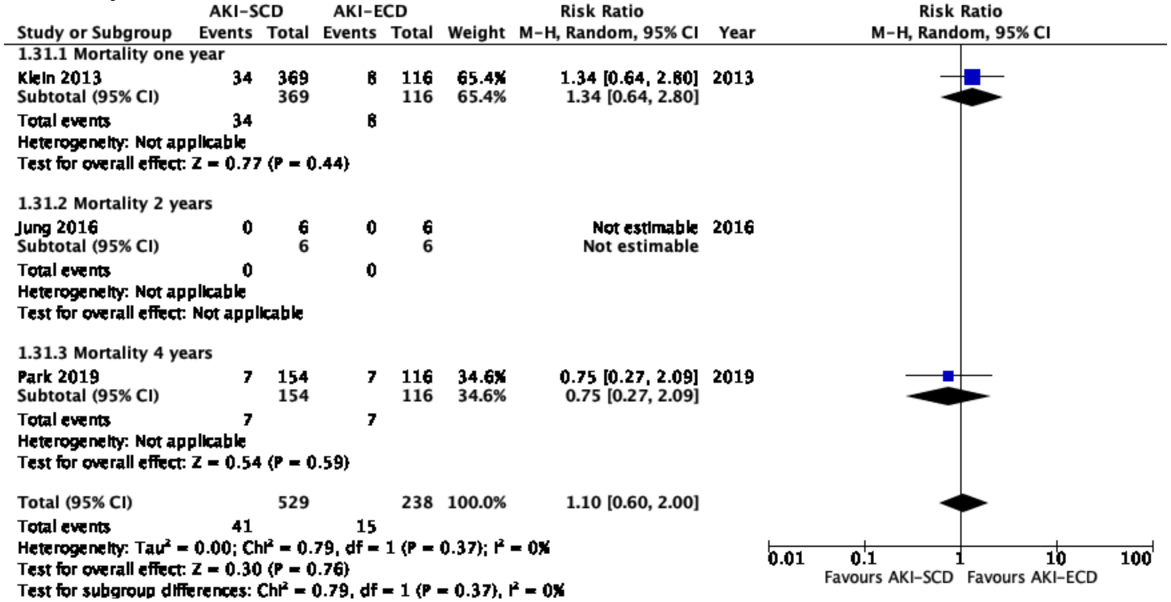


## Acute Rejection

| Study or Subgroup | AKI-SCD Events | AKI-SCD Total | AKI-ECD Events | AKI-ECD Total | Weight | Risk Ratio M-H, Random, 95% CI | Year |
|---|---|---|---|---|---|---|---|
| Klein 2013 | 129 | 369 | 33 | 116 | 66.6% | 1.23 [0.89, 1.69] | 2013 |
| Jacobi 2014 | 7 | 23 | 5 | 28 | 8.1% | 1.70 [0.62, 4.66] | 2014 |
| Heilman 2015 | 19 | 139 | 0 | 23 | 1.1% | 6.69 [0.42, 107.07] | 2015 |
| Jung 2016 | 0 | 6 | 0 | 6 | | Not estimable | 2016 |
| Ko 2018 | 17 | 52 | 14 | 38 | 24.1% | 0.89 [0.50, 1.57] | 2018 |
| Total (95% CI) | | 589 | | 211 | 100.0% | 1.19 [0.89, 1.59] | |
| Total events | 172 | | 52 | | | | |
| Heterogeneity: $Tau^2 = 0.01$; $Chi^2 = 3.15$, df = 3 (P = 0.37); $I^2 = 5\%$ | | | | | | | |
| Test for overall effect: Z = 1.17 (P = 0.24) | | | | | | | |

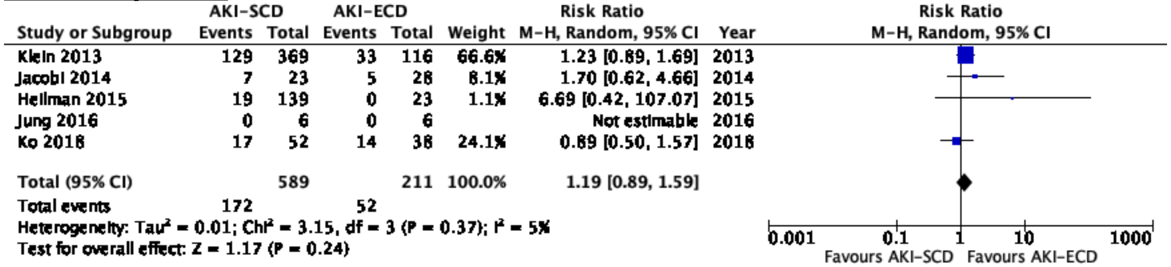

## Delayed Graft Function

| Study or Subgroup | AKI-SCD Events | AKI-SCD Total | AKI-ECD Events | AKI-ECD Total | Weight | Risk Ratio M-H, Random, 95% CI | Year |
|---|---|---|---|---|---|---|---|
| Klein 2013 | 258 | 368 | 79 | 116 | 70.9% | 1.03 [0.89, 1.19] | 2013 |
| Jacobi 2014 | 9 | 26 | 12 | 37 | 2.9% | 1.07 [0.53, 2.16] | 2014 |
| Heilman 2014 | 92 | 139 | 17 | 23 | 19.4% | 0.90 [0.68, 1.17] | 2014 |
| Jung 2016 | 0 | 6 | 2 | 6 | 0.2% | 0.20 [0.01, 3.46] | 2016 |
| Ko 2018 | 13 | 52 | 9 | 38 | 2.6% | 1.06 [0.50, 2.21] | 2018 |
| Park 2019 | 19 | 154 | 19 | 116 | 4.1% | 0.75 [0.42, 1.36] | 2019 |
| Total (95% CI) | | 745 | | 336 | 100.0% | 0.99 [0.88, 1.11] | |
| Total events | 391 | | 138 | | | | |

Heterogeneity: $Tau^2 = 0.00$; $Chi^2 = 3.02$, df = 5 (P = 0.70); $I^2 = 0\%$
Test for overall effect: Z = 0.20 (P = 0.84)

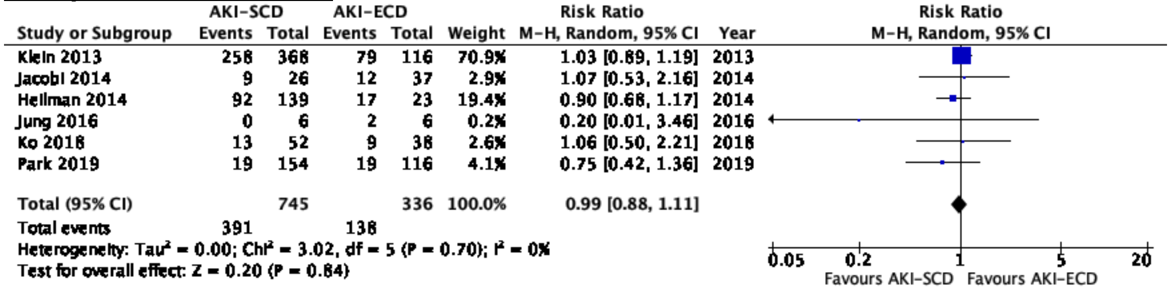


## Overall Graft Survival

| Study or Subgroup | AKI-SCD Events | AKI-SCD Total | AKI-ECD Events | AKI-ECD Total | Weight | Risk Ratio M-H, Random, 95% CI | Year |
|---|---|---|---|---|---|---|---|
| 1.6.1 1 year Graft survival | | | | | | | |
| Klein 2013 | 296 | 369 | 88 | 116 | 60.9% | 1.06 [0.94, 1.19] | 2013 |
| Jacobi 2014 | 21 | 23 | 22 | 28 | 15.0% | 1.16 [0.92, 1.46] | 2014 |
| Subtotal (95% CI) | | 392 | | 144 | 75.9% | 1.08 [0.97, 1.19] | |
| Total events | 317 | | 110 | | | | |
| Heterogeneity: $Tau^2 = 0.00$; $Chi^2 = 0.53$, df = 1 (P = 0.47); $I^2 = 0\%$ | | | | | | | |
| Test for overall effect: Z = 1.42 (P = 0.15) | | | | | | | |
| 1.6.2 Mean follow up 40 months | | | | | | | |
| Ko 2018 | 48 | 52 | 30 | 38 | 24.1% | 1.17 [0.97, 1.40] | 2018 |
| Subtotal (95% CI) | | 52 | | 38 | 24.1% | 1.17 [0.97, 1.40] | |
| Total events | 48 | | 30 | | | | |
| Heterogeneity: Not applicable | | | | | | | |
| Test for overall effect: Z = 1.68 (P = 0.09) | | | | | | | |
| Total (95% CI) | | 444 | | 182 | 100.0% | 1.10 [1.00, 1.20] | |
| Total events | 365 | | 140 | | | | |

Heterogeneity: $Tau^2 = 0.00$; $Chi^2 = 1.14$, df = 2 (P = 0.56); $I^2 = 0\%$
Test for overall effect: Z = 2.07 (P = 0.04)
Test for subgroup differences: $Chi^2 = 0.59$, df = 1 (P = 0.44), $I^2 = 0\%$

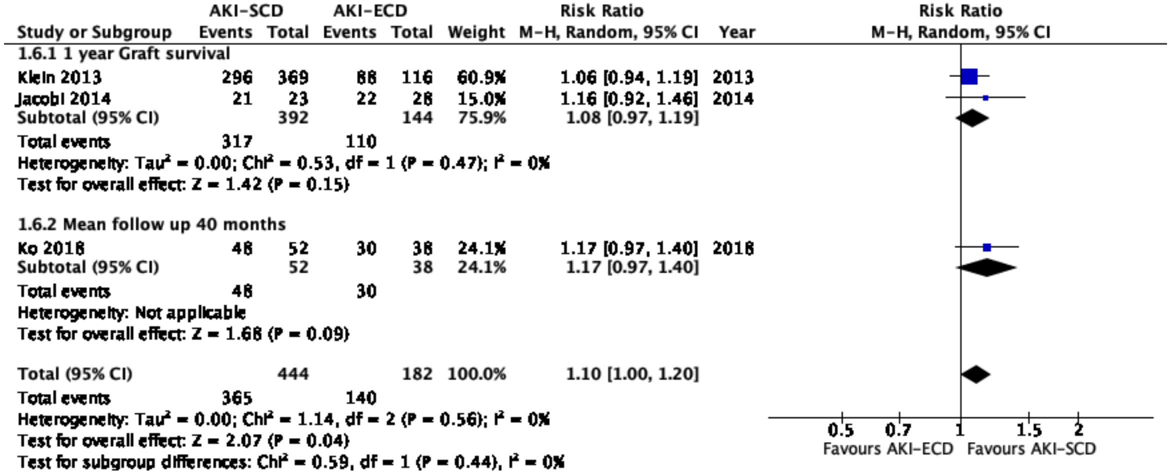

# 6. **Evidence Summary:** DCD donors compared to NDD donors for renal transplant

| Certainty assessment | | | | | | | № of patients | | Effect | | Certainty | Importance |
|---|---|---|---|---|---|---|---|---|---|---|---|---|
| № of studies | Study design | Risk of bias | Inconsistency | Indirectness | Imprecision | Other considerations | DCD donors | NDD donors | Relative (95% CI) | Absolute (95% CI) | | |
| **Mortality** | | | | | | | | | | | | |
| 9 | non-randomized studies | serious[a] | serious[b] | not serious | not serious | none | 2414/23274 (10.4%) | 2735/36264 (7.5%) | **RR 1.33** (1.15 to 1.54) | **25 more per 1,000** (from 11 more to 41 more) | ⨁◯◯◯ Very low | CRITICAL |
| **Graft Loss** | | | | | | | | | | | | |
| 7 | non-randomized studies | serious[a] | not serious | not serious | not serious | none | 886/9151 (9.7%) | 3677/17454 (21.1%) | **RR 1.08** (1.00 to 1.17) | **17 more per 1,000** (from 0 fewer to 36 more) | ⨁◯◯◯ Very low | CRITICAL |
| **Death Censored-Graft Loss** | | | | | | | | | | | | |
| 2 | non-randomized studies | serious[a] | not serious | not serious | serious[c] | none | 1515/20039 (7.6%) | 2783/30816 (9.0%) | **RR 1.04** (0.92 to 1.17) | **4 more per 1,000** (from 7 fewer to 15 more) | ⨁◯◯◯ Very low | CRITICAL |
| **Acute Rejection** | | | | | | | | | | | | |
| 7 | non-randomized studies | serious[a] | not serious | not serious | serious[c] | none | 2939/7471 (39.3%) | 7669/29040 (26.4%) | **RR 1.62** (0.77 to 3.42) | **164 more per 1,000** (from 61 fewer to 639 more) | ⨁◯◯◯ Very low | IMPORTANT |
| **Delayed graft function** | | | | | | | | | | | | |
| 10 | non-randomized studies | serious[a] | not serious | not serious | not serious | none | 11229/27526 (40.8%) | 29753/137459 (21.6%) | **RR 1.89** (1.80 to 1.99) | **193 more per 1,000** (from 173 more to 214 more) | ⨁◯◯◯ Very low | IMPORTANT |

**CI:** confidence interval; **RR:** risk ratio

**Explanations**

a. rated down for risk of bias given the possibility of double counting of participants in registry studies
b. rated down for heterogeneity
c. rated down due to wide confidence intervals that include the line of no effect

## Forest Plots: DCD Versus NDD

### All-Cause Mortality

| Study or Subgroup | DCD events Events | DCD events Total | NDD events Events | NDD events Total | Weight | Risk Ratio M-H, Random, 95% CI |
|---|---|---|---|---|---|---|
| **1.1.1 Mortality at 1 year** | | | | | | |
| Aida 2022 | 76 | 2239 | 10 | 684 | 3.6% | 2.32 [1.21, 4.46] |
| Chen 2017 | 2 | 258 | 1 | 59 | 0.4% | 0.46 [0.04, 4.96] |
| Perez-Saez 2021 | 36 | 277 | 52 | 575 | 6.6% | 1.44 [0.96, 2.14] |
| Sanchez-Fructuoso 2019 | 29 | 774 | 6 | 366 | 2.3% | 2.29 [0.96, 5.46] |
| Savoye 2021 | 63 | 499 | 445 | 6185 | 9.6% | 1.75 [1.37, 2.25] |
| Snoeijs 2010 (A) | 552 | 570 | 211 | 230 | 13.2% | 1.06 [1.01, 1.10] |
| Trotter 2018 | 248 | 5899 | 446 | 12741 | 11.6% | 1.20 [1.03, 1.40] |
| Yuan 2014 | 2 | 101 | 0 | 50 | 0.2% | 2.50 [0.12, 51.11] |
| Subtotal (95% CI) | | 10617 | | 20890 | 47.5% | 1.46 [1.05, 2.03] |
| Total events | 1008 | | 1171 | | | |
| Heterogeneity: $Tau^2 = 0.13$; $Chi^2 = 71.62$, df = 7 ($P < 0.00001$); $I^2 = 90\%$ | | | | | | |
| Test for overall effect: Z = 2.27 (P = 0.02) | | | | | | |
| **1.1.2 Mortality at 3 years** | | | | | | |
| Aida 2022 | 134 | 2239 | 30 | 684 | 6.9% | 1.36 [0.93, 2.01] |
| Chen 2017 | 9 | 258 | 3 | 59 | 1.2% | 0.69 [0.19, 2.46] |
| Subtotal (95% CI) | | 2497 | | 743 | 8.0% | 1.28 [0.86, 1.90] |
| Total events | 143 | | 33 | | | |
| Heterogeneity: $Tau^2 = 0.01$; $Chi^2 = 1.02$, df = 1 (P = 0.31); $I^2 = 2\%$ | | | | | | |
| Test for overall effect: Z = 1.23 (P = 0.22) | | | | | | |
| **1.1.3 Mortality at 5 years** | | | | | | |
| Aida 2022 | 215 | 2239 | 49 | 684 | 8.5% | 1.34 [0.99, 1.81] |
| Molina 2019 | 16 | 237 | 10 | 237 | 2.8% | 1.60 [0.74, 3.45] |
| Sanchez-Fructuoso 2019 | 83 | 774 | 23 | 366 | 5.9% | 1.71 [1.09, 2.66] |
| Trotter 2018 | 779 | 5899 | 1363 | 12741 | 12.8% | 1.23 [1.14, 1.34] |
| Subtotal (95% CI) | | 9149 | | 14028 | 30.0% | 1.26 [1.16, 1.36] |
| Total events | 1093 | | 1445 | | | |
| Heterogeneity: $Tau^2 = 0.00$; $Chi^2 = 2.56$, df = 3 (P = 0.46); $I^2 = 0\%$ | | | | | | |
| Test for overall effect: Z = 5.76 ($P < 0.00001$) | | | | | | |
| **1.1.4 Mortality at 10 years** | | | | | | |
| Molina 2019 | 32 | 237 | 29 | 237 | 5.6% | 1.10 [0.69, 1.76] |
| Sanchez-Fructuoso 2019 | 138 | 774 | 57 | 366 | 8.9% | 1.14 [0.86, 1.52] |
| Subtotal (95% CI) | | 1011 | | 603 | 14.4% | 1.13 [0.89, 1.44] |
| Total events | 170 | | 86 | | | |
| Heterogeneity: $Tau^2 = 0.00$; $Chi^2 = 0.02$, df = 1 (P = 0.90); $I^2 = 0\%$ | | | | | | |
| Test for overall effect: Z = 1.02 (P = 0.31) | | | | | | |
| **Total (95% CI)** | | 23274 | | 36264 | 100.0% | 1.33 [1.15, 1.54] |
| Total events | 2414 | | 2735 | | | |
| Heterogeneity: $Tau^2 = 0.04$; $Chi^2 = 80.00$, df = 15 ($P < 0.00001$); $I^2 = 81\%$ | | | | | | |
| Test for overall effect: Z = 3.86 (P = 0.0001) | | | | | | |
| Test for subgroup differences: $Chi^2 = 1.52$, df = 3 (P = 0.68), $I^2 = 0\%$ | | | | | | |

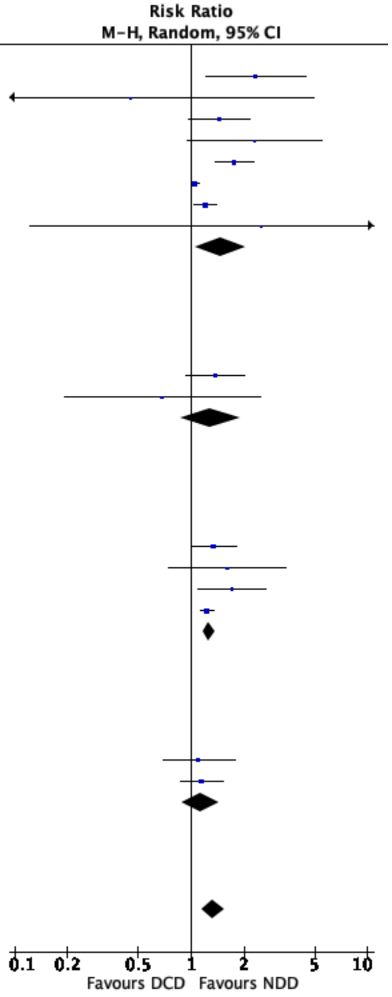

# Graft Loss

| Study or Subgroup | DCD events Events | DCD events Total | NDD events Events | NDD events Total | Weight | Risk Ratio M-H, Random, 95% CI | Risk Ratio M-H, Random, 95% CI |
|---|---|---|---|---|---|---|---|
| 1.2.1 Overall | | | | | | | |
| Molina 2019 | 1 | 19 | 5 | 67 | 0.1% | 0.71 [0.09, 5.68] | |
| Snoeijs 2010 (A) | 119 | 297 | 232 | 594 | 21.4% | 1.03 [0.86, 1.22] | |
| Subtotal (95% CI) | | 316 | | 661 | 21.6% | 1.02 [0.86, 1.21] | |
| Total events | 120 | | 237 | | | | |
| Heterogeneity: $Tau^2 = 0.00$; $Chi^2 = 0.12$, df = 1 (P = 0.73); $I^2 = 0\%$ | | | | | | | |
| Test for overall effect: Z = 0.26 (P = 0.79) | | | | | | | |
| 1.2.2 Graft loss at 1 year | | | | | | | |
| Aida 2022 | 38 | 2239 | 12 | 684 | 1.5% | 0.97 [0.51, 1.84] | |
| Chen 2017 | 10 | 258 | 1 | 59 | 0.2% | 2.29 [0.30, 17.52] | |
| Savoye 2021 | 61 | 442 | 71 | 809 | 6.1% | 1.57 [1.14, 2.17] | |
| Yuan 2014 | 3 | 101 | 1 | 50 | 0.1% | 1.49 [0.16, 13.92] | |
| Subtotal (95% CI) | | 3040 | | 1602 | 7.9% | 1.44 [1.09, 1.91] | |
| Total events | 112 | | 85 | | | | |
| Heterogeneity: $Tau^2 = 0.00$; $Chi^2 = 1.96$, df = 3 (P = 0.58); $I^2 = 0\%$ | | | | | | | |
| Test for overall effect: Z = 2.53 (P = 0.01) | | | | | | | |
| 1.2.3 Graft loss at 3 year | | | | | | | |
| Aida 2022 | 132 | 2239 | 36 | 684 | 4.9% | 1.12 [0.78, 1.60] | |
| Chen 2017 | 22 | 258 | 3 | 59 | 0.5% | 1.68 [0.52, 5.42] | |
| Subtotal (95% CI) | | 2497 | | 743 | 5.4% | 1.16 [0.82, 1.63] | |
| Total events | 154 | | 39 | | | | |
| Heterogeneity: $Tau^2 = 0.00$; $Chi^2 = 0.42$, df = 1 (P = 0.52); $I^2 = 0\%$ | | | | | | | |
| Test for overall effect: Z = 0.85 (P = 0.40) | | | | | | | |
| 1.2.4 Graft loss at 5 year | | | | | | | |
| Aida 2022 | 230 | 2239 | 74 | 684 | 10.3% | 0.95 [0.74, 1.22] | |
| Heylen 2018 | 270 | 1059 | 3242 | 13764 | 54.9% | 1.08 [0.97, 1.20] | |
| Subtotal (95% CI) | | 3298 | | 14448 | 65.2% | 1.06 [0.96, 1.17] | |
| Total events | 500 | | 3316 | | | | |
| Heterogeneity: $Tau^2 = 0.00$; $Chi^2 = 0.91$, df = 1 (P = 0.34); $I^2 = 0\%$ | | | | | | | |
| Test for overall effect: Z = 1.16 (P = 0.24) | | | | | | | |
| Total (95% CI) | | 9151 | | 17454 | 100.0% | 1.08 [1.00, 1.17] | |
| Total events | 886 | | 3677 | | | | |
| Heterogeneity: $Tau^2 = 0.00$; $Chi^2 = 8.08$, df = 9 (P = 0.53); $I^2 = 0\%$ | | | | | | | |
| Test for overall effect: Z = 1.97 (P = 0.05) | | | | | | | |
| Test for subgroup differences: $Chi^2 = 4.67$, df = 3 (P = 0.20), $I^2 = 35.7\%$ | | | | | | | |

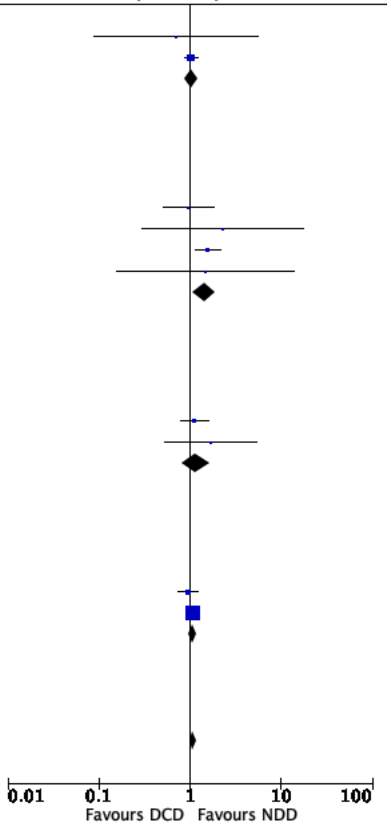

## Death-censored graft loss

| Study or Subgroup | DCD events Events | DCD events Total | NDD events Events | NDD events Total | Weight | Risk Ratio M-H, Random, 95% CI |
|---|---|---|---|---|---|---|
| 1.3.1 at 1 year | | | | | | |
| Aida 2022 | 29 | 2239 | 5 | 684 | 1.5% | 1.77 [0.69, 4.56] |
| Trotter 2018 | 426 | 6661 | 820 | 14382 | 37.4% | 1.12 [1.00, 1.26] |
| Subtotal (95% CI) | | 8900 | | 15066 | 38.9% | 1.13 [1.01, 1.26] |
| Total events | 455 | | 825 | | | |
| Heterogeneity: Tau² = 0.00; Chi² = 0.89, df = 1 (P = 0.35); I² = 0% | | | | | | |
| Test for overall effect: Z = 2.11 (P = 0.03) | | | | | | |
| 1.3.2 at 3 years | | | | | | |
| Aida 2022 | 74 | 2239 | 18 | 684 | 4.9% | 1.26 [0.76, 2.09] |
| Subtotal (95% CI) | | 2239 | | 684 | 4.9% | 1.26 [0.76, 2.09] |
| Total events | 74 | | 18 | | | |
| Heterogeneity: Not applicable | | | | | | |
| Test for overall effect: Z = 0.88 (P = 0.38) | | | | | | |
| 1.3.3 at 5 years | | | | | | |
| Aida 2022 | 107 | 2239 | 42 | 684 | 9.6% | 0.78 [0.55, 1.10] |
| Trotter 2018 | 879 | 6661 | 1898 | 14382 | 46.5% | 1.00 [0.93, 1.08] |
| Subtotal (95% CI) | | 8900 | | 15066 | 56.2% | 0.94 [0.75, 1.16] |
| Total events | 986 | | 1940 | | | |
| Heterogeneity: Tau² = 0.02; Chi² = 1.92, df = 1 (P = 0.17); I² = 48% | | | | | | |
| Test for overall effect: Z = 0.60 (P = 0.55) | | | | | | |
| Total (95% CI) | | 20039 | | 30816 | 100.0% | 1.04 [0.92, 1.17] |
| Total events | 1515 | | 2783 | | | |
| Heterogeneity: Tau² = 0.01; Chi² = 7.15, df = 4 (P = 0.13); I² = 44% | | | | | | |
| Test for overall effect: Z = 0.64 (P = 0.52) | | | | | | |
| Test for subgroup differences: Chi² = 2.57, df = 2 (P = 0.28), I² = 22.3% | | | | | | |

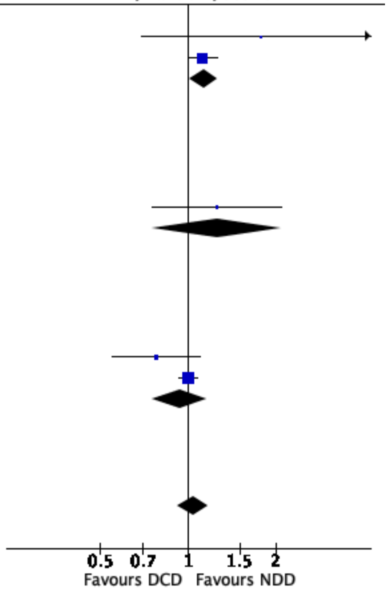


## Delayed graft Function

| Study or Subgroup | DCD events Events | DCD events Total | NDD events Events | NDD events Total | Weight | Risk Ratio M-H, Random, 95% CI |
|---|---|---|---|---|---|---|
| Bell 2019 | 223 | 468 | 177 | 905 | 7.2% | 2.44 [2.07, 2.87] |
| Chen 2017 | 58 | 258 | 6 | 59 | 0.4% | 2.21 [1.00, 4.88] |
| Kostakis 2020 | 794 | 1884 | 1194 | 5509 | 19.6% | 1.94 [1.81, 2.09] |
| Lafuente 2016 | 15 | 29 | 7 | 26 | 0.5% | 1.92 [0.93, 3.96] |
| Lia 2021 | 7452 | 18354 | 25335 | 117290 | 32.5% | 1.88 [1.84, 1.92] |
| Minambres 2020 | 8 | 19 | 12 | 67 | 0.4% | 2.35 [1.13, 4.90] |
| Molina 2019 | 174 | 237 | 110 | 237 | 7.7% | 1.58 [1.35, 1.85] |
| Perez-Saez 2021 | 111 | 277 | 132 | 575 | 4.8% | 1.75 [1.42, 2.15] |
| Trotter 2018 | 2377 | 5899 | 2778 | 12741 | 26.7% | 1.85 [1.77, 1.93] |
| Yuan 2014 | 17 | 101 | 2 | 50 | 0.1% | 4.21 [1.01, 17.50] |
| Total (95% CI) | | 27526 | | 137459 | 100.0% | 1.89 [1.80, 1.99] |
| Total events | 11229 | | 29753 | | | |
| Heterogeneity: Tau² = 0.00; Chi² = 18.03, df = 9 (P = 0.03); I² = 50% | | | | | | |
| Test for overall effect: Z = 25.39 (P < 0.00001) | | | | | | |

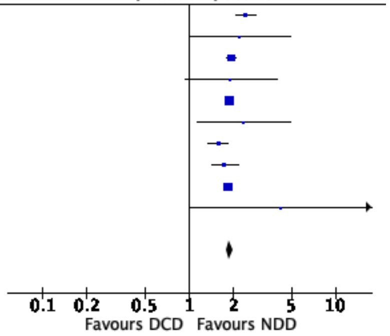

Supplement: Supplementary file 2 [file DataSheet1.pdf]
